# Modular Calcium‐Responsive and CD9‐Targeted Phospholipase System Enhancing Endosomal Escape for DNA Delivery

**DOI:** 10.1002/advs.202410815

**Published:** 2025-02-25

**Authors:** Alexander Klipp, Christina Greitens, David Scherer, Alexander Elsener, Jean‐Christophe Leroux, Michael Burger

**Affiliations:** ^1^ Department of Chemistry and Applied Biosciences ETH Zürich Vladimir‐Prelog‐Weg 3 Zürich 8093 Switzerland

**Keywords:** calcium‐responsive system, CD9 targeting, DNA delivery, endosomal escape, phospholipase C

## Abstract

Gene delivery systems must overcome multiple barriers, with endosomal escape representing a prominent obstacle. We have previously shown that a bacterial phospholipase C (PLC) enabled endosomal escape of a non‐viral protein‐based DNA delivery system termed TFAMoplex. Building upon this, this work introduces a calcium‐responsive system designed to enhance endosomal escape through non‐covalent capturing of PLC to the TFAMoplex followed by its release within endosomes and nanobody‐mediated targeting to the endosomal membrane. This approach leads to improved TFAMoplexes enabling transfection of HeLa cells in full serum with a half maximal effective concentration (EC_50_) of less than 200 ng DNA per mL serum, using only 5 nM PLC. Particularly, the modular capture, release and targeting system could potentially be adapted to other delivery agents previously constrained by poor endosomal escape. These findings present a promising strategy to achieve efficient endosomal escape, offering prospects for improved delivery of macromolecules, in particular nucleic acids.

## Graphical abstract

A modular endosomal escape system boosts transfection efficiency of a DNA delivery agent. This system comprises a phospholipase fused to a split‐calbindin domain and a nanobody targeting CD9. In the extracellular space, the phospholipase is tightly bound to protein‐DNA complexes, released within endosomes at low calcium concentrations and recruited to the membrane by binding to CD9, resulting in efficient endosomal destabilization.

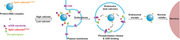

## 1 Introduction

Gene therapy holds the potential to change the future of medicine by enabling curative treatment of numerous human diseases. Successful gene therapy depends on the introduction of nucleic acids, for example, DNA, into target cells. Once inside the cells, the genetic payload can exert specific functions, such as editing, adding, replacing, and regulating a target gene involved in a disease. The delivery of nucleic acids into cells is, however, difficult and still poses a substantial obstacle for gene therapy.^[^ ^1^, ^2^, ^3^, ^4^, ^5^, ^6^ ^]^

Gene delivery vectors harboring nucleic acids are usually taken up by cells via endocytosis.^[^ ^7^, ^8^ ^]^ The endocytic vesicles containing the genetic payload undergo maturation from early to late endosomes and, eventually, to lysosomes, where the engulfed cargo is degraded by hydrolytic enzymes. During endosomal maturation the pH decreases gradually reaching a value of ≈4–5 in the lysosomes.^[^ ^9^, ^10^, ^11^ ^]^ Moreover, the calcium concentration also decreases inside the endosomal compartment reaching the low µM range. This creates a steep gradient compared to the extracellular space, where the pH is 7.4 and the calcium concentration ≈2 mM.^[^ ^12^ ^]^ In general, successful gene delivery systems share the requirement to evade the endosomal compartment and, therewith, lysosomal degradation. This, however, still represents one of the major hurdles, considering that most non‐viral systems exhibit only poor endosomal escape.^[^ ^13^, ^14^, ^15^ ^]^

Viruses as well as certain bacteria evolved sophisticated mechanisms to penetrate cells.^[^ ^16^, ^17^, ^18^ ^]^ One common pathogenic strategy is the disruption of endosomes by hydrolysis of phospholipids. This is, for example, applied by the parvovirus, which is thought to use a phospholipase A_2_ domain for this purpose.^[^ ^19^ ^]^ Also distinct bacteria, such as *Listeria monocytogenes* (*L. m*.) and *Clostridium perfringens* (*C. p*.), secrete phospholipase C (PLC) to disrupt membranes by phospholipid cleavage.^[^ ^20^, ^21^, ^22^ ^]^ Interestingly, the *L. m*. broad‐range PLC exhibits maximal activity at pH 5.5, restricting its activity to acidic compartments, such as the phagosome.^[^ ^23^ ^]^ Many strategies to enter cells are, moreover, dependent on specific triggers, including cleavage by endosomal host proteases^[^ ^24^, ^25^, ^26^ ^]^ or pH‐induced conformational changes in pathogenic proteins.^[^ ^27^, ^28^, ^29^ ^]^ Sophisticated drug delivery systems try to mimic these stimulus‐triggered responses inside endosomes, aiming to increase endosomal escape efficiency. Examples include lipid nanoparticles (LNPs) containing ionizable lipids,^[^ ^30^, ^31^ ^]^ cathepsin B‐dependent delivery systems^[^ ^32^ ^]^ as well as pH‐responsive nanoparticles^[^ ^33^ ^]^ and micelles.^[^ ^34^, ^35^ ^]^

A non‐viral DNA delivery system termed TFAMoplex, which was recently developed in our laboratory, was shown to efficiently transfect cells.^[^ ^36^ ^]^ TFAMoplexes are protein‐DNA complexes of ≈100 nm in size and depend on the human mitochondrial transcription factor A (TFAM) for compacting plasmid DNA (pDNA). Formation of those complexes is carried out by combining two TFAM fusion proteins at equimolar concentrations with pDNA. One fusion protein comprises TFAM fused covalently to the *L. m*. broad‐range phospholipase C (PLC‐TFAM), which is crucial for enabling endosomal escape via phospholipid hydrolysis. The second fusion protein is composed of the double mutant TFAM^A105C, V109C^ fused covalently to the human vaccinia‐related kinase 1 (cysTFAM‐VRK1). VRK1 is a serine/threonine‐protein kinase that phosphorylates the protein barrier‐to‐autointegration factor (BAF). BAF was shown to complex exogenous DNA in the cytoplasm of mammalian cells and, eventually, sequester it within endoplasmic reticulum (ER)‐derived membrane cages.^[^ ^37^, ^38^, ^39^, ^40^, ^41^ ^]^ To prevent delivered DNA from being retained in the cytoplasm, VRK1 was incorporated into the TFAMoplex, since VRK1‐mediated phosphorylation of BAF was shown to inactivate its DNA‐binding function.^[^ ^42^ ^]^ The inclusion of VRK1 into the complexes resulted in significantly increased transfection efficiency. However, TFAMoplexes containing a kinase‐dead mutant of VRK1 (VRK1^D177A^) exhibited the same transfection efficiency as those harboring active VRK1. The mechanism by which VRK1 enhances transfection remains unknown.^[^ ^36^ ^]^ Moreover, incorporating the cysTFAM variant was essential to achieve efficient transfection in pure serum, mimicking in vivo conditions encountered after intravenous injection. TFAMoplexes formed only with wildtype (wt) TFAM did not transfect cells under those conditions.^[^ ^36^ ^]^ Therefore, we decided to only use cysTFAM in this study, which will be hereafter referred to as TFAM for simplicity.

We hypothesized that the PLCs in the TFAMoplex are sterically hindered from reaching the endosomal membrane, limiting the efficiency of endosomal escape. This hypothesis is supported by in vitro experiments showing that PLC's activity is reduced by ≈50% when inside the TFAMoplex compared to free PLC.^[^ ^36^ ^]^ We aimed to overcome this problem by releasing PLC from the TFAMoplex in an endosome‐specific manner together with directing the enzyme to the endosomal membrane for increasing its phospholipid hydrolyzing potency.

**Figure 1:**
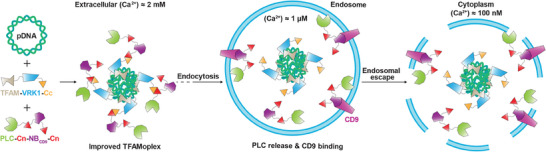
Schematic concept for improving PLC‐mediated endosomal escape of TFAMoplexes. Formation of TFAMoplexes is carried out by incubating TFAM‐VRK1‐Cc with pDNA. PLC is captured at extracellular calcium concentrations by two Cn domains to the TFAMoplex and directed via the NB_CD9_ moiety to the endosomal membrane. Following endocytosis, the low calcium concentration inside endosomes results in the release of PLC and its recruitment to the membrane, eventually leading to endosomal escape mediated by phospholipid hydrolysis. This version of the TFAMoplex is referred to as capture, release and targeting2 (CRT2)‐TFAMoplex. Cc, Cal9k C‐terminal part; Cn, Cal9k N‐terminal part.

Here, we present a calcium‐responsive capture and release system combined with a membrane‐targeting mechanism of PLC to enable efficient endosomal escape, even under stringent transfection conditions (**Figure** **1**). The capture and release mechanism is based on the calcium‐binding protein calbindin‐D9k (Cal9k), which is a small globular protein (≈9 kDa).^[^ ^43^ ^]^ Bovine Cal9k was previously split into two parts for studying protein fragment complementation.^[^ ^44^ ^]^ It was shown that the two split parts exhibit very high binding affinity (K_D_ = 3 pM) in the presence of mM calcium concentrations, while the binding was very weak in the absence of calcium. The human version of the split Cal9k linker system, which is highly homologous to the bovine version (88.6% sequence identity), was applied to TFAMoplexes to enable non‐covalent capturing of PLC to the complexes. Once inside the endosomes, the decreasing calcium concentration should trigger PLC release from the complex, thereby augmenting its activity. To additionally ensure that the released PLC is localizing to the endosomal membrane, we coupled it to the nanobody 4c8 targeting the extracellular loop 2 of human CD9 (NB_CD9_).^[^ ^45^ ^]^ The tetraspanin CD9 is ubiquitously expressed in mammalian cells and enriched within endosomes and at the plasma membrane of human cells.^[^ ^46^ ^]^

We demonstrate that applying the capture, release and membrane targeting system to PLC increases its potency. Moreover, employing this modular system to TFAMoplexes significantly enhances transfection efficiency.

## 2 Results

### 2.1 A Split Protein System for Calcium‐Responsive Capture and Release

**Figure 2:**
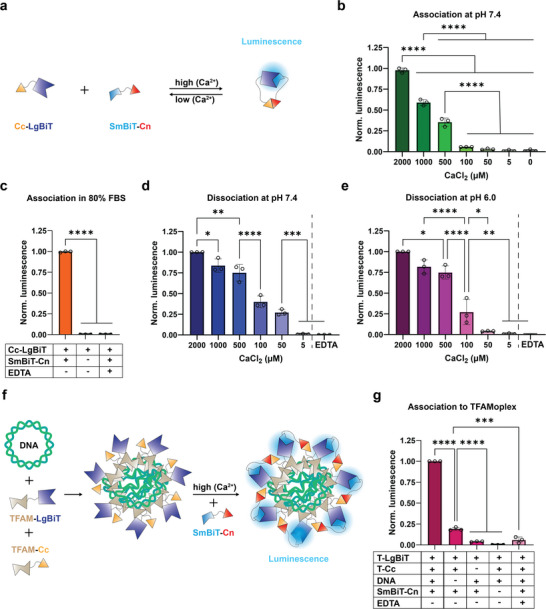
Split Cal9k as calcium‐sensitive capture and release system for gene delivery. a) Schematic representation of experimental workflow for studying split Cal9k association by split NanoLuc complementation. SmBiT‐Cn was incubated with Cc‐LgBiT at different CaCl_2_ concentrations and generation of luminescence was recorded. b) Split Cal9k association at pH 7.4 (2 nM equimolar proteins). c) Split Cal9k association in 80% FBS ± SmBiT‐Cn and ± 2 mM EDTA (10 nM equimolar proteins). d,e) Dissociation experiments were conducted by incubating SmBiT‐Cn with Cc‐LgBiT at 2 mM CaCl_2_ for 30 min ensuring binding and split NanoLuc complementation. The sample was then diluted to different CaCl_2_ concentrations ± 2 mM EDTA and luminescence was recorded (0.2 nM equimolar proteins). d) Dissociation at pH 7.4. e) Dissociation at pH 6.0. f) Schematic representation of experimental workflow for studying split Cal9k association to the TFAMoplex. TFAMoplexes were formed by incubating plasmid DNA with TFAM‐LgBiT and TFAM‐Cc for 30 min. Then, SmBiT‐Cn was added to the sample and luminescence was recorded. g) Split Cal9k association to the TFAMoplex in 80% FBS with different construct combinations and ± 6 mM EDTA (0.9 µM TFAM‐LgBiT, 0.7 µM TFAM‐Cc, 10 ng µL^−1^ DNA, 0.35 µM SmBiT‐Cn). Bar plots of association experiments display values at plateau phase. Bar plots of dissociation experiments display values recorded after 1 min. Data were normalized within each biological replicate. Data shown as mean ± SD of N = 3 independent experiments, each performed in technical triplicates. Data was analyzed using one‐way ANOVA with Tukey's multiple comparison test. Statistical significance is specified with *p < 0.05, **p < 0.01, ***p < 0.001, ****p < 0.0001.

First, we investigated binding of the two split Cal9k parts by split NanoLuc complementation.^[^ ^47^ ^]^ The N‐terminal part of Cal9k (Cn) was fused to maltose binding protein (MBP) connected to Small NanoBiT (MBP‐SmBiT‐Cn) and the C‐terminal part of Cal9k (Cc) was inserted between MBP and Large NanoBiT (MBP‐Cc‐LgBiT). Note that MBP, which served as mock passenger protein, is omitted for simplicity hereafter. Hence, the two fusion constructs are referred to as SmBiT‐Cn and Cc‐LgBiT (**Figure** **2**a). SmBiT and LgBiT display very low binding affinity (K_D_ = 190 µM), and only generate luminescence if they are actively combined.^[^ ^47^ ^]^ At pH 7.4, binding of SmBiT‐Cn to Cc‐LgBiT was detected at calcium concentrations above 0.5 mM with maximal binding occurring at 2 mM calcium (Figure 2b). No binding could be detected at calcium concentrations encountered in the endosomal compartment (low µM range). To better mimic in vivo conditions, we also tested binding in 80% fetal bovine serum (FBS) (Figure 2c), which naturally contains calcium concentrations in the low mM range.^[^ ^48^ ^]^ In FBS, binding of Cc to Cn was detected. As expected, no binding of the two Cal9k split parts could be measured when the calcium chelating agent EDTA was added to the sample.

Subsequently, we assessed whether the binding of Cn to Cc was reversible upon calcium removal, and on which time scale the dissociation happened, considering that the average time through the endosomal compartment is ≈30 min.^[^ ^9^ ^]^ For this purpose, SmBiT‐Cn was incubated with Cc‐LgBiT at 2 mM CaCl_2_ for 30 min to ensure association. Subsequently, the sample was diluted in buffers containing different CaCl_2_ concentrations and luminescence signal was recorded. At pH 7.4 and only 1 min after dilution, the luminescence signal decreased with decreasing final calcium concentrations, indicating the dissociation of the two protein fragments (Figure 2d). No signal was detected at 5 µM CaCl_2_, as encountered in the endosomes. Taking into account that the pH value in the endosomal compartment is acidic,^[^ ^9^ ^]^ we also investigated dissociation at pH 6.0 (Figure 2e). Dissociation of Cn from Cc showed a similar trend at pH 6.0 versus 7.4, with the difference that no significant signal could be detected already at 50 µM CaCl_2_. This behavior at acidic pH is favorable for quickly releasing PLC in the endosomal compartment.

Next, we investigated whether the calcium‐sensitive linkage could be employed with the TFAMoplex system. Therefore, pDNA was incubated with TFAM fused to Cc (TFAM‐Cc) as well as TFAM fused to LgBiT (TFAM‐LgBiT) (Figure 2f). After complex formation, SmBiT‐Cn was added. In this assay, luminescence should only be generated if the luciferase parts are brought together on the TFAMoplex. Again, capturing of SmBiT‐Cn onto TFAMoplexes was measured in 80% FBS. Luminescence data indicated that SmBiT‐Cn associated to the TFAMoplexes (Figure 2g). In the absence of DNA, TFAMoplexes could not form and only weak luminescence was detected. This could be explained by non‐specific TFAM interactions in 80% FBS mediated by serum components. Adding EDTA abolished the signal, indicative of dissociated SmBiT‐Cn. To test whether SmBiT‐Cn could bind non‐specifically to the TFAMoplex, TFAMoplexes were prepared without TFAM‐Cc. No significant luminescence could be detected in this case, indicating little unspecific binding of SmBiT‐Cn to the TFAMoplex. Complementation curves of the described luminescence experiments can be found in Figure S1, Supporting Information.

In summary, these data demonstrate that the split Cal9k system can capture a protein at conditions found in the extracellular space (neutral pH and high calcium concentration) and quickly release it once encountering endosomal conditions (acidic pH and low calcium concentration). Importantly, we showed that capturing of a protein partner can also be achieved onto protein‐DNA complexes, such as the TFAMoplexes, and in 80% FBS.

### 2.2 Harnessing CD9 for Membrane Targeting

**Figure 3:**
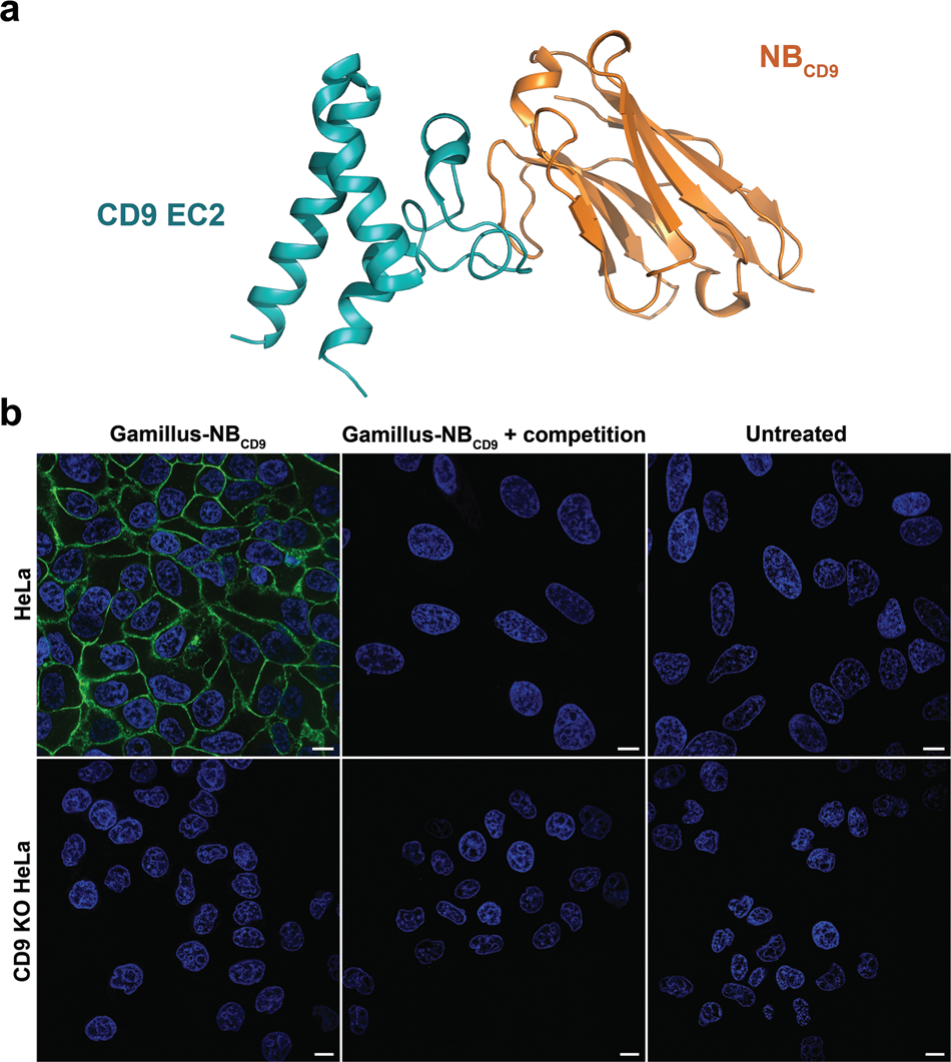
NB_CD9_ binds specifically to CD9 on HeLa cells. a) Binding mode of NB_CD9_ (orange) to extracellular loop 2 (EC2) of CD9 (teal) depicted in cartoon representation (PDB ID = 6Z20).^[^ ^45^ ^]^ b) Representative images investigating binding of Gamillus‐NB_CD9_ on HeLa and CD9 KO HeLa cells. Gamillus‐NB_CD9_ (100 nM) was applied for 30 min on cells. For competition experiments, 2.5 µM unlabeled NB_CD9_ was incubated 10 min before addition of Gamillus‐NB_CD9_ on the cells. Nuclei were stained with Hoechst (blue). Single z‐slices in the center of the cells are shown. Scale bar 10 µm.

We aimed to additionally target PLC to the membrane via NB_CD9_ (**Figure** **3**a) in order to increase its potency. This strategy was inspired by nature, where one of the most toxic PLCs (α‐toxin from *Clostridium perfringens*) contains a membrane‐recruiting C2 domain for its very high phospholipid hydrolyzing activity.^[^ ^22^ ^]^ Moreover, we expected added benefits by combining the targeting with the capture and release of PLC. For investigating binding to cells, we created a NB_CD9_ fusion construct with Gamillus,^[^ ^49^ ^]^ a pH‐stable green fluorescent protein (GFP) (Gamillus‐NB_CD9_) as well as the free NB_CD9_ as control. Cell association experiments using Gamillus‐NB_CD9_ on HeLa cells confirmed binding of the nanobody to CD9, which was abolished by competition with unlabeled NB_CD9_ or when using CD9 knock‐out (KO) HeLa cells demonstrating the activity and specificity of the NB_CD9_ (Figure 3b).

### 2.3 A Combined Capture, Release and Membrane Targeting System for Increased PLC Potency

After characterization of the capture and release system and selection of the ubiquitously expressed CD9 as target for membrane recruitment of PLC inside the endosomal compartment, we combined those two mechanisms to increase the potency of PLC in promoting endosomal escape. Therefore, we created a fusion construct consisting of PLC fused to two Cn moieties flanking the NB_CD9_ (PLC‐Cn‐NB_CD9_‐Cn). We assumed that two Cn moieties would ensure a strong coupling, since efficient transfection requires the PLC to remain within the TFAMoplex until reaching the endosomal compartment. The possibility that PLC‐Cn‐NB_CD9_‐Cn could bind itself rather than to the Cc partner is unlikely given that the K_D_ for heterodimerization of the two bovine split Cal9k partners (i.e., Cn with Cc) is 6 orders of magnitude lower than the K_D_ for homodimerization (i.e., Cn with Cn).^[^ ^44^ ^]^

To assess coupling behavior of PLC‐Cn‐NB_CD9_‐Cn to Cc, we created the fusion construct MBP‐SmBiT‐Cn‐NB_CD9_‐Cn and studied its binding to LgBiT‐Cc by split NanoLuc complementation. MBP is omitted for clarity hereafter. Considering that LgBiT‐Cc can bind to both Cn domains of PLC‐Cn‐NB_CD9_‐Cn but only one LgBiT can complement with SmBiT, we used a fourfold excess of LgBiT‐Cc in association and dissociation experiments to fully saturate PLC‐Cn‐NB_CD9_‐Cn. Split NanoLuc experiments with the double Cn construct were compared to SmBiT‐Cn. In 80% FBS, binding resulted in 1.3‐fold increased signal compared to SmBiT‐Cn (Figure S2a, Supporting Information), which could indicate enhanced binding in FBS when using two Cn moieties. Dissociation experiments at pH 7.4 and 6.0 exhibited the same trend as SmBiT‐Cn, with no detectable luminescence after dilution to µM calcium concentrations (Figure S2b,c, Supporting Information). Furthermore, split NanoLuc experiments investigating capturing of SmBiT‐Cn‐NB_CD9_‐Cn to TFAMoplexes (performed with twofold excess of the Cc partner) showed no significant difference compared to SmBiT‐Cn (Figure S2d, Supporting Information). These data indicate that the fusion construct containing two Cn moieties flanking the NB_CD9_ binds to Cc in complex environments, such as FBS, and is well suited for tightly capturing and releasing PLC in endosomes.

**Figure 4:**
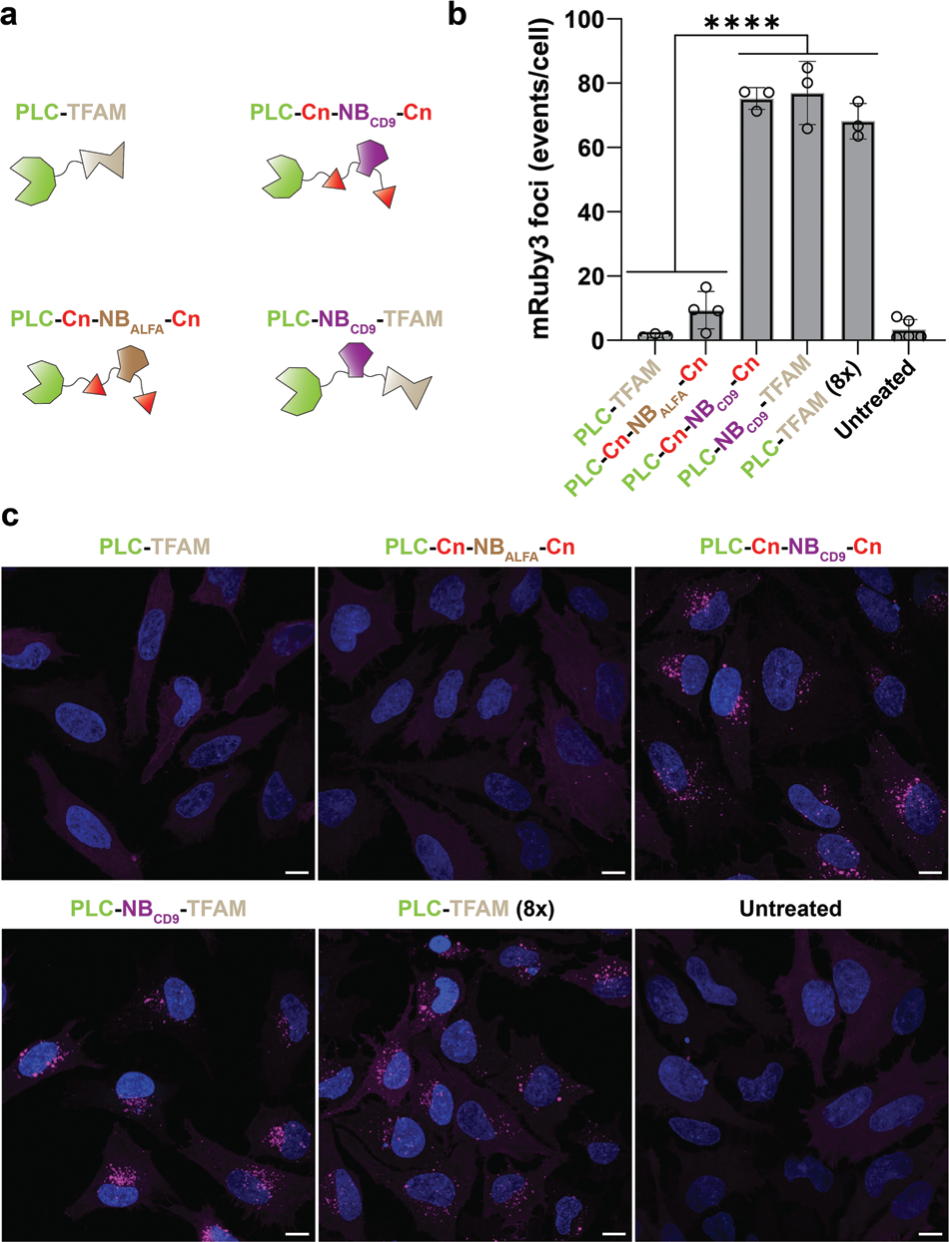
Targeting PLC to the membrane enhances endosomal disruption. a) Schematic representation of the various PLC constructs. b) Quantification of endosomal disruption in HeLa‐Gal8‐mRuby3 cells induced by the different PLC constructs. Each data point represents the average amount of mRuby3 events per cell in one image. Data shown as mean ± SD and analyzed using one‐way ANOVA with Tukey's multiple comparison test. Statistical significance is specified with ****p < 0.0001. c) Representative images displaying endosomal rupture. Endosomal disruption is visualized by punctate mRuby3 fluorescence (pseudocolored magenta). All proteins were applied at 15 nM for 3 h to the cells. PLC‐TFAM was additionally applied at 120 nM (8×). Images show z‐projections of maximum intensities. Nuclei were stained with Hoechst (blue). Scale bar 10 µm.

The next aim was to investigate whether PLC‐Cn‐NB_CD9_‐Cn would exhibit increased endosomal permeation potency compared to PLC‐TFAM. As control, we replaced the NB_CD9_ with a nanobody targeting the ALFA tag^[^ ^50^ ^]^ (PLC‐Cn‐NB_ALFA_‐Cn). The ALFA tag represents an artificial epitope which is not present in cells. Additionally, we created the fusion construct PLC‐NB_CD9_‐TFAM, where PLC is fused directly to the NB_CD9_ connected to TFAM, as control for capture and release. The fusion proteins are represented schematically in **Figure** **4**a. In vitro assays investigating phospholipase activity revealed that all PLC fusion constructs performed similarly (Figure S3, Supporting Information). This was expected, considering that the activity assay is performed with an artificial substrate lacking CD9 as target for the nanobody. To test the ability of the fusion proteins in promoting endosomal disruption, we applied the free proteins, that is, only proteins without DNA, on HeLa‐Gal8‐mRuby3 cells.^[^ ^51^, ^52^ ^]^ This stable cell line overexpresses cytoplasmic galectin‐8 fused to the fluorescent protein mRuby3 (Gal8‐mRuby3), which is evenly dispersed throughout the cytoplasm at cellular homeostasis. Upon endosomal disruption, Gal8‐mRuby3 is recruited to the inner leaflet of endosomes by binding to specific sugars and resulting in punctate red fluorescence.^[^ ^51^, ^52^ ^]^ Confocal microscopy analysis revealed that applying 15 nM of the PLC fusion constructs containing NB_CD9_ to HeLa‐Gal8‐mRuby3 cells induced pronounced endosomal permeation (Figure 4b,c). In contrast, PLC‐TFAM and PLC‐Cn‐NB_ALFA_‐Cn promoted only very limited or no endosomal disruption at this concentration. For PLC‐TFAM, an eight times higher concentration (120 nM) was necessary for inducing endosomal rupture with fluorescent puncta comparable to the ones obtained by applying the NB_CD9_ constructs at 15 nM. PLC fusion constructs displayed drastic differences when applied on cells, where solely the constructs containing NB_CD9_ were able to promote pronounced endosomal disruption at a low concentration. Therefore, these data support the hypothesis that targeting PLC to the membrane, its place of action, improves endosomal disruption.

### 2.4 Improving TFAMoplex Potency

Having shown that the calcium‐sensitive capture and release mechanism works as desired in vitro and that targeting CD9 enhances PLC potency in permeating endosomes, the next step was to couple this system to the TFAMoplex. This approach is referred to as capture, release and targeting (CRT)‐TFAMoplex. In the previously published TFAMoplex system (using covalent PLC‐wtTFAM) the final applied protein concentration in transfection studies was 32 nM (16 nM PLC‐wtTFAM and 16 nM TFAM‐VRK1) and the final DNA concentration was 200 ng mL^−1^. Note that transfection was abolished when complexes without PLC or with a phospholipase‐dead mutant were applied.^[^ ^36^ ^]^ For comparison, we used the same composition for the CRT‐TFAMoplex, with the exception that the 16 nM PLC‐wtTFAM were replaced by 14 nM TFAM‐Cc and 2 nM TFAM, and PLC‐Cn‐NB_CD9_‐Cn was included. The 2 nM extra TFAM were included to keep the total TFAM concentration equal in all tested TFAMoplex compositions, since changing the total TFAM concentration could itself influence transfection efficiency. Additionally, we aimed to simplify the CRT‐TFAMoplexes, which contained 4 protein components. Therefore, we covalently fused the Cc moiety to the C‐terminus of TFAM‐VRK1 (TFAM‐VRK1‐Cc), creating a system consisting only of TFAM‐VRK1‐Cc, PLC‐Cn‐NB_CD9_‐Cn and pDNA (termed CRT2‐TFAMoplexes) (Figure 1). By this, we reduced the number of protein components and increased the total VRK1 concentration in the complexes, which was hypothesized to further enhance the transfection efficiency. As control, TFAMoplexes consisting of PLC‐TFAM and TFAM‐VRK1 were used (termed original TFAMoplex). Note that for all experiments investigating transfection efficiency in this study, the TFAMoplexes were formed in ≥ 80% FBS and cells were transfected in 99% FBS for only 30 min, representing particularly challenging conditions.

The mean particle hydrodynamic diameters of the original TFAMoplexes, as well as the CRT‐ and CRT2‐TFAMoplexes in HEPES buffer at pH 7.4 containing 150 mM KCl ranged from 98 to 120 nm (Figure S4, Supporting Information), which is comparable to the sizes of previous TFAMoplex systems.^[^ ^36^, ^53^ ^]^ When measured in the same buffer supplemented with 2 mM CaCl_2_, as it is required for the recruitment of PLC‐Cn‐NB_CD9_‐Cn, the complexes exhibited slightly smaller mean diameters ranging from 90 to 100 nm, which could be related to Ca^2+^ ions binding to the negatively charged phosphate backbone of DNA thereby compacting the particles. Moreover, gel mobility shift assays confirmed similar DNA binding behavior among the three systems (Figure S5, Supporting Information).

**Figure 5:**
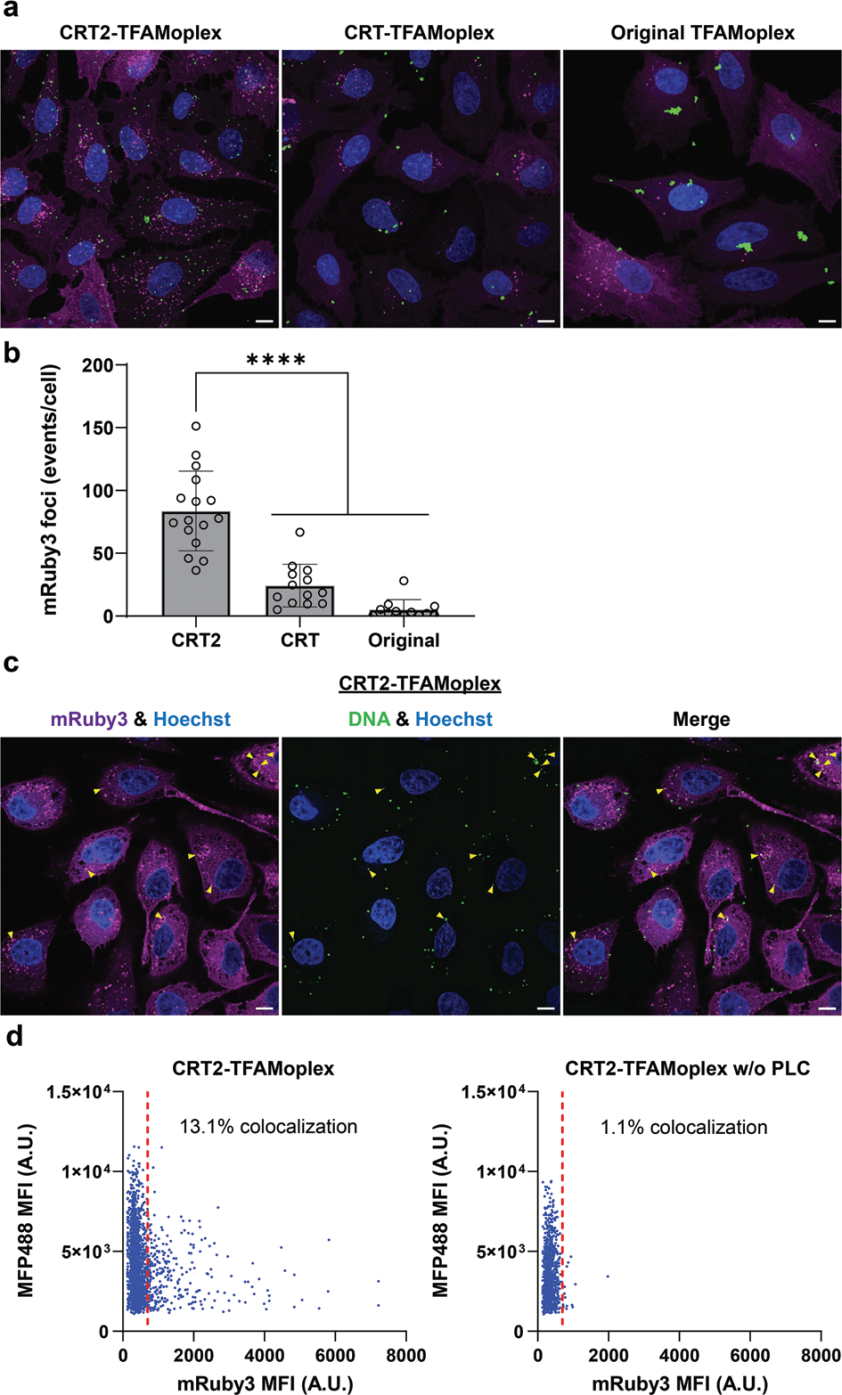
Combining TFAMoplexes with capture, release and targeting of PLC enhances endosomal disruption. a) Representative images displaying endosomal rupture (punctate mRuby3 fluorescence) in HeLa‐Gal8‐mRuby3 cells induced by the different TFAMoplex systems using MFP488‐labelled DNA. b) Quantification of endosomal rupture. Each data point represents the average amount of mRuby3 events per cell in one image. Data shown as mean ± SD and analyzed using one‐way ANOVA with Tukey's multiple comparison test. Statistical significance is specified with ****p < 0.0001. c) Colocalization of MFP488‐labelled DNA with endosomal disruption induced by CRT2‐TFAMoplexes (white spots indicated with yellow arrowheads). Images show z‐projections (a) and a z‐slice (c). d) Quantification of TFAMoplex colocalization with disrupted endosomes (segmented MFP488 signal and corresponding mRuby3 signal). Threshold for colocalization (dotted red line) was set to 696.5, corresponding to the mean mRuby3 mean fluorescence intensity (MFI) of CRT2‐TFAMoplexes w/o PLC plus three times SD. A total of 160 cells and 144 cells were analyzed for CRT2‐TFAMoplexes and CRT2‐TFAMoplexes w/o PLC, respectively. CRT2‐TFAMoplexes (10 nM PLC‐Cn‐NB_CD9_‐Cn, 64 nM TFAM‐VRK1‐Cc), CRT‐TFAMoplexes (4 nM PLC‐Cn‐NB_CD9_‐Cn, 28 nM TFAM‐Cc, 4 nM TFAM, 32 nM TFAM‐VRK1), original TFAMoplexes (32 nM PLC‐TFAM, 32 nM TFAM‐VRK1). All complexes were added to cells in 99% FBS for 1 h with final 400 ng mL^−1^ DNA. MFP488 is displayed in green, mRuby3 is pseudocolored magenta. Nuclei stained with Hoechst (blue). Scale bar 10 µm.

To study endosomal colocalization, HeLa cells containing GFP‐Rab5a labelled early endosomes were transfected with CRT2‐TFAMoplexes prepared with Cy3‐labelled DNA. Analysis by confocal microscopy revealed colocalization of CRT2‐TFAMoplexes with early endosomes (Figure S6, Supporting Information). Next, endosomal disruption induced by the different TFAMoplex systems was investigated using optimized protein ratios. MFP488‐labelled pDNA was used for complex formation to enable DNA visualization. Note that the total concentrations applied were twice as high as those used in standard TFAMoplex transfection experiments (200 ng mL^−1^ DNA), due to the sensitivity limit of the Gal8‐mRuby3 assay. The complexes were applied to the cells in 99% FBS for 1 h. Endosomal permeation induced by the three TFAMoplex versions is shown in **Figure** **5**, while the control experiments for the same assay are shown in Figure S7, Supporting Information. Confocal images revealed endosomal disruption for complexes containing NB_CD9_ as well as for the original TFAMoplexes. The CRT2‐TFAMoplexes induced pronounced endosomal destabilization while both the original and CRT‐TFAMoplexes showed less effect (Figure 5a,b). Interestingly, the original TFAMoplex system was prone to aggregation (large green DNA spots) at the applied concentration (final 400 ng mL^−1^ DNA), while the other systems did not display aggregation. However, no aggregation was observed when the original TFAMoplexes were applied at the same concentration as for transfection experiments (200 ng mL^−1^) (Figure S8, Supporting Information). Endosomal permeation could also be detected when PLC‐Cn‐NB_CD9_‐Cn was added to the cells in a free form, that is, with TFAMoplexes containing TFAM instead of TFAM‐Cc (Figure S7, Supporting Information). This was consistent with the Gal8‐mRuby3 experiment using free proteins (Figure 4). Moreover, complexes harboring the non‐targeted PLC‐Cn‐NB_ALFA_‐Cn showed only very limited permeation of endosomes.

Remarkably, applying the CRT2‐TFAMoplexes to the cells resulted in extensive colocalization of labelled DNA with fluorescent mRuby3 puncta, indicating successful permeation of endosomes harboring TFAMoplexes (Figure 5c,d). The colocalization was quantified using CRT2‐TFAMoplexes without PLC as negative control (exhibiting no endosomal permeation) and amounted to 13.1% for CRT2‐TFAMoplexes (Figure 5d). Temporal dynamics of colocalization with CRT2‐TFAMoplexes, which was studied with live‐cell time‐lapse imaging, revealed that detectable endosomal rupture and colocalization occurred after 40–50 min (Movie S1 and Figure S9, Supporting Information). These findings provide evidence that the complexes enter the cytoplasm of cells via endosomal escape rather than via other routes, such as by directly hydrolyzing the cell surface membrane. This aligns with the fact that PLC exhibits low activity at neutral pH and highest activity at acidic pH, as encountered in the endosomal compartment.^[^ ^23^ ^]^

Nuclear colocalization was studied using CRT2‐TFAMoplexes containing TFAM‐Gamillus and Cy3‐labelled pDNA (Figure S10, Supporting Information). While Cy3‐DNA signal could occasionally be detected in a Hoechst stained region, its precise localization, whether inside the nucleus or associated with the nuclear membrane inside BAF clusters, remained unclear, due to limitations in microscopy resolution.^[^ ^54^ ^]^ Therefore, the next step was to transfect cells and quantify reporter gene expression as a more reliable indicator of nuclear DNA delivery.

**Figure 6:**
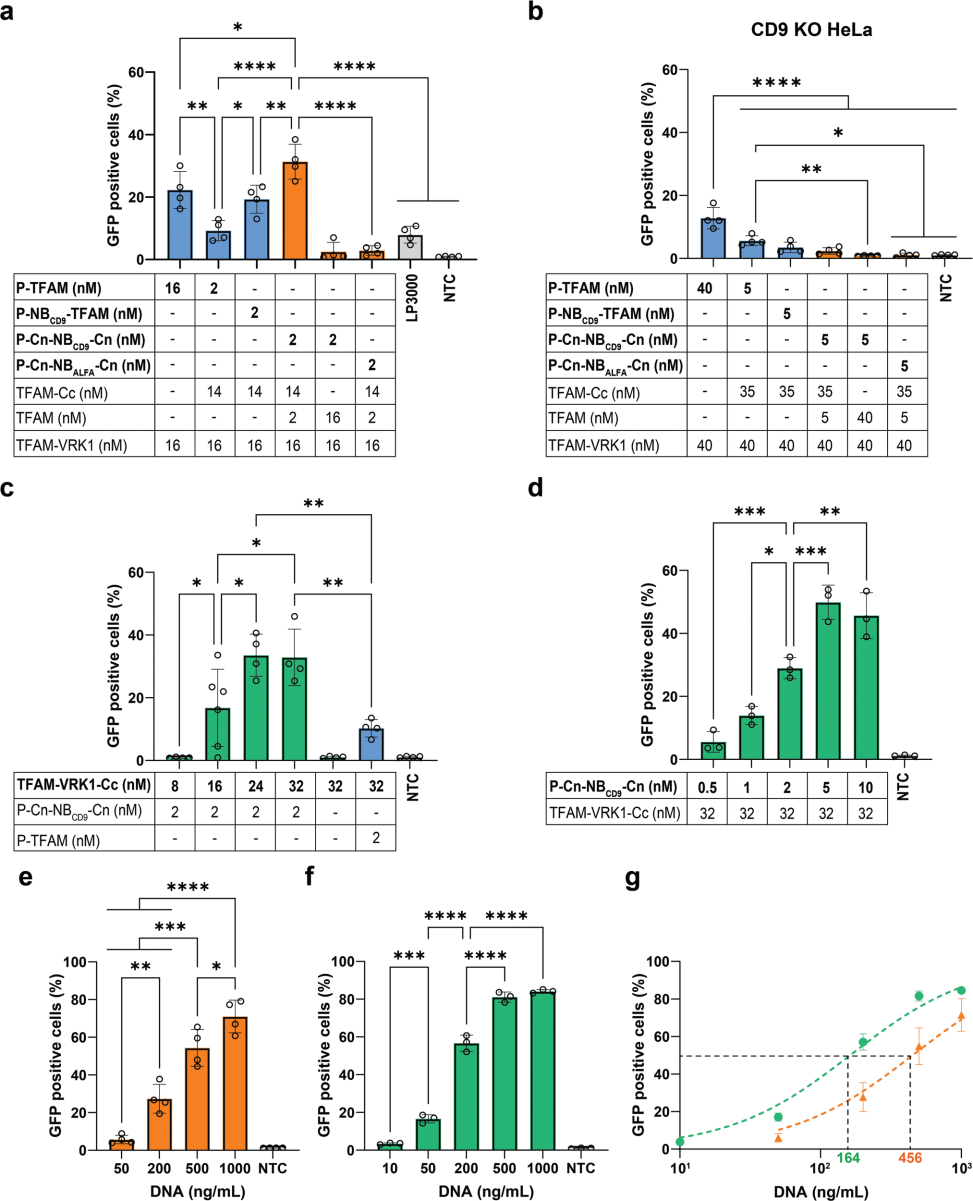
Applying the capture, release and targeting system to TFAMoplexes enhances transfection efficiency. a) Transfection of HeLa and b) CD9 KO HeLa cells with different TFAMoplex systems or with Lipofectamine 3000 using indicated protein concentrations and 200 ng mL^−1^ pDNA (HeLa) and 500 ng mL^−1^ pDNA (CD9 KO HeLa). c) Transfection of HeLa cells with the CRT2‐TFAMoplex system titrating TFAM‐VRK1‐Cc and TFAMoplexes containing PLC‐TFAM as control using 200 ng mL^−1^ pDNA and indicated final protein concentrations on the cells. d) Transfection with CRT2‐TFAMoplexes titrating PLC‐Cn‐NB_CD9_‐Cn using 200 ng mL^−1^ pDNA and indicated protein concentrations on HeLa cells. e) CRT‐TFAMoplex dose escalation using PLC‐Cn‐NB_CD9_‐Cn (0.1–10 nM), TFAM‐Cc (0.7–70 nM), TFAM (0.1–10 nM), TFAM‐VRK1 (0.8–80 nM) and indicated DNA concentrations on HeLa cells. f) CRT2‐TFAMoplex dose escalation using PLC‐Cn‐NB_CD9_‐Cn (0.25–25 nM), TFAM‐VRK1‐Cc (1.6–160 nM) and indicated DNA concentrations on HeLa cells. The protein to DNA ratio was fixed for dose escalation studies. g) Dose escalation curves showing fits as dotted lines for CRT‐TFAMoplex (orange) and CRT2‐TFAMoplex (green) specifying EC_50_ values. For all experiments, cells were transfected with pDNA encoding for GFP in 99% FBS for 30 min and transfection efficiency was assessed the following day by flow cytometry quantifying GFP+ cells. P, PLC; LP3000, Lipofectamine 3000; NTC, non‐treated control. Dose escalation curves were fitted by non‐linear regression constraining bottom and top values to 0% and 100%, respectively. Data shown as mean ± SD of N = 3–4 independent experiments, each performed in technical triplicates. Data was analyzed using one‐way ANOVA with Tukey's multiple comparison test. Statistical significance is specified with *p < 0.05, **p < 0.01, ***p < 0.001, ****p < 0.0001.

HeLa cells were transfected with the different systems using pDNA encoding for GFP as reporter gene. First, transfection experiments with CRT‐TFAMoplexes and increasing amounts of PLC‐Cn‐NB_CD9_‐Cn were conducted to determine the optimal concentration of the latter. Flow cytometry data indicated that both 2 and 5 nM PLC‐Cn‐NB_CD9_‐Cn resulted in maximal transfection, with no significant difference between these two concentrations (Figure S11, Supporting Information). Therefore, we selected 2 nM for the subsequent experiments. Transfection efficiency of the CRT‐TFAMoplexes on HeLa cells was 1.5‐fold higher compared to the original TFAMoplex (containing 16 nM PLC‐TFAM) as well as to TFAMoplexes containing 2 nM PLC‐NB_CD9_‐TFAM, where PLC is directed to the membrane but not released (**Figure** **6**a). Even though the CRT‐TFAMoplexes displayed increased transfection efficiency compared to the original TFAMoplexes, the detected endosomal disruption of both systems was comparable (Figure 5a,b). This indicates the sensitivity limit of the Gal8‐mRuby3 assay. Moreover, the CRT TFAMoplexes exhibited a fourfold higher transfection efficiency compared to Lipofectamine 3000 (LP3000), a state‐of‐the‐art transfection agent (Figure 6a). When the original TFAMoplexes were formed with the same amount of PLC (2 nM PLC‐TFAM), the CRT‐TFAMoplexes exhibited a threefold higher transfection efficiency. The resulting mean GFP intensities showed no significant differences among these systems (Figure S12, Supporting Information) indicating that similar amounts of DNA reached the nucleus. CRT‐TFAMoplexes with TFAM‐Cc substituted by TFAM, that is, without coupling of PLC‐Cn‐NB_CD9_‐Cn, resulted in no transfection (Figure 6a). This indicates that PLC must be bound to the complexes for successful transfection. Additionally, no transfection was detected when using the control PLC‐Cn‐NB_ALFA_‐Cn instead of PLC‐Cn‐NB_CD9_‐Cn, indicating the requirement of directing PLC to the membrane at such low enzyme concentrations. Remarkably, transfecting CD9 KO HeLa cells with the same systems resulted in drastic differences compared to HeLa cells (Figure 6b). There, the original TFAMoplexes performed best (twofold increase in transfection efficiency compared to CRT‐TFAMoplexes), while the other systems resulted in very little or no transfection. Note that CD9 KO HeLa cells were transfected with 2.5 times higher concentrations compared to HeLa cells, since the transfection efficiency using the CD9 KO cell line was generally lower. When using equal concentrations, transfection efficiency exhibited the same trend compared to using 2.5 times higher concentrations (Figure S13, Supporting Information). Cell viability assay indicated only little cytotoxicity when increasing concentrations of CRT‐TFAMoplexes were applied on HeLa cells (Figure S14, Supporting Information).

Next, we transfected HeLa cells with CRT2‐TFAMoplexes (PLC‐Cn‐NB_CD9_‐Cn, TFAM‐VRK1‐Cc, pDNA) titrating TFAM‐VRK1‐Cc to determine its optimal concentration. Transfection data showed that 24 nM as well as 32 nM TFAM‐VRK1‐Cc resulted in maximal transfection efficiency (Figure 6c), and 32 nM were selected for subsequent experiments to keep the total TFAM concentration equal to the other systems. Then, we titrated PLC‐Cn‐NB_CD9_‐Cn in combination with the CRT2‐TFAMoplexes to assess its optimal concentration. Maximal transfection efficiency was achieved with 5 nM PLC‐Cn‐NB_CD9_‐Cn (Figure 6d). Strikingly, under these conditions the transfection efficiency (≈50% positive cells) was increased 1.5‐fold compared to the CRT‐TFAMoplexes and 2.5‐fold compared to the original TFAMoplexes. Moreover, the resulting mean GFP intensity was significantly enhanced by 1.7‐fold compared to the original TFAMoplex system (Figure S15, Supporting Information). This indicates that increased amounts of DNA reached the nucleus due to enhanced endosomal escape. Dose escalation studies with the CRT‐TFAMoplexes (Figure 6e) and the CRT2‐TFAMoplexes (Figure 6f) confirmed that the latter system performed best in transfecting HeLa cells, with approximately threefold lower half maximal effective concentration (EC_50_) for the CRT2‐TFAMoplexes (EC_50_ of 164 ng mL^−1^ DNA) compared to the CRT‐TFAMoplexes (EC_50_ of 456 ng mL^−1^ DNA) (Figure 6g).

**Figure 7:**
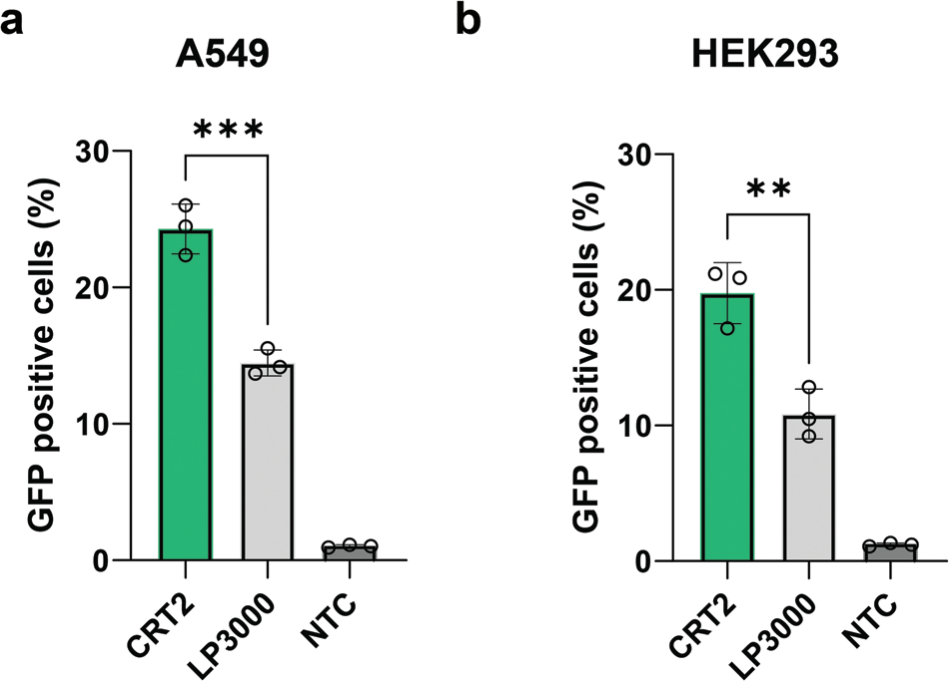
CRT2‐TFAMoplexes efficiently transfect A549 and HEK293 cells in serum. a) Transfection of A549 cells using CRT2‐TFAMoplexes (10 nM PLC‐Cn‐NB_CD9_‐Cn, 64 nM TFAM‐VRK1‐Cc) or LP3000 with 400 ng mL^−1^ pDNA. b) Transfection of HEK293 cells using CRT2‐TFAMoplexes (5 nM PLC‐Cn‐NB_CD9_‐Cn, 32 nM TFAM‐VRK1‐Cc) or LP3000 with 200 ng mL^−1^ pDNA. Cells were transfected with pDNA encoding for GFP in 99% FBS for 30 min and transfection efficiency was assessed the following day by flow cytometry quantifying GFP^+^ cells. LP3000, Lipofectamine 3000; NTC, non‐treated control. Data shown as mean ± SD of N = 3 independent experiments, each performed in technical triplicates. Data was analyzed using one‐way ANOVA with Tukey's multiple comparison test. Statistical significance is specified with **p < 0.01, ***p < 0.001.

To assess the generalizability of the CRT2‐TFAMoplexes in other cell lines, we performed additional transfection studies using the lung carcinoma A549 and human embryonic kidney (HEK) 293 cells (**Figures** **7** and S16, Supporting Information). Both cell lines were transfected in full serum for 30 min. LP3000 was used as positive control and applied at the same conditions. The CRT2‐TFAMoplexes outperformed LP3000 in both cell lines exhibiting an increased transfection efficiency (GFP positive cells) of ≈1.5‐ and ≈2‐fold in A549 and HEK293 cells, respectively (Figure 7).

In summary, these results demonstrated that integrating the capture, release and targeting of PLC to TFAMoplexes greatly enhances endosomal escape and transfection efficiency in various cell lines without altering much the physiochemical properties of the complexes.

## 3 Discussion

Non‐viral gene delivery vectors are usually associated with inefficient endosomal escape, which impairs their effectiveness.^[^ ^13^, ^14^, ^15^ ^]^ To create potent gene delivery systems, strategies for efficient endosomal escape must be devised. Here, we describe a modular calcium‐responsive capture, release and targeting system for PLC enabling potent endosomal escape and efficient transfection in combination with the TFAMoplex DNA delivery agent.

Capture and release were achieved by employing human split Cal9k. We demonstrated that the two split parts, Cn and Cc, can be exploited to link passenger proteins at extracellular calcium concentrations and quickly releasing them at conditions present in the endosomal compartment, also when applied to TFAMoplexes. A similar calcium‐sensitive system was developed previously in combination with cell‐penetrating peptide (CPP)‐mediated cargo delivery.^[^ ^55^, ^56^ ^]^ In this system, the cargo was fused to a calmodulin binding‐sequence (CBS‐cargo) and coupled to calmodulin fused to the CPP TAT (TAT‐CaM). Non‐covalent CaM‐CBS linkage displayed similar calcium‐dependent properties as split Cal9k, that is, association at high and dissociation at low calcium concentrations. However, an advantage of the split Cal9k system over the CaM‐CBS system is that Cn (≈5 kDa) and Cc (≈4 kDa) are both small peptides compared to CaM (≈17 kDa). Hence, employing the split Cal9k domains is not expected to greatly alter the properties of passenger proteins or delivery vectors.

By fusing NB_CD9_ to PLC, we could substantially increase its potency in inducing endosomal rupture on HeLa cells. Notably, eight times more PLC was required to obtain comparable endosomal disruption when using the non‐targeted PLC‐TFAM. By using PLC‐Cn‐NB_ALFA_‐Cn as negative control, as well as CD9 KO HeLa cells, we showed that the increased potency resulted from specifically directing PLC via CD9 to the membrane, and not from structural differences inherent to the fusion constructs altering, for example, interaction of PLC with the membrane.

Integration of the capture, release and targeting system to TFAMoplexes increased the transfection efficiency. Using the control construct PLC‐NB_CD9_‐TFAM, which enables membrane recruitment of covalently‐bound PLC, at 2 nM resulted in increased transfection efficiency compared to PLC‐TFAM. Notably, the combination with CD9 targeting further boosted transfection efficiency, suggesting that PLC might not efficiently reach the membrane in the original TFAMoplexes, as supported by previous work conducted in our laboratory.^[^ ^36^ ^]^ However, potential intracellular downstream effects when targeting CD9 should be considered, since CD9 was shown to interact with other proteins resulting in the formation of tetraspanin‐enriched microdomains (TEMs) responsible for mediating different cellular functions.^[^ ^57^ ^]^

Further optimization resulted in the CRT2‐TFAMoplexes leading to a threefold increased transfection efficiency and 1.7‐fold increased mean GFP intensity while requiring 3.2 times less PLC compared to the original TFAMoplexes (Figure 6 and Figure S15, Supporting Information), demonstrating enhanced endosomal escape. To the best of our knowledge, only engineered perfringolysin O (PFO) enabled efficient non‐viral delivery of silencing RNA (siRNA) at similar concentrations (0.5–5 nM).^[^ ^58^ ^]^ However, PFO was added separately, that is, not coupled, with the siRNA carrier to cells. The resulting EC_50_ of the CRT2‐TFAMoplexes was 164 ng mL^−1^ DNA corresponding to ≈48 pM DNA or ≈62 000 plasmids cell^−1^, which is orders of magnitude lower compared to other non‐viral nucleic acid delivery systems.^[^ ^14^ ^]^ Interestingly, with the CRT2‐TFAMoplexes, the concentration of PLC‐Cn‐NB_CD9_‐Cn required to achieve maximal transfection plateaued at 5 nM (Figure 6d), which could suggest that beyond this concentration, endosomal escape is not the limiting step for transfection anymore. There was no indication that cytotoxicity caused the decrease in transfection efficiency at the tested PLC concentrations (Figure S14, Supporting Information).

Colocalization of TFAMoplexes containing fluorescently‐labelled DNA and endosomal permeation provided evidence that the complexes enter the cytoplasm of cells via endosomal escape rather than via other routes, such as by directly hydrolyzing the cell surface membrane (Figure 5). This aligns with the fact that PLC exhibits low activity at neutral pH and highest activity at acidic pH, as encountered in the endosomal compartment.^[^ ^23^ ^]^

A substantial advantage of our system is its potential adaptability to various delivery agents by simply incorporating the small Cc moiety to the desired vector. For protein‐based systems, Cc can be genetically fused, while for other delivery agents bioconjugation techniques, such as maleimide chemistry,^[^ ^59^ ^]^ could be applied. PLC‐Cn‐NB_CD9_‐Cn can then be captured to the system in FBS or buffers containing low mM calcium concentrations with the aim to enable efficient endosomal escape. Inside the cytoplasm, the PLC construct is not bound to the delivery vector anymore, thus not affecting the vector's intracellular mechanism of action.

## 4 Conclusion

In conclusion, we developed a modular system to enhance PLC‐mediated endosomal escape enabling efficient DNA delivery into cells. Importantly, this calcium‐responsive and CD9‐targeted system might be translated to other delivery agents currently restricted due to poor endosomal escape. Therefore, our system expands the toolbox for the cytoplasmic delivery of macromolecular drugs.

## 5 Experimental Section

### Chemicals and Consumables

Dithiothreitol (DTT), Halt protease‐inhibitor‐cocktail, polyethyleneimine (PEI), imidazole, heparin agarose, KCl, Tris‐base, sodium dodecyl sulfate (SDS), 2‐mercaptoethanol, 4‐(2‐hydroxyethyl)‐1‐piperazineethanesulfonic acid (HEPES), agarose, kanamycin sulfate, glucose, Hoechst 33342, and Amicon Ultra‐15 PLGC Ultracel‐PL Membrane were purchased from Sigma‐Aldrich (St. Louis, MO, USA). Phosphate‐buffered saline (PBS) (1 mM KH_2_PO_4_, 155 mM NaCl, 3 mM Na_2_HPO_4_), FBS, Dulbecco's Modified Eagle Medium (DMEM) GlutaMAX, penicillin‐streptomycin (pen‐strep), LP3000, CellMask Deep Red, NaCl, glycerol, bromphenol blue, ethylenediaminetetraacetic acid (EDTA), ZnCl_2_, FastDigest Buffer, FastDigest restriction enzymes and trypsin‐EDTA were obtained from Thermo Fisher Scientific (Waltham, MA, USA). Coomassie Brilliant Blue G‐250, CaCl_2_, paraformaldehyde (PFA) and bovine serum albumin (BSA) were purchased from Fluka‐Chemie AG (Buchs, Switzerland). Chloramphenicol and MgCl_2_ were obtained from abcr GMBH (Karlsruhe, Germany). Isopropyl‐β‐D‐thiogalactopyranoside (IPTG), 2‐(*N*‐morpholino)ethanesulfonic acid (MES), lysozyme, Tris‐acetate‐EDTA (TAE) buffer (50×) (2 M Tris, 1 M acetic acid, 0.05 M EDTA) were purchased from AppliChem (Darmstadt, Germany). Strep‐TactinXT 4Flow high capacity resin, Buffer W and Buffer XT were purchased from IBA Lifesciences (Göttingen, Germany). Ni‐nitrilotriacetic acid (NTA) Superflow was obtained from Qiagen (Venlo, Netherlands). Furimazine was purchased from TargetMol (Boston, MA, USA). GelRed DNA dye was purchased from Biotium (Hayward, CA, USA). Lysogeny Broth (LB) was obtained from Lab Logistics Group (Meckenheim, Germany).

### Plasmid Construction

All constructs were created by FastDigest restriction enzyme‐based molecular cloning. In general, cloning and PCR experiments were performed according to the manufacturer's protocol. Synthesized gene fragments were purchased from Twist Bioscience (South San Francisco, CA, USA) or from GeneArt (Thermo Fisher Scientific), as indicated in Table S1, Supporting Information.

Note that only the mutant TFAM^A105C, V109C^ was used in this study, which is for simplicity referred to as TFAM. The plasmids pET_MBP‐PLC‐TFAM, pET_TFAM‐VRK1, pET_TFAM, pET_TFAM‐sh2 were produced as previously described by this group.^[^ ^36^ ^]^ The pET vectors are based on pET His6 TEV LIC cloning vector (1B) from Scott Gradia (Addgene plasmid # 29 653, Addgene, Cambridge, MA, USA).

All constructs were cloned into the pET vector containing a N‐terminal His_6_‐tag for purification via immobilized metal affinity chromatography (IMAC). The constructs Gamillus‐NB_CD9_, NB_CD9_, MBP‐PLC‐Cn‐NB_CD9_‐Cn and MBP‐PLC‐Cn‐NB_ALFA_‐Cn additionally contain a C‐terminal Strep‐tag II before the stop codon. Note that constructs with MBP contain a Tobacco Etch Virus (TEV) protease cleavage site after the MBP domain. The applied cloning strategy specifying backbones, inserts and restriction enzymes used can be found in Table S1, Supporting Information. The sequences of all constructs were confirmed by sequencing (Microsynth AG, Balgach, Switzerland) and can be found in Table S1, Supporting Information.

### Protein Expression

Constructs containing a nanobody moiety were transformed and expressed in chemically competent *E. Coli* Shuffle T7 cells (New England Biolabs, Ipswich, MA, USA). MBP‐SmBiT‐Cn, MBP‐Cc‐LgBiT, TFAM‐VRK1 and TFAM‐VRK1‐Cc were transformed into chemically competent *E. coli* One Shot BL21 Star(DE3) cells (Thermo Fisher Scientific). All other constructs were transformed into chemically competent *E. coli* BL21(DE3) pLysS cells (Promega AG, Dübendorf, Switzerland).

Cells harboring plasmids encoding for the different constructs were grown in LB medium supplemented with 0.2% (w/v) glucose and 50 µg mL^−1^ kanamycin sulfate. For plasmids in *E. coli* BL21(DE3) pLysS cells, 30 µg mL^−1^ chloramphenicol were additionally added to the medium. Cells were grown at 37 °C with 210 rpm shaking (Multitron shaker, INFORS HT, Bottmingen, Switzerland) until reaching an optical density measured at 600 nm (OD600) of 0.4–0.6. Protein expression was induced with 0.4 mM IPTG. Cells were then incubated at 20 °C, 210 rpm shaking overnight, except for cells harboring pET_TFAM‐Cc, pET_TFAM‐LgBiT, pET_MBP‐PLC‐TFAM, pET_TFAM, pET_TFAM‐VRK1 and pET_TFAM‐VRK1‐Cc, which were incubated at 30 °C, 210 rpm for 4–6 h. Subsequently, cells were harvested by centrifugation at 4000 x *g*, 4 °C for 10 min (Heraeus Megafuge 16R centrifuge, Thermo Scientific, Waltham, MA, USA) and cell pellets were frozen at −20 °C.

### Protein Purification

For purification of all constructs, cell lysis and IMAC were performed with the same protocol. Note that 1 mM DTT was included for purification of all proteins except for constructs containing NB_CD9_ or NB_ALFA_, which contain disulfide bridges. Samples were always handled on ice, except during cell lysis. Affinity chromatography was always performed at room temperature (RT).

Cell pellets containing expressed proteins were thawed on ice and resuspended in Lysis Buffer (20 mM HEPES pH 7.4, 500 mM NaCl, 10% (v/v) glycerol, 1 mg mL^−1^ lysozyme, 1× protease inhibitor cocktail, ± 1 mM DTT). The suspension was incubated at RT for 30 min followed by sonication on ice for 3 × 1 min, 5 s pulses, 10 s breaks, amplitude 50 (FB705 sonicator, Thermo Fisher Scientific). Lysates were adjusted to a final concentration of 1 M NaCl and 0.1% (w/v) PEI was added to precipitate genomic DNA. Samples were then clarified by centrifugation at 30 000 × *g*, 4 °C for 1 h (Heraeus Megafuge 16R centrifuge, Thermo Fisher Scientific) and supernatants were collected, filtered through a 0.2‐µm filter and 10 mM imidazole were added. Samples were then loaded on Ni‐NTA agarose resin pre‐equilibrated with IMAC buffer (20 mM HEPES pH 7.4, 500 mM NaCl, 10% (v/v) glycerol, 10 mM imidazole, ± 1 mM DTT). Flowthrough was re‐loaded and the resin was subsequently washed with 25 column volumes (CV) IMAC buffer containing 25 mM imidazole. Subsequently, the protein was eluted with IMAC buffer containing 250 mM imidazole and protein concentration was estimated by spectrophotometry at 280 nm (NanoPhotometer Pearl, Implen GmbH, Munich, Germany) using the respective molecular weights and extinction coefficients (Table S1, Supporting Information) that were determined with the ProtParam online tool (ExPASy, Bioinformatics Resource Portal, Switzerland).

The constructs MBP‐SmBiT‐Cn, MBP‐Cc‐LgBiT and MBP‐SmBiT‐Cn‐NB_CD9_‐Cn were purified with a single purification step. Following IMAC purification the samples were buffer exchanged into storage buffer (20 mM HEPES pH 7.4, 500 mM NaCl, 10% (v/v) glycerol, 10 mM imidazole, ± 1 mM DTT) by ultracentrifugation at 4000 x *g*, 4 °C (Sorvall ST 16R centrifuge, Thermo Fisher Scientific) using Amicon ultra centrifugal filters with a molecular weight cut‐off (MWCO) at least two times smaller than the molecular weight of the corresponding protein.

The constructs Gamillus‐NB_CD9_ and NB_CD9_ were additionally purified via strep‐tag affinity chromatography. IMAC eluates were loaded on Strep‐TactinXT 4Flow high capacity resin pre‐equilibrated with Buffer W (100 mM Tris‐HCl pH 8, 150 mM NaCl, 1 mM EDTA). The resin was then washed with 8 CV Buffer W followed by elution with Buffer BXT (100 mM Tris‐HCl pH 8, 150 mM NaCl, 1 mM EDTA, 50 mM biotin). Eventually, buffer exchange was performed as described above into Storage buffer without DTT.

The constructs TFAM‐LgBiT, TFAM, TFAM‐Cc, TFAM‐VRK1, TFAM‐VRK1‐Cc and TFAM‐Gamillus were additionally purified via heparin affinity chromatography. Therefore, IMAC eluates were diluted with ice cold double‐distilled water (ddH_2_O) to reach a final salt concentration of 180 mM and immediately loaded on heparin agarose resin pre‐equilibrated with heparin buffer (PBS, 1 mM DTT). Flowthrough was passed through the column a second time. Then the resin was washed with 12 CV heparin buffer. Subsequently, elution was performed with heparin buffer supplemented with 1 M KCl. Eventually, a buffer exchange was performed as described above into Storage buffer.

For further purification of MBP‐PLC‐TFAM and MBP‐PLC‐NB_CD9_‐TFAM, TEV protease^[^ ^60^ ^]^ (1:10 (w/w)) was added to the IMAC eluates to cleave off MBP. Note that this step is crucial since PLC is only active with its wildtype free N‐terminus, which starts with a tryptophane (W). MBP is connected via the TEV cleavage site ENLYFQ/W (single amino‐acid letter code, forward slash specifying position of cleavage), which is not efficiently cleaved by TEV. That is why comparably large amounts of TEV were added to achieve complete cleavage. The cleavage reactions were incubated overnight at 4 °C. The next day, heparin affinity chromatography was performed as described above to remove free MBP and TEV, with the exception that no DTT was used for PLC‐NB_CD9_‐TFAM. Eventually, the samples containing PLC‐TFAM or PLC‐NB_CD9_‐TFAM were buffer exchanged as described above with the exception that a different storage buffer was used (50% (v/v) PBS, 20% (v/v) glycerol, 5 mM MgCl_2_) and 1 mM DTT was only added to the buffer for PLC‐TFAM.

For further purification of MBP‐PLC‐Cn‐NB_CD9_‐Cn and MBP‐PLC‐Cn‐NB_ALFA_‐Cn, TEV‐mediated cleavage was performed as described above. The samples were then purified via strep‐tag affinity chromatography as described above to remove free MBP and TEV. Buffer exchange for the samples containing PLC‐Cn‐NB_CD9_‐Cn or PLC‐Cn‐NB_ALFA_‐Cn was performed as described above using the same storage buffer as for PLC‐TFAM but without DTT.

For all purified constructs, final protein concentrations were estimated by spectrophotometry at 280 nm. Protein purity was assessed by SDS polyacrylamide gel electrophoresis (SDS‐PAGE) analysis.

### SDS‐PAGE Analysis

SDS‐PAGE images of all constructs can be found in Figure S17, Supporting Information. Protein samples were prepared by adding 1× Laemmli buffer (37 mM Tris‐HCl pH 6.8, 6% (w/v) SDS, 4.8% (v/v) glycerol, 9% (v/v) 2‐mercaptoethanol, 0.03% (w/v) bromphenol blue) and incubated at 95 °C for 1 min. Subsequently, 1–2 µg protein was loaded per well of a Mini‐PROTEAN TGX Stain‐Free gel (4–20%, Bio‐Rad, Hercules, CA, USA) and electrophoresis was performed in a Mini‐PROTEAN 2‐D electrophoresis cell (Bio‐Rad) at 100 V for 1.5 h. The gel was stained with Coomassie Brilliant Blue G‐250 and imaged with a ChemiDoc MP Gel reader (Bio‐Rad). Protein purity was evaluated using densiometric analysis with the software ImageJ.^[^ ^61^ ^]^

### Luminescence Assays

All luminescence assays were performed in white 384‐well flat bottom plates (Greiner Bio‐One, Kremsmünster, Austria) with furimazine as luciferase substrate. Luminescence signal was recorded with a Spark Multimode Microplate reader (Tecan, Männerdorf, Switzerland) using an integration time of 1000 ms without attenuation and without filters.

Association at pH 7.4 with different CaCl_2_ concentrations (0–2 mM) was measured in 75 mM HEPES pH 7.4, 100 mM NaCl using 2 nM equimolar MBP‐Cc‐LgBiT and MBP‐SmBiT‐Cn and 1 µM furimazine in a final volume of 75 µL. Association in FBS with MBP‐SmBiT‐Cn was measured in 80% (v/v) FBS ± 6 mM EDTA using 10 nM MBP‐Cc‐LgBiT ± 10 nM MBP‐SmBiT‐Cn and 1 µM furimazine in a final volume of 100 µL. Association in FBS with MBP‐SmBiT‐Cn‐NB_CD9_‐Cn was measured as described above with the exception that 10 nM MBP‐LgBiT‐Cn and 40 nM MBP‐SmBiT‐Cn‐NB_CD9_‐Cn or 40 nM MBP‐SmBiT‐Cn (as control) were used.

Dissociation experiments at pH 7.4 and 6.0 were conducted by first incubating 100 nM equimolar MBP‐Cc‐LgBiT and MBP‐SmBiT‐Cn in buffer A (20 mM HEPES pH 7.4, 150 mM NaCl, 2 mM CaCl_2_) or in buffer B (20 mM MES pH 6.0, 150 mM NaCl, 2 mM CaCl_2_) for 30 min to ensure association. Then, the sample was diluted 500‐fold into buffer A or into buffer B containing different CaCl_2_ concentrations (0–2 mM) or 2 mM EDTA, resulting in a final equimolar protein concentration of 0.2 nM. Luminescence was immediately recorded using 1 µM furimazine in a final volume of 100 µL. Dissociation for MBP‐SmBiT‐Cn‐NB_CD9_‐Cn was measured as described above, with the exception that 100 nM MBP‐Cc‐LgBiT and 400 nM MBP‐SmBiT‐Cn‐NB_CD9_‐Cn were used in the incubation step, resulting in final protein concentrations of 0.2 nM MBP‐Cc‐LgBiT and 0.8 nM MBP‐SmBiT‐Cn‐NB_CD9_‐Cn after dilution.

Association to TFAMoplexes was measured by forming complexes with 900 nM TFAM‐LgBiT, 700 nM TFAM‐Cc, 10 ng µL^−1^ pDNA encoding for enhanced GFP (pEGFP) (Addgene, pc3DNA, Table S1, Supporting Information) and adding MBP‐SmBiT‐Cn (350 nM) or MBP‐SmBiT‐Cn‐NB_CD9_‐Cn (350 nM) in final 80% (v/v) FBS (Thermo Fisher Scientific) with 1 µM furimazine in a final volume of 50 µL. As controls, different components were omitted and 6 mM EDTA was added.

### Cell Culture

HeLa (ATCC CCL‐2) and A549 (ATCC CCL‐185) cells were purchased from ATCC (Manassas, VA, USA). HEK293 cells were a kind gift from Dr. David Vukovic (Department of Biochemistry, University of Zurich, Zürich, Switzerland). CD9 KO HeLa cells were a kind gift from Dr. Eric Rubinstein (Center of Immunology and Microbial Infections, Sorbonne University, Paris, France). HeLa‐Gal8‐mRuby3 cells were kindly provided by Dr. Simone Berger (Department of Pharmacy, LMU Munich, Munich, Germany). All cell lines (passage number 3–30, tested negative for mycoplasma contamination (MycoAlert Kit, Lonza AG, Basel, Switzerland)) were maintained in complete growth medium (DMEM GlutaMAX, 10% (v/v) FBS, 1% (v/v) pen‐strep) at standard cell culture conditions (37 °C, 5% CO_2_, humidified atmosphere).

### Confocal Laser Microscopy

For confocal microscopy experiments, 30 000 HeLa cells were seeded per 1 cm^2^ microscopy slide well (μ‐slide 8 well chambered coverslips with glass bottom, ibidi, Martinsried, Germany) with a working volume of 250 µL. 1 day after seeding, the cells were washed once with PBS and fresh complete growth medium or 100% FBS was added to the cells. After treatment, the cells were fixed with 4% (w/v) methanol‐free PFA for 20 min and stained with 2.5 µg mL^−1^ Hoechst 33342 and optionally 1.25 µg mL^−1^ CellMask Deep Red for 15 min. After washing three times with PBS, 250 µL PBS was added to each well. Microscopy samples were stored at 4 °C and imaged within 1 week.

Confocal laser microscopy was performed on an Eclipse Ti2 inverse spinning disk confocal microscope (Nikon, Tokyo, Japan) using an 100 × 1.45 CFI Plan Apo Oil objective (Nikon) with immersion oil type F (Nikon) and a sCMOS Orca Fusion BT camera (Hamamatsu Photonics, Hamamatsu City, Japan). The Hoechst channel was excited at 405 nm and fluorescent emission was collected with a 447 nm filter (bandwidth 60 nm). Green fluorophores were excited at 488 nm and detected using a 525 nm filter (bandwidth 50 nm). mRuby3 was excited at 561 nm and fluorescence was detected with a 600 nm filter (bandwidth 52 nm). 35 z‐stacks were acquired with a slice thickness of 0.3 µm. The software ImageJ^[^ ^61^ ^]^ was used for image processing.

### NB_CD9_ Cell Association Experiments

Gamillus‐NB_CD9_ was added at 100 nM to either HeLa cells or CD9 KO HeLa cells, which were seeded 1 day before in complete growth medium as described above. For competition, 2.5 µM of unlabeled NB_CD9_ was added to the cells 10 min before addition of Gamillus‐NB_CD9_. After incubation for 30 min, the cells were washed three times with PBS. The cells were subsequently prepared and imaged as described above. Confocal microscope laser intensities were 20% with 200 ms exposure time for the 405 nm laser and 60% with 400 ms exposure time for the 488 nm laser.

### PLC Activity Assay

Phosphatidylcholine (pc)‐specific PLC activity was measured with EnzChek Direct Phospholipase C Assay kit (Thermo Fisher Scientific) according to the manufacturers protocol using 100 nM of each PLC construct in 75 mM MES pH 5.5, 150 mM NaCl, 10 µM ZnCl_2_ in 20 µL final volume in a black 384‐well flat bottom plate (Greiner Bio‐One). Fluorescence was recorded with a Spark Multimode Microplate reader (Tecan) with excitation at 490 nm and collecting emission at 520 nm.

### Colocalization with Early Endosomes

One day before transfection, HeLa cells (seeded as described above under “confocal laser microscopy”) were transduced in complete growth medium with CellLight Early Endosomes‐GFP BacMam 2.0 (Thermo Fisher Scientific) according to the manufacturer's instructions. On the day of transfection, the cells were washed three times with PBS and 240 µL 100% FBS were added to the cells. Then, CRT2‐TFAMoplexes were prepared in a 1.5‐mL test tube in ≥ 80% (v/v) FBS with 0.25 µM P‐Cn‐NB_CD9_‐Cn, 1.6 µM TFAM‐VRK1‐Cc and 10 ng µL^−1^ Cy3‐labelled pDNA (Mirusbio LCC, Madison, WI, USA) for 30 min at RT. Subsequently, 10 µL of the sample were added to the HeLa cells in 100% FBS (in a final volume of 250 µL) resulting in final concentrations of 10 nM P‐Cn‐NB_CD9_‐Cn, 64 nM TFAM‐VRK1‐Cc and 400 ng mL^−1^ Cy3‐labelled pDNA. After 1 h of incubation, the cells were washed three times with PBS and prepared and imaged as described above. Confocal microscope laser intensities were 20% with 200 ms exposure time for the 405 nm laser, 40% with 400 ms exposure time for the 488 nm laser and 20% with 200 ms exposure time for the 561 nm laser.

### Gal8‐mRuby3 Assay

For investigating endosomal permeation induced by free proteins, the different PLC constructs were applied at 15 nM, for PLC‐TFAM additionally at 120 nM, to HeLa‐Gal8‐mRuby3 cells, that were seeded 1 day before (as described above under confocal laser microscopy), and incubated for 3 h in complete growth medium. Subsequently, the cells were washed three times with PBS and prepared and imaged as described above under confocal laser microscopy. Confocal microscope laser intensities were 10% with 200 ms exposure time for the 405 nm laser and 20% with 400 ms exposure time for the 561 nm laser.

For investigating endosomal permeation induced by the TFAMoplexes and colocalization of pDNA and Galectin8‐mRuby3, pDNA encoding for enhanced blue fluorescent protein (pEBFP) (pEBFP2‐N1, Addgene, #54 595) was labelled with MFP488 using 0.1% (v/w) (Label IT Nucleic Acid Labeling Kit, Mirusbio LCC) and purified via ethanol precipitation according to the manufacturer's protocol. Formation of the different complexes was performed inside a 1.5‐mL test tube in ≥ 80% (v/v) FBS with a fixed total TFAM concentration of 1.6 µM and 10 ng µL^−1^ MFP488‐labelled pEBFP for 30 min at RT. Subsequently, 10 µL of each sample were added to HeLa‐Gal8‐mRuby3 cells prepared as described above in 240 µL 100% FBS, therefore diluting each sample 25‐fold resulting in a final total TFAM concentration of 64 nM and 400 ng mL^−1^ DNA. Final protein concentrations of each TFAMoplex can be found in Figure S7, Supporting Information. As control, LP3000 lipoplexes, prepared according to the manufacturer's protocol, were applied with the same final DNA concentration at the same conditions. After 1 h of incubation, the cells were washed three times with PBS and prepared and imaged as described above. Confocal microscope laser intensities were 20% with 200 ms exposure time for the 405 nm laser, 20% with 200 ms exposure time for the 488 nm laser and 40% with 400 ms exposure time for the 561 nm laser.

For time‐lapse live‐cell imaging of endosomal permeation induced by the CRT2‐TFAMoplexes, HeLa‐Gal8‐mRuby3 cells were prepared as described above. Before transfection, cells were washed three times with PBS and 240 µL of FluoroBrite DMEM (Thermo Fisher Scientific) supplemented with 10% (v/v) FBS and 1% (v/v) pen‐strep were added. Complexes were prepared and added to the cells as described above (final concentrations: 10 nM P‐Cn‐NB_CD9_‐Cn, 64 nM TFAM‐VRK1‐Cc and 400 ng mL^−1^ MFP488‐labelled pDNA). Time‐lapse imaging started 30 min after transfection, acquiring z‐stacks (21 steps, 0.3 µm stack size, total 5.6 µm) every 5 min. Cells were kept at 37 °C and 5% CO_2_ during imaging. Confocal microscope laser intensities were 5% with 100 ms exposure time for the 488 nm laser and 5% with 100 ms exposure time for the 561 nm laser.

### Quantification of Endosomal Permeation

To quantify Gal8‐mRuby3 foci, the ImageJ Macro 1 (Table S2, Supporting Information) was applied on z projections of maximum intensities, as well as the ImageJ Macro 2 (Table S2, Supporting Information) to quantify cells. For images of free proteins, intensity thresholding was applied from 700 to 65535 (ImageJ Macro 1) and for images of TFAMoplexes, intensity thresholding was applied from 1000 to 65535 (ImageJ Macro 3, Table S2, Supporting Information). Foci with a size from 0.02 to 200 µm^2^ and a circularity of 0.6 to 1.0 were segmented and counted. The segmented foci were related to the number of cells in one image.

### Quantification of TFAMoplex Colocalization with Endosomal Permeation

The colocalization of MFP488‐labelled DNA with intense Gal8‐mRuby3 dots was determined on z‐projections with the ImageJ Macro 4 (Table S2, Supporting Information). MFP488 signals with intensities from 1000 to 665535 with an area from 0.02 to 200 µm^2^ and a circularity of 0.6 to 1.0 were segmented. The intensities in the segmented objects were measured in the MFP488 and Gal8‐mRuby3 channel and displayed as shown in Figure 5d.

To calculate the percentage of colocalization, the average Gal8‐mRuby3 signal plus three times the SD (696.5) of the control CRT2‐TFAMoplexes without PLC was set as lower limit of Gal8‐mRuby3 signal. MFP488 objects with corresponding Gal8‐mRuby3 signal above this threshold were associated with ruptured endosomes. The percentage represents the amount of MFP488 signal in a ruptured endosome in relation to the total number of MFP488 signals.

### Nuclear Colocalization

For investigating nuclear colocalization of TFAMoplexes, HeLa cells were prepared as described above. On the day of transfection, the cells were washed three times with PBS and 240 µL fresh FBS were added to the cells. Subsequently, Gamillus‐labelled TFAMoplexes were formed inside a 1.5‐mL test tube in ≥ 80% (v/v) FBS with 0.25 µM P‐Cn‐NB_CD9_‐Cn, 0.8 µM TFAM‐VRK1‐Cc, 0.8 µM TFAM‐Gamillus and 10 ng µL^−1^ Cy3‐labelled pDNA (Mirusbio LCC) for 30 min at RT. Subsequently, 20 µL of the sample were added to the HeLa cells in 100% FBS (in a final volume of 250 µL) resulting in final concentrations of 20 nM P‐Cn‐NB_CD9_‐Cn, 64 nM TFAM‐VRK1‐Cc, 64 nM TFAM‐Gamillus and 800 ng mL^−1^ Cy3‐labelled pDNA. After 30 min, the cells were washed three times with PBS and fresh complete growth medium was added followed by incubating the cells overnight. The next day, cells were washed and imaged as described above, but with 51 z‐stacks with 0.2 µm spacing, total 10 µm. Confocal microscope laser intensities were 20% with 200 ms exposure time for the 405 nm laser, 60% with 400 ms exposure time for the 488 nm laser and 20% with 200 ms exposure time for the 561 nm laser.

### DLS Experiments

For DLS measurements, TFAMoplex formation was performed with indicated protein concentrations and 10 ng µL^−1^ pEGFP inside a micro cuvette (ZEN0040) (Malvern Panalytical, Malvern, UK) at RT for 30 min in a total volume of 80 µL using 25 mM HEPES pH 7.4, 150 mM KCl as buffer. To ensure binding of the PLC constructs containing a Cn moiety to TFAMoplexes, particle size was also measured in the same buffer containing 2 mM CaCl_2_. As control, particle size was also measured without DNA. The hydrodynamic diameter was measured based on the intensity of the scattered light using a Zetasizer pro (Malvern Panalytical). For size determination, the average peak values of 3 independent experiments were taken.

### Gel Mobility Shift Assay

An agarose gel (0.8% (w/v)) was prepared with 1× TAE buffer supplemented with GelRed DNA dye (1:20 000). The samples were prepared in 1.5‐mL test tubes with indicated protein concentrations and 10 ng µL^−1^ pEGFP in PBS with a final volume of 10 µL. As control, free DNA samples (without proteins) were included. Complex formation was performed for 30 min at RT followed by adding 2 µL FastDigest Green buffer (10×) to each sample. Subsequently, 12 µL sample corresponding to 100 ng DNA were loaded per well. Electrophoresis was performed in 1× TAE buffer at 100 mA for 1 h in an electrophoresis chamber (Sub‐Cell GT Cell, Bio‐Rad). The DNA was imaged with a ChemiDoc MP Gel reader (Bio‐Rad).

### Cell Preparation for Transfection, Particle Formation and Transfection

HeLa, CD9 KO HeLa, A549 and HEK293 cells were seeded in 24‐well tissue culture plates (TPP Techno Plastic Products AG, Trasadingen, Switzerland) to reach 90–100% confluency on the day of transfection. Right before transfection, the cells were washed three times with PBS and 500 µL 100% FBS were added to the cells.

TFAMoplex formation was performed in ≥ 80% (v/v) FBS in 1.5‐mL test tubes with a final volume of 10–100 µL. FBS was added first followed by adding the desired proteins and, eventually, pEGFP (10 ng µL^−1^). Then, the samples were mixed by tipping and incubated at RT for 30 min to allow protein‐DNA complex formation. For transfection of HeLa and HEK293 cells, 10 µL sample was added to each well of the tissue culture plate containing cells in 100% FBS, resulting in a 50‐fold dilution (200 ng mL^−1^ DNA final). For transfection of CD9 KO HeLa cells using final 500 ng mL^−1^ DNA, 25 µL of respective TFAMoplex sample were added per well, resulting in a 20‐fold dilution of complexes. For transfection of A549 cells using final 400 ng mL^−1^ DNA, 20 µL of respective TFAMoplex sample were added per well, resulting in a 25‐fold dilution of complexes. For dose escalation studies, up to 50 µL TFAMoplex were added per well (final 1000 ng mL^−1^ DNA). Note that the indicated protein and DNA concentrations correspond to the final concentrations on the cells. Cells were transfected for 30 min at standard cell culture conditions followed by washing three times with PBS and adding 500 µL fresh complete growth medium and, eventually, incubating the cells overnight.

As a control, LP3000 complexes, prepared according to the manufacturer's protocol, were applied at the same conditions as the TFAMoplex samples.

For investigating transfection efficiency after storage at 4 °C, the complexes were prepared as described above and stored at 4 °C (in the fridge) for 1 day or 1 week. HeLa cells were transfected and analyzed as described above.

Please note that all components of the TFAMoplexes (proteins and DNA) exhibit long‐term stability when stored at −80 °C (for at least a year). Once formed in serum, the complexes have to be used within hours, as storage at 4 °C for 1 day or longer significantly reduces transfection efficiency (Figure S18, Supporting Information).

### Flow Cytometry Analysis

HeLa, CD9 KO HeLa, HEK293 and A549 cells in 24‐well plates were washed three times with PBS, trypsinized for 3 min at 37 °C and transferred to a Costar round‐bottom 96‐well assay plate (Corning, New York, NY, USA) containing 20 µL FBS. Cells were pelleted at 300 x *g*, 3 min, 4 °C (Sorvall ST 16R centrifuge) followed by resuspending the pellets in ice cold FACS buffer (PBS, 2 mM EDTA, 1% (w/v) BSA) and flow cytometry analysis (CytoFLEX Flow Cytometer, Beckman Coulter Life Sciences, Nyon, Switzerland). For each sample, 10 000 cells were analyzed according to their green fluorescence (excitation at 488 nm) using the FlowJo software (Tree Star Inc., Ashland, OR, USA).

### Cell Viability Assay

HeLa cells were seeded in a 96‐well tissue culture plate (TPP Techno Plastic Products AG) at 5000 cells per well in 100 µL complete growth medium. The next day, cells were washed three times with PBS and subsequently 100 µL FBS were added to each well. TFAMoplexes were formed as described above and added to the cells at indicated concentrations followed by incubating the cells overnight. Then, CellTiter 96 Aqueous One Solution Reagent (20 µL, Promega) was given to each well. As positive control, SDS was added to a final concentration of 2% (w/v). The cells were incubated for 1.5 h at standard cell culture conditions followed by measuring absorbance at 490 nm with a Spark Multimode Microplate reader (Tecan). Data were normalized to the non‐treated control and absorbance value obtained for the positive control was subtracted from each sample.

### Statistical Analysis

Statistical analysis was conducted with the GraphPad Prism software version 10.1. In general, data are represented as mean ± SD of at least 3 independent experiments, each performed in technical triplicates. Significance was analyzed by one‐way ANOVA with Tukey's multiple comparison test.

## Conflict of Interest

The authors declare no conflict of interest.

## Author Contributions

A.K. and M.B. developed the concept of the study and designed the protein constructs. J.‐C.L. and M.B. supervised the study. A.K. designed, produced and purified protein constructs and performed luminescence, phospholipase activity, transfection, cell viability, DLS and gel mobility shift experiments. C.G. conducted the confocal microscopy experiments, scripted the ImageJ Macros and analyzed and prepared the microscopy figures. D.S. conceived the split NanoLuc system on TFAMoplexes and produced and purified TFAM‐LgBiT. A.E. helped with protein production for transfection studies. A.K. wrote the manuscript and prepared the figures. Data analysis, data evaluation, manuscript editing and proofreading were performed by A.K., C.G., J.‐C.L., and M.B. The manuscript was reviewed by all authors.

## Supporting information



Supporting Information

Supplemental Movie 1

## Data Availability

The data that support the findings of this study are available in the ETH research collection.
