# Supplementary material for: Modular Calcium‐Responsive and CD9‐Targeted Phospholipase System Enhancing Endosomal Escape for DNA Delivery
Source: Adv Sci (Weinh). 2025 Feb 25;12(15):2410815. doi: 10.1002/advs.202410815 (PMC12005733; doi:10.1002/advs.202410815)
Supplement: Supplementary file 1 — Supporting Information [file ADVS-12-2410815-s002.pdf]

ADVANCED SCIENCE
Open Access

Supporting Information

for *Adv. Sci.*, DOI 10.1002/advs.202410815

Modular Calcium-Responsive and CD9-Targeted Phospholipase System Enhancing Endosomal Escape for DNA Delivery

Alexander Klipp, Christina Greitens, David Scherer, Alexander Elsener, Jean-Christophe Leroux and Michael Burger**

Supporting information

Modular Calcium-Responsive and CD9-Targeted Phospholipase System Enhancing Endosomal Escape for DNA Delivery

Alexander Klipp,[a] Christina Greitens,[a] David Scherer,[a] Alexander Elsener,[a] Jean-Christophe Leroux,*[a] and Michael Burger*[a]

[a] A. Klipp, C. Greitens, D. Scherer, A. Elsener, Prof. Dr. J.-C. Leroux, Dr. M. Burger
Department of Chemistry and Applied Biosciences
ETH Zürich
Vladimir-Prelog-Weg 3, 8093 Zürich, Switzerland
E-mail: jleroux@ethz.ch, michael.burger@pharma.ethz.ch

Table of Contents

Figure S1……………………………………………………………………………2
Figure S2……………………………………………………………………………3-4
Figure S3……………………………………………………………………………5
Figure S4……………………………………………………………………………6-7
Figure S5……………………………………………………………………………8
Figure S6……………………………………………………………………………9
Figure S7……………………………………………………………………………10
Figure S8……………………………………………………………………………11
Figure S9……………………………………………………………………………12
Figure S10……………………………………………………………………………13
Figure S11……………………………………………………………………………14
Figure S12……………………………………………………………………………15-16
Figure S13……………………………………………………………………………17
Figure S14……………………………………………………………………………18
Figure S15……………………………………………………………………………19
Figure S16……………………………………………………………………………20
Figure S17……………………………………………………………………………21
Figure S18……………………………………………………………………………22
Supporting Movie 1 legend……………………………………………………………23
Table S1……………………………………………………………………………24-31
Table S2……………………………………………………………………………32-38

Supporting Figures

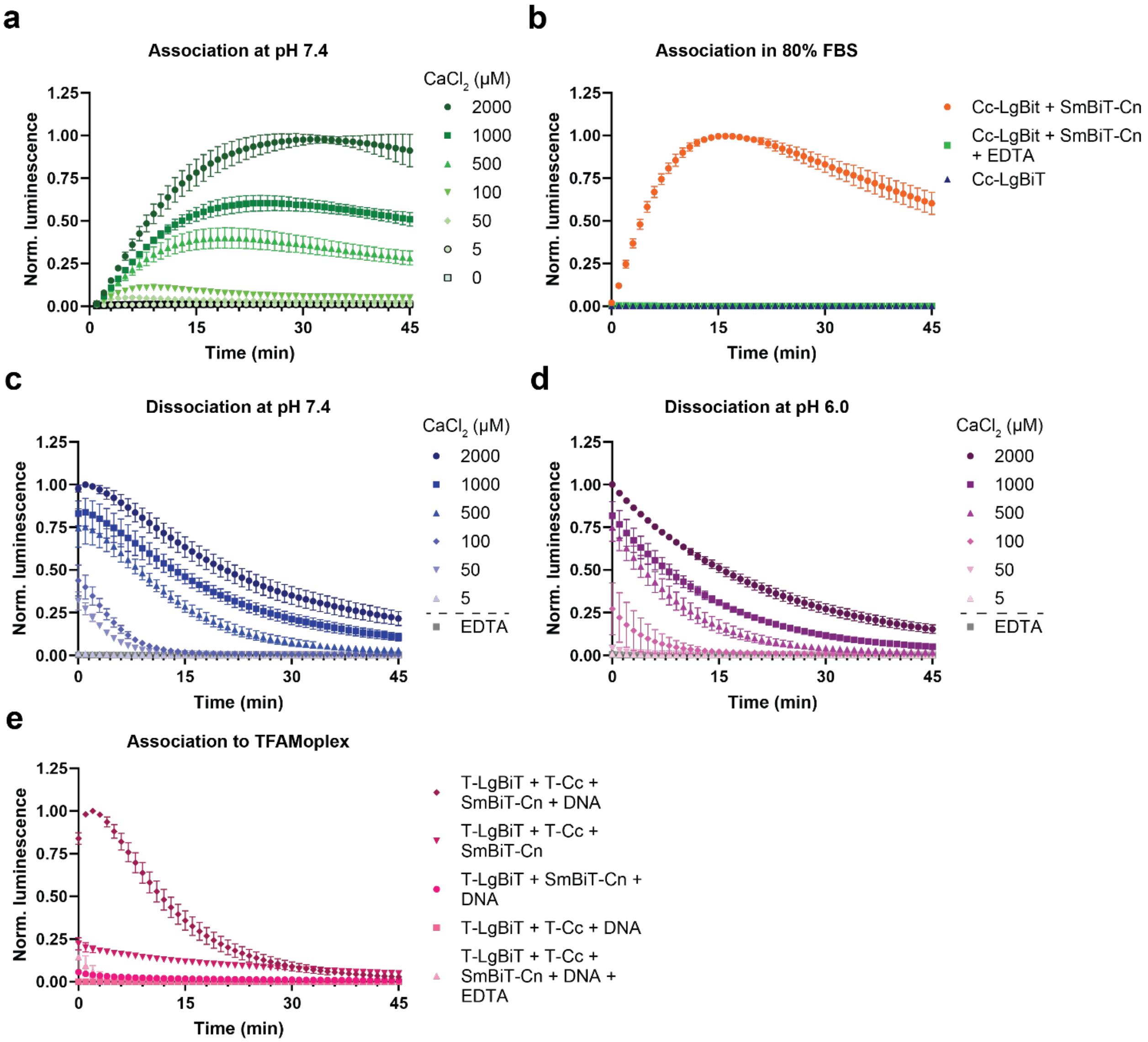

Figure S1. Split Cal9k complementation curves. (a) Split Cal9k association was determined by incubating SmBiT-Cn with Cc-LgBiT at pH 7.4 and different $CaCl_2$ concentrations with luminescence generation as read out (2 nM equimolar protein partners). (b) Split Cal9k association in 80% FBS +/- SmBiT-Cn (10 nM equimolar) and +/- 2 mM EDTA. (c) and (d) Dissociation experiments were conducted by incubating SmBiT-Cn with Cc-LgBiT at 2 mM $CaCl_2$ for 30 min ensuring binding and split NanoLuc complementation. The sample was then diluted to different $CaCl_2$ concentrations +/- 2 mM EDTA and luminescence was recorded (0.2 nM equimolar protein partners). (c) Dissociation at pH 7.4. (d) Dissociation at pH 6.0. (e) Split Cal9k association to TFAMoplexes formed in 80% FBS. After formation, SmBiT-Cn was added to the sample and luminescence was recorded. Binding curves shown for different construct combinations and +/- 6 mM EDTA (0.9 μM TFAM-LgBiT, 0.7 μM TFAM-Cc, 10 ng/μL DNA, 0.35 μM SmBiT-Cn). Data were normalized within each biological replicate. Data shown as mean ± SD of N = 3 independent experiments, each performed in technical triplicates.

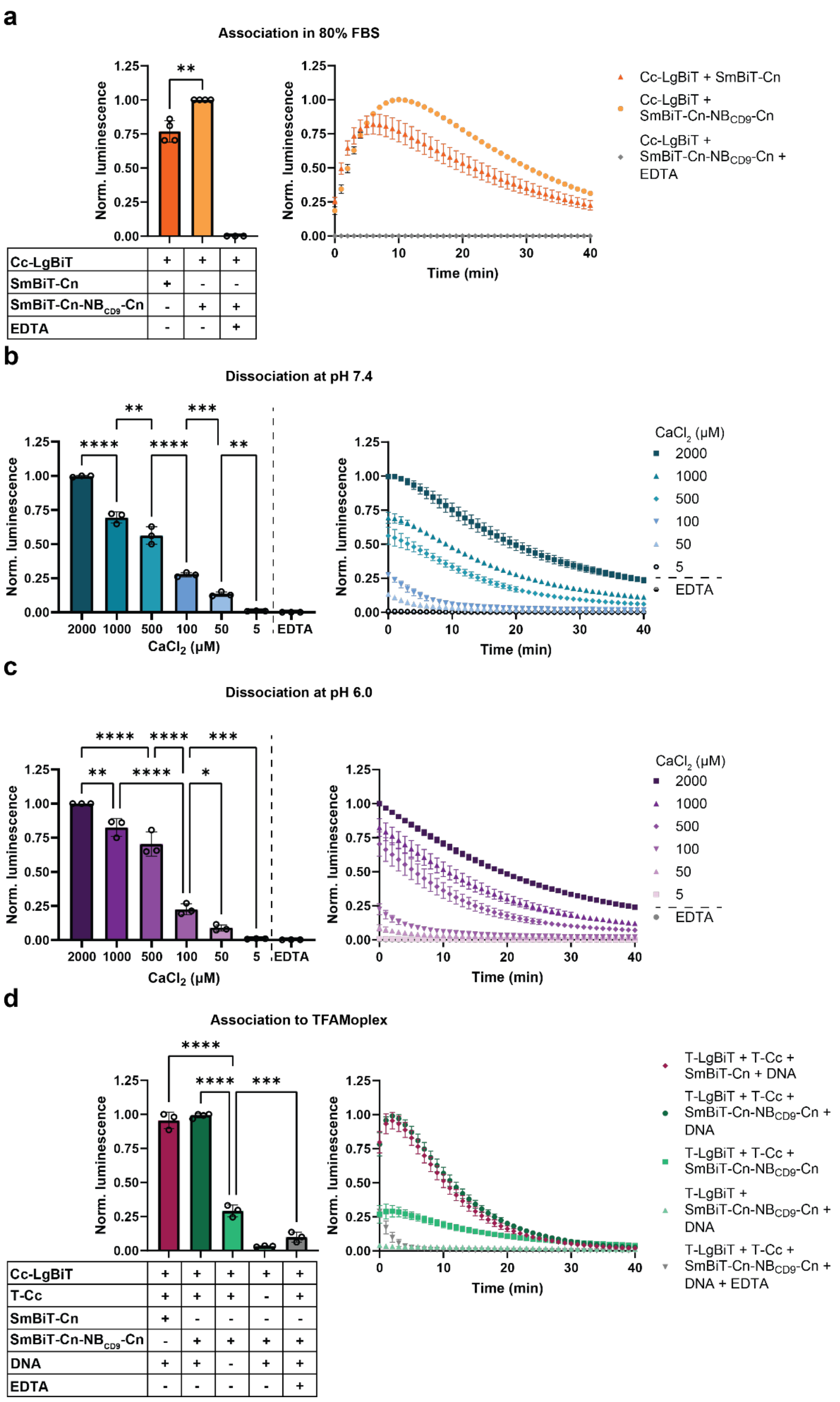

a
Association in 80% FBS
Norm. luminescence
Time (min)
Cc-LgBiT + SmBiT-Cn
Cc-LgBiT + SmBiT-Cn-NBCD9-Cn
Cc-LgBiT + SmBiT-Cn-NBCD9-Cn + EDTA
Cc-LgBiT
SmBiT-Cn
SmBiT-Cn-NBCD9-Cn
EDTA
b
Dissociation at pH 7.4
Norm. luminescence
CaCl2 (μM)
Time (min)
2000
1000
500
100
50
5
EDTA
c
Dissociation at pH 6.0
Norm. luminescence
CaCl2 (μM)
Time (min)
2000
1000
500
100
50
5
EDTA
d
Association to TFAMoplex
Norm. luminescence
Time (min)
T-LgBiT + T-Cc + SmBiT-Cn + DNA
T-LgBiT + T-Cc + SmBiT-Cn-NBCD9-Cn + DNA
T-LgBiT + T-Cc + SmBiT-Cn-NBCD9-Cn
T-LgBiT + SmBiT-Cn-NBCD9-Cn + DNA
T-LgBiT + T-Cc + SmBiT-Cn-NBCD9-Cn + DNA + EDTA
Cc-LgBiT
T-Cc
SmBiT-Cn
SmBiT-Cn-NBCD9-Cn
DNA
EDTA

Figure S2. Split Cal9k complementation using MBP-SmBiT-Cn-NB$_{CD9}$-Cn. (a) Split Cal9k association in 80% FBS using Cc-LgBiT (10 nM) +/- SmBiT-Cn (40 nM), +/- MBP-SmBiT-Cn-NB$_{CD9}$-Cn (40 nM), and +/- 6 mM EDTA. (b) and (c) Dissociation experiments were conducted by incubating MBP-SmBiT-Cn-NB$_{CD9}$-Cn with Cc-LgBiT at 2 mM $CaCl_2$ for 30 min ensuring binding and split NanoLuc complementation. The sample was then diluted to different $CaCl_2$ concentrations +/- 2 mM EDTA and luminescence was recorded. Final concentrations: 0.8 nM MBP-SmBiT-Cn-NB$_{CD9}$-Cn and 0.2 nM Cc-LgBiT. (b) Dissociation at pH 7.4. (c) Dissociation at pH 6.0. (d) Split Cal9k association to TFAMoplexes formed in 80% FBS. After formation, SmBiT-Cn or MBP-SmBiT-Cn-NB$_{CD9}$-Cn was added to the sample and luminescence was recorded. Binding curves shown for different construct combinations and +/- 6 mM EDTA (0.9 µM TFAM-LgBiT, 0.7 µM TFAM-Cc, 10 ng/µL DNA, 0.35 µM SmBiT-Cn, 0.35 µM MBP-SmBiT-Cn-NB$_{CD9}$-Cn). MBP is omitted for clarity. Bar plots (left) and corresponding complementation curves (right) are shown for each experiment. Bar plots of association experiments display values at plateau phase. Bar plots of dissociation experiments display values recorded after 1 min. Data were normalized within each biological replicate. Data shown as mean ± SD of N = 3-4 independent experiments, each performed in technical triplicates. Data was analyzed using one-way ANOVA with Tukey's multiple comparison test. Statistical significance is specified with $^*p < 0.05$, $^{**}p < 0.01$, $^{***}p < 0.001$, $^{****}p < 0.0001$.

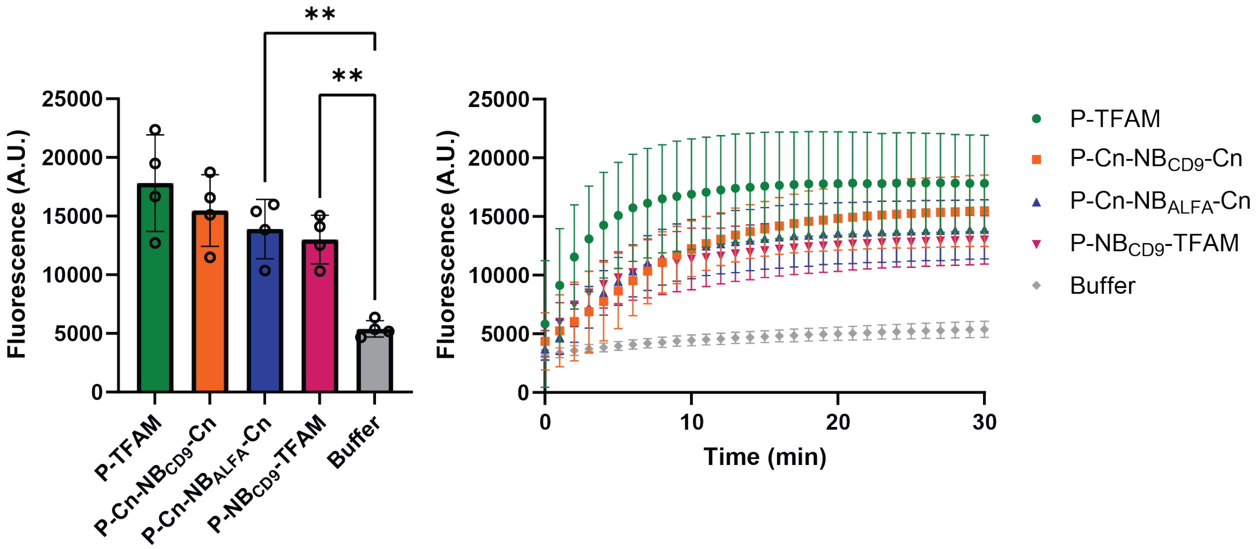

Figure S3. PLC activity of the different constructs. Activity was measured at pH 5.5 using 0.1 μM of each protein construct with EnzChek™ Direct Phospholipase C Assay kit. P, PLC. Bar plots and progression curves are shown. Bar plots display values at plateau phase. Data shown as mean ± SD of N = 4 independent experiments, each performed in technical triplicates. Data was analyzed using one-way ANOVA with Tukey's multiple comparison test. Statistical significance is specified with $^{**}p < 0.01$.

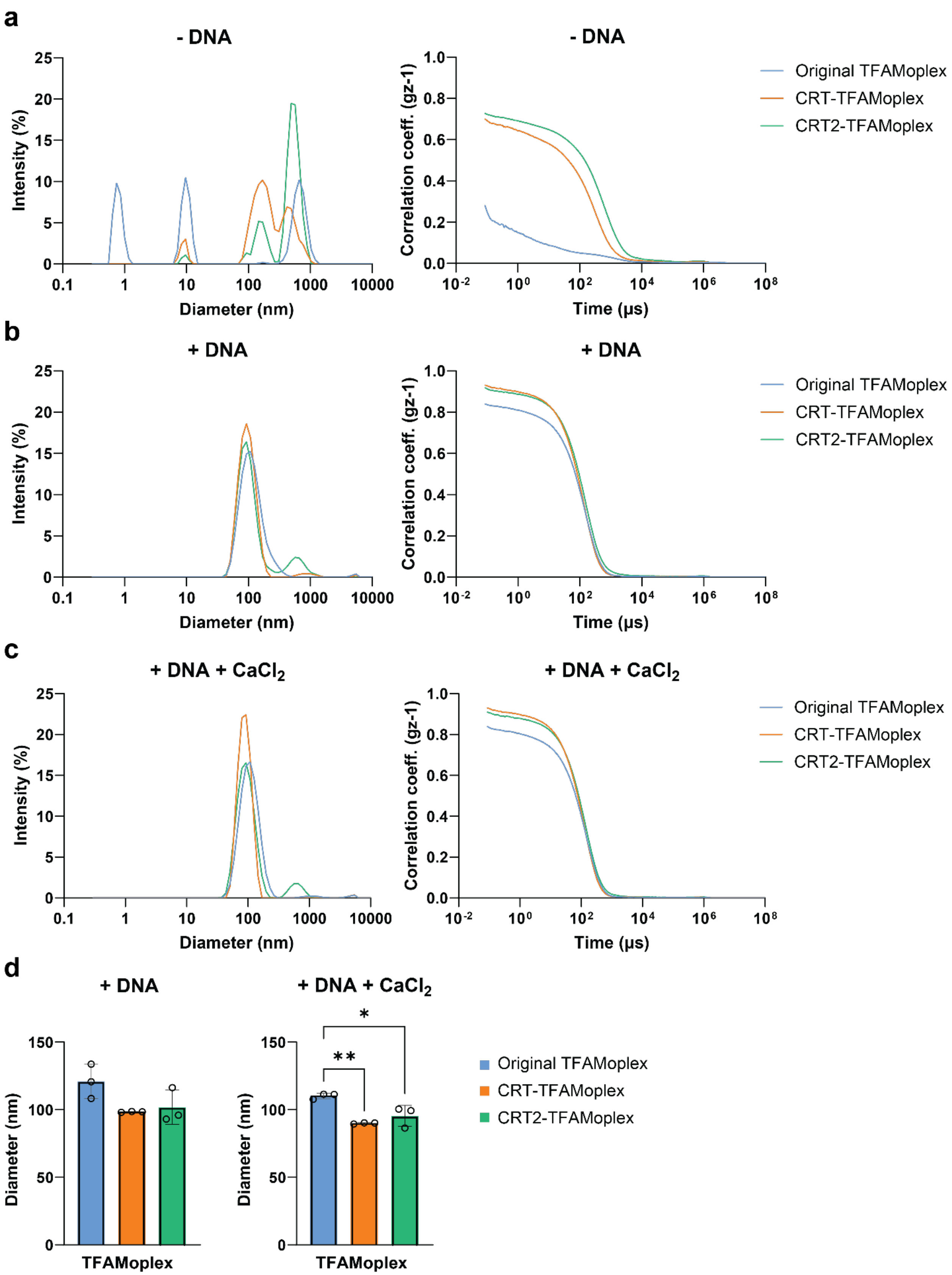
a
- DNA
Intensity (%)
Diameter (nm)
Correlation coeff. (gz-1)
Time (μs)
Original TFAMoplex
CRT-TFAMoplex
CRT2-TFAMoplex
b
+ DNA
c
+ DNA + CaCl2
d
Diameter (nm)
TFAMoplex
**
*

Figure S4. Particle size characterization of the different TFAMoplex systems by DLS. Size distribution (left) and correlogram (right) of (a) free proteins (w/o DNA), (b) indicated TFAMoplex systems with DNA, (c) indicated TFAMoplex systems with DNA and with 2 mM $CaCl_2$. Calcium is required for capturing PLC-Cn-NB_{CD9}-Cn to the complexes. (d) Bar plots displaying main peak values of the different TFAMoplex systems +/- $CaCl_2$. All experiments were performed in HEPES buffer at pH 7.4 supplemented with 150 mM KCl +/- 2 mM $CaCl_2$ and +/- DNA. For the original TFAMoplex, 250 nM total TFAM was used. For the other systems, 500 nM total TFAM was used. Original TFAMoplex (125 nM PLC-TFAM, 125 nM TFAM-VRK1, 10 ng/µL DNA), CRT-TFAMoplex (31.25 nM PLC-Cn-NB_{CD9}-Cn, 220 nM TFAM-Cc, 30 nM TFAM, 250 nM TFAM-VRK1, 10 ng/µL DNA), CRT2-TFAMoplex (80 nM PLC-Cn-NB_{CD9}-Cn, 500 nM TFAM-VRK1-Cc, 10 ng/µL DNA). N = 3 independent experiments were performed. Curves display average of one triplicate measurement. For bar plots, data shown as mean ± SD. Data was analyzed using one-way ANOVA with Tukey's multiple comparison test. Statistical significance is specified with $*p < 0.05$, $**p < 0.01$.

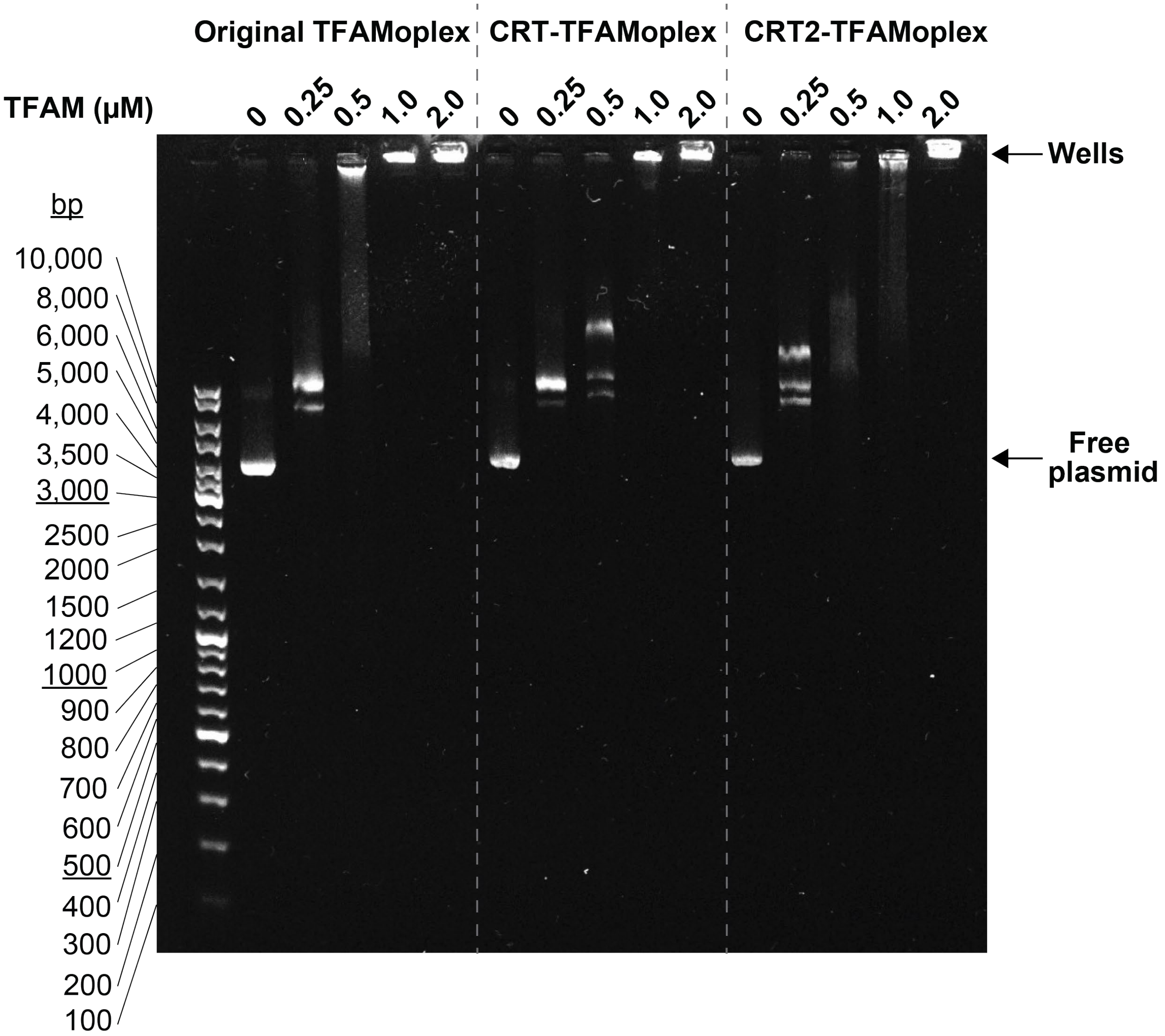

Figure S5. Gel mobility shift assay for the different TFAMoplex systems. Complexes were formed for 30 min in Buffer containing 2 mM $CaCl_2$ using increasing protein concentrations and 10 ng/µL DNA. Total TFAM concentrations are indicated. Original TFAMoplexes: PLC-TFAM (125—1000 nM), TFAM-VRK1 (125—1000 nM). CRT-TFAMoplexes: PLC-Cn-NB_{CD9}-Cn (15.6—125 nM), TFAM-Cc (109—875 nM), TFAM (16—125 nM), TFAM-VRK1 (125—1000 nM). CRT2-TFAMoplexes: PLC-Cn-NB_{CD9}-Cn (39—312 nM), TFAM-VRK1-Cc (250—2000 nM). Ten µL corresponding to 100 ng DNA were loaded per well. The gel wells and migration of the free plasmid are indicated by black arrows.

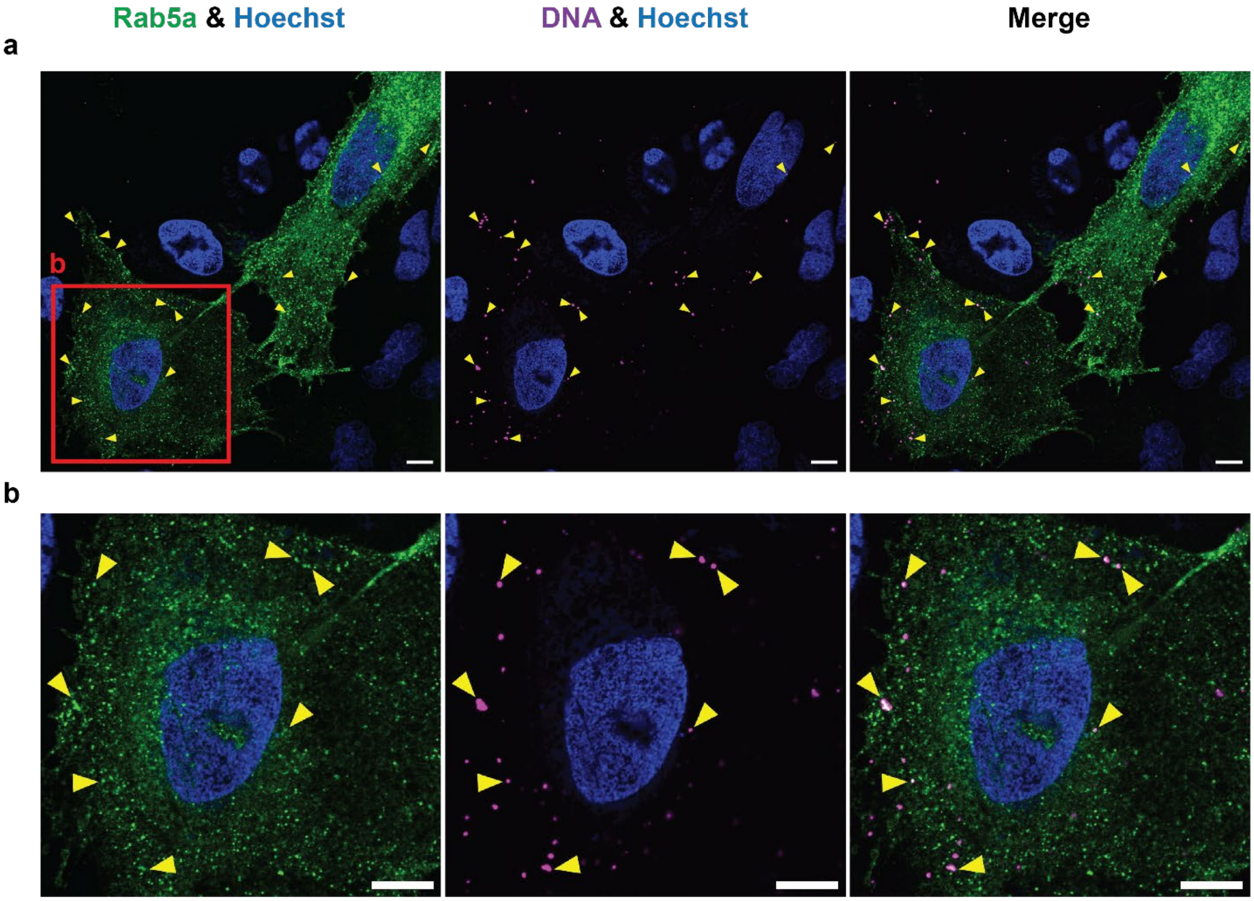

Figure S6. Colocalization of CRT2-TFAMoplexes and early endosomes. (a) Representative images displaying colocalization of Rab5a-GFP labelled early endosomes and CRT2-TFAMoplexes (10 nM PLC-Cn-NBCD9-Cn, 64 nM TFAM-VRK1-Cc) formed with 400 ng/mL Cy3-labelled pDNA. (b) Zoom-in region. HeLa cells were transduced one day prior to transfection with CellLight™ Early Endosomes-GFP BacMam 2.0. Cells were transfected with CRT2-TFAMoplexes in 99% FBS for 1 h. Cy3 is displayed in magenta. Early endosomes shown in green. Nuclei were stained with Hoechst (blue). Colocalization is indicated with yellow arrowheads and appears as white spots in the merged image. Images show a single z-slice. Scale bar: 10 µm.

Sample	1	2	3	4	5	6	7	8
P-Cn-NB$_{CD9}$-Cn (nM)	10	4	-	-	-	4	-	-
P-TFAM (nM)	-	-	32	4	-	-	-	-
P-NB$_{CD9}$-TFAM (nM)	-	-	-	-	4	-	-	-
P-Cn-NB$_{ALFA}$-Cn (nM)	-	-	-	-	-	-	4	-
TFAM-VRK1-Cc (nM)	64	-	-	-	-	-	-	-
TFAM-Cc (nM)	-	28	-	28	28	-	28	-
TFAM (nM)	-	4	-	-	-	32	4	-
TFAM-VRK1 (nM)	-	32	32	32	32	32	32	-
Lipofectamine	-	-	-	-	-	-	-	+

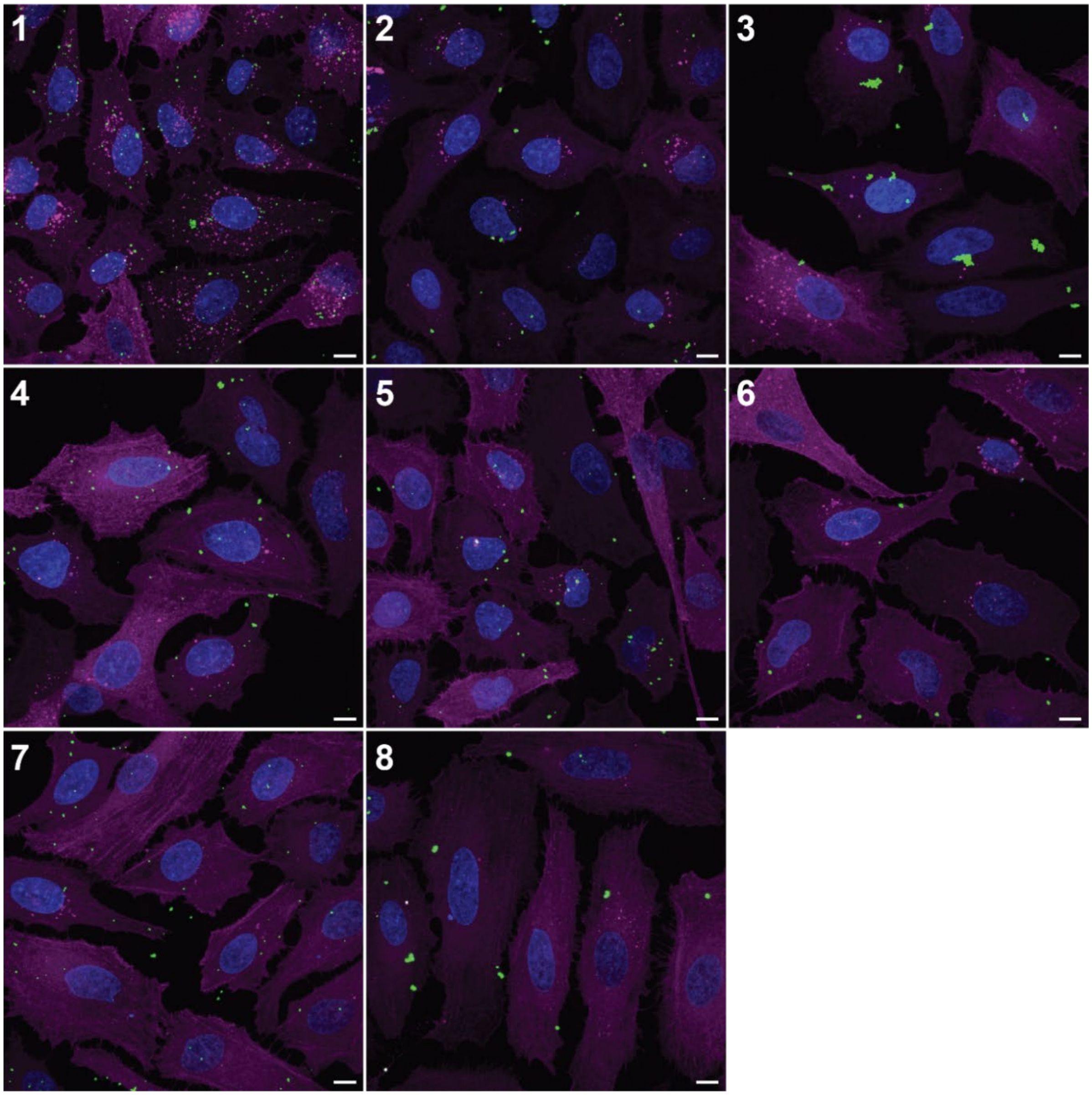

Figure S7. Combining TFAMoplexes with capture, release and targeting of PLC enhances endosomal disruption. Representative images displaying endosomal rupture (punctate mRuby3 fluorescence) in HeLa-Gal8-mRuby3 cells induced by the different TFAMoplex systems using MFP488-labelled DNA. Composition of the complexes is specified in the Table (top panel). Images show z-projections of maximum intensities. All complexes were added to cells in 99% FBS for 1 h with final 400 ng/mL DNA. MFP488 is displayed in green, mRuby3 is pseudocolored magenta. Nuclei were stained with Hoechst (blue). Scale bar 10 µm.

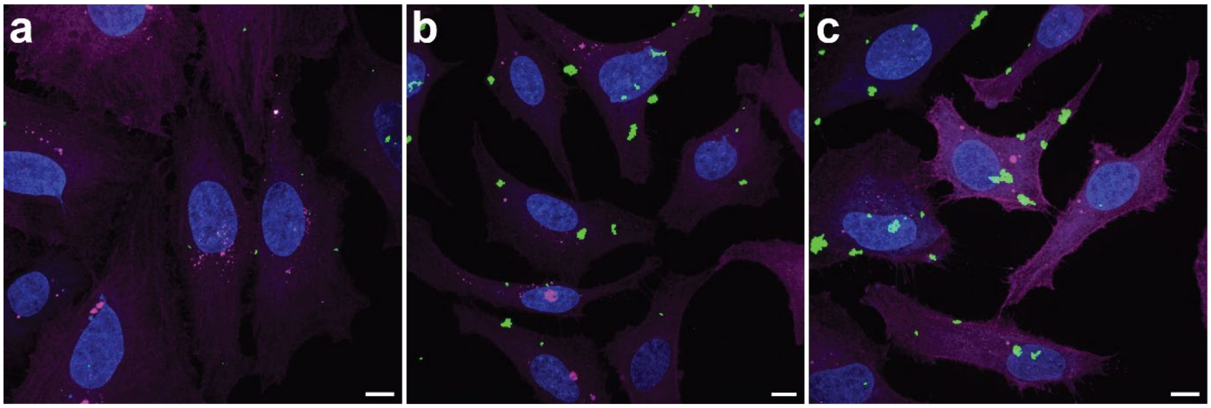

Figure S8. Applying increased concentrations of original TFAMoplexes on HeLa-Gal8-mRuby3 cells results in DNA aggregation. Representative images displaying endosomal rupture (punctate mRuby3 fluorescence) in HeLa-Gal8-mRuby3 cells induced by applying 3 different concentrations of original TFAMoplexes using MFP488-labelled DNA. (a) 16 nM PLC-TFAM, 16 nM TFAM-VRK1, 200 ng/mL DNA. (b) 32 nM PLC-TFAM, 32 nM TFAM-VRK1, 400 ng/mL DNA. (c) 64 nM PLC-TFAM, 64 nM TFAM-VRK1, 800 ng/mL DNA. Images show z-projections of maximum intensities. All complexes were added to cells in 99% FBS for 1 h (a and b) or 30 min (c). MFP488 is displayed in green, mRuby3 is pseudocolored magenta. Nuclei were stained with Hoechst (blue). Scale bar 10 µm.

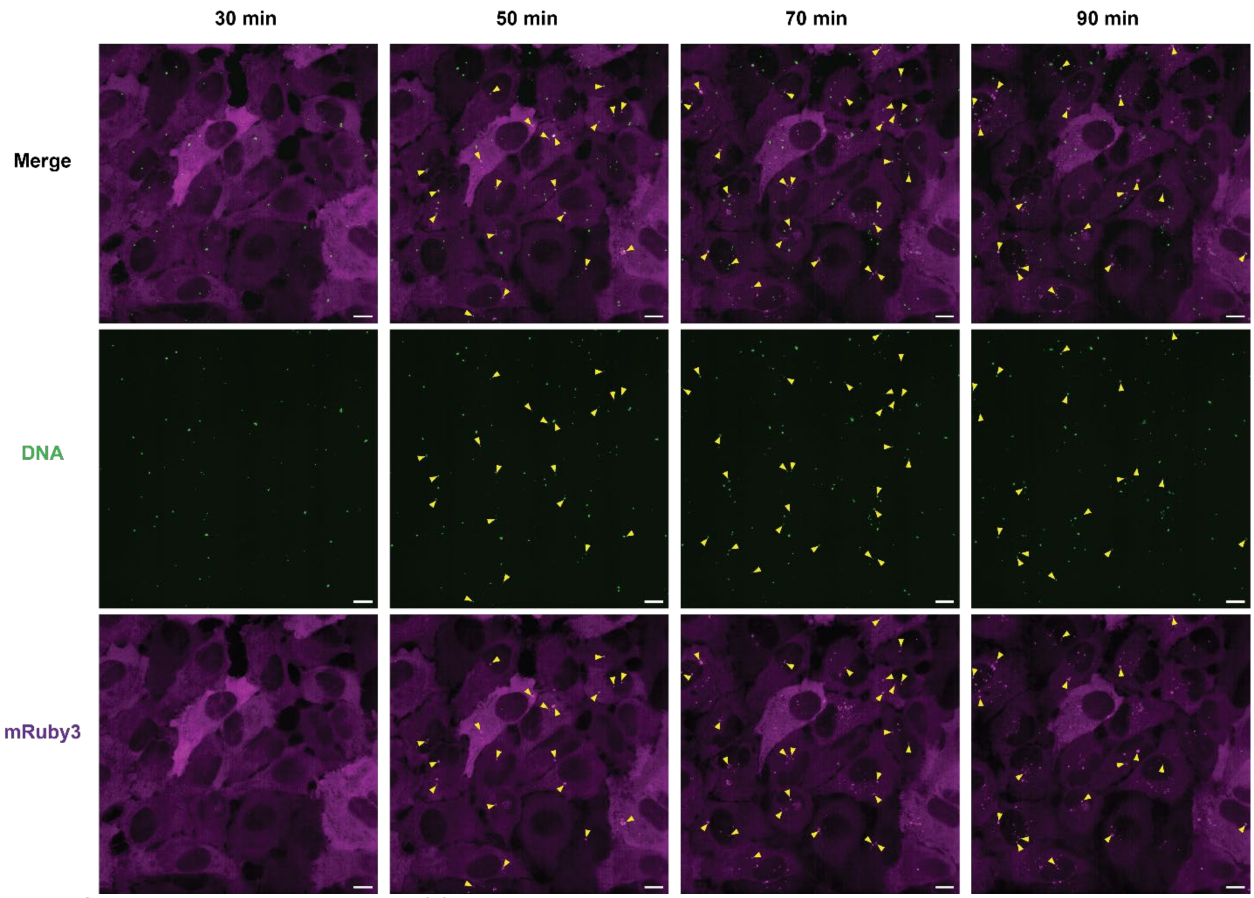

Figure S9. Live-cell time-lapse imaging of CRT2-TFAMoplex colocalization with endosomal rupture. Representative images displaying endosomal rupture (punctate mRuby3 fluorescence) in HeLa-Gal8-mRuby3 cells after addition of CRT2-TFAMoplexes using MFP488-labelled DNA (final concentrations: 10 nM P-Cn-NBCD9-Cn, 64 nM TFAM-VRK1-Cc and 400 ng/mL pDNA). Time-lapse imaging was initiated 30 min after transfection. The complexes were added to cells in complete growth medium without phenol red. Images show z-projections of maximum intensities. Time points indicate elapsed time after the addition of complexes to the cells. MFP488 is displayed in green, mRuby3 is pseudocolored magenta. Scale bar 10 µm.

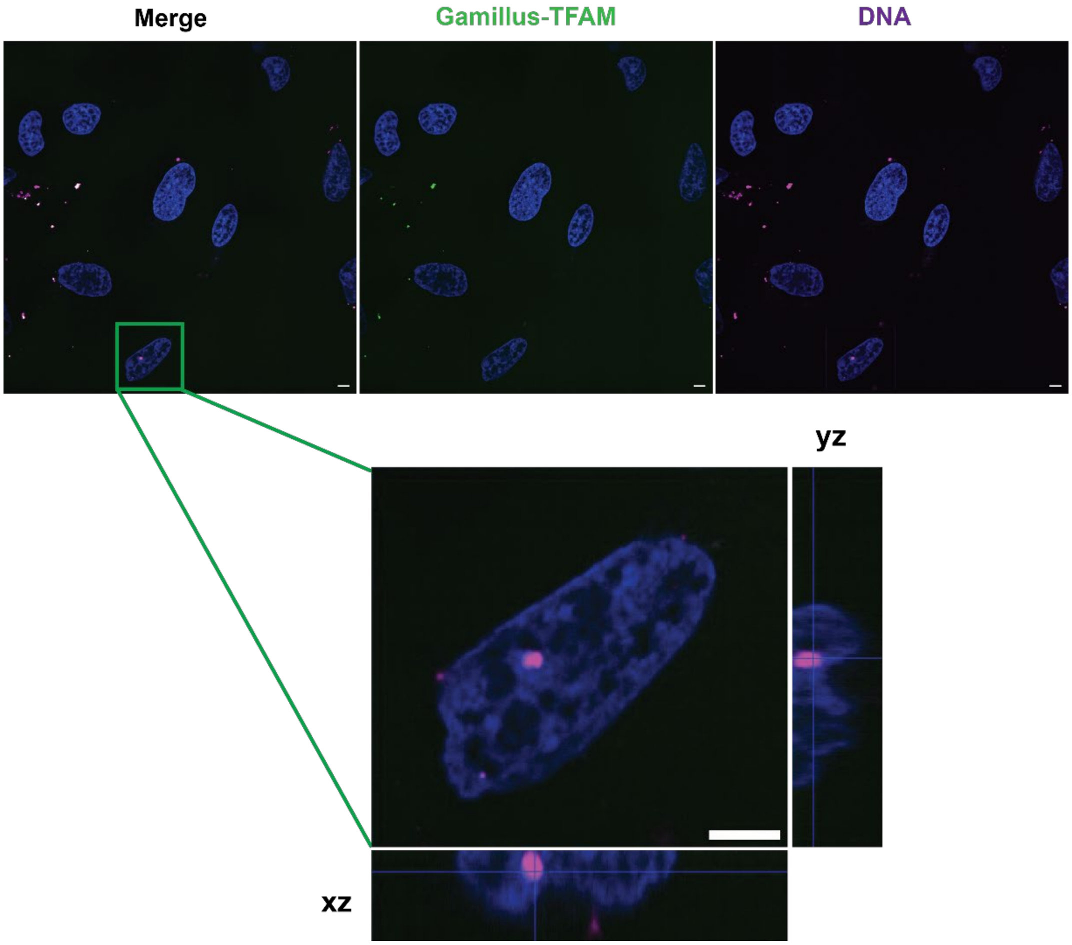

Figure S10. Nuclear colocalization of labelled CRT2-TFAMoplexes in HeLa cells. Representative images displaying HeLa cells transfected with Cy3- and Gamillus-labelled CRT2-TFAMoplexes. Final concentrations: 20 nM P-Cn-NBCD9-Cn, 64 nM TFAM-VRK1-Cc, 64 nM TFAM-Gamillus and 800 ng/mL Cy3-labelled pDNA. Cells were transfected for 30 min in full serum. Afterwards, the cells were washed and incubated in complete growth medium overnight. Images show a single z-slice. For the zoom-in region, the xy, yz and xz planes are shown. Cy3-labelled pDNA is displayed in magenta, Gamillus is displayed in green. Nuclei were stained with Hoechst (blue). Scale bar 5 µm.

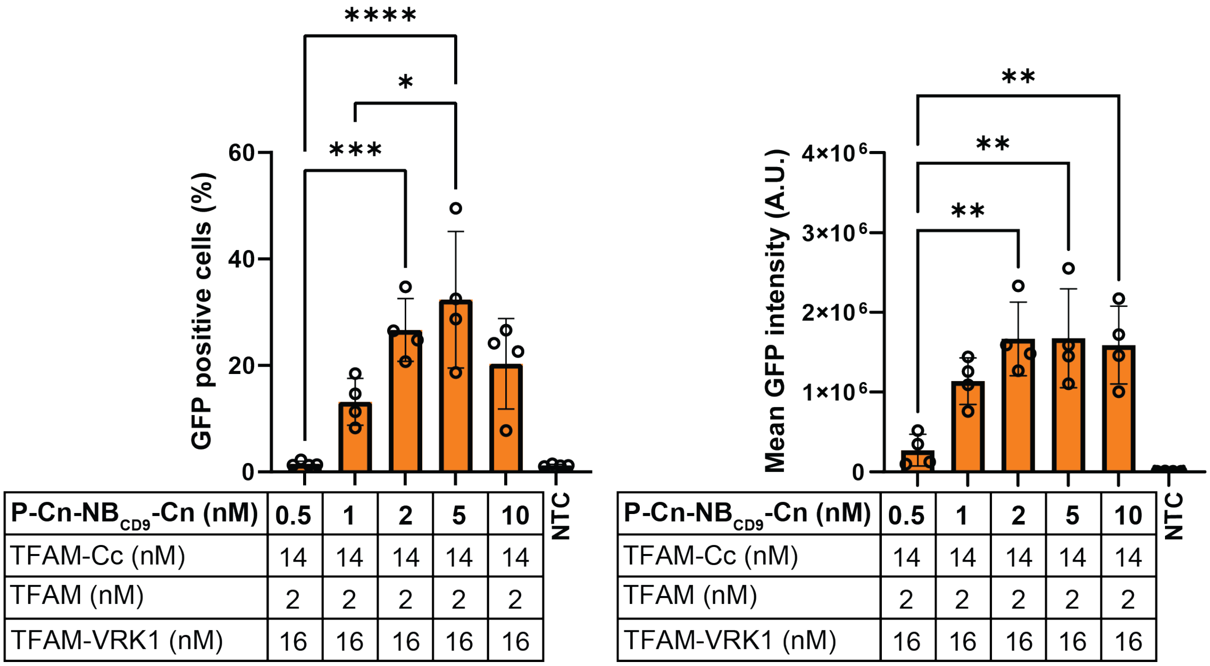

Figure S11. Transfection of HeLa cells with CRT-TFAMoplexes titrating PLC-Cn-NB$_{CD9}$-Cn. TFAMoplexes were formed with indicated protein concentrations and 200 ng/mL DNA. Cells were transfected with pDNA encoding for GFP in 99% FBS for 30 min and transfection efficiency was assessed the following day by flow cytometry quantifying GFP+ cells and mean GFP intensities. Bar plots specifying GFP positive cells and mean GFP intensities of GFP positive cells are shown. P, PLC; NTC, non-treated control. Data shown as mean ± SD with N = 4 independent experiments, each performed in technical triplicates. Data was analyzed using one-way ANOVA with Tukey's multiple comparison test. Statistical significance is specified with *p < 0.05, **p < 0.01, ***p < 0.001, ****p < 0.0001.

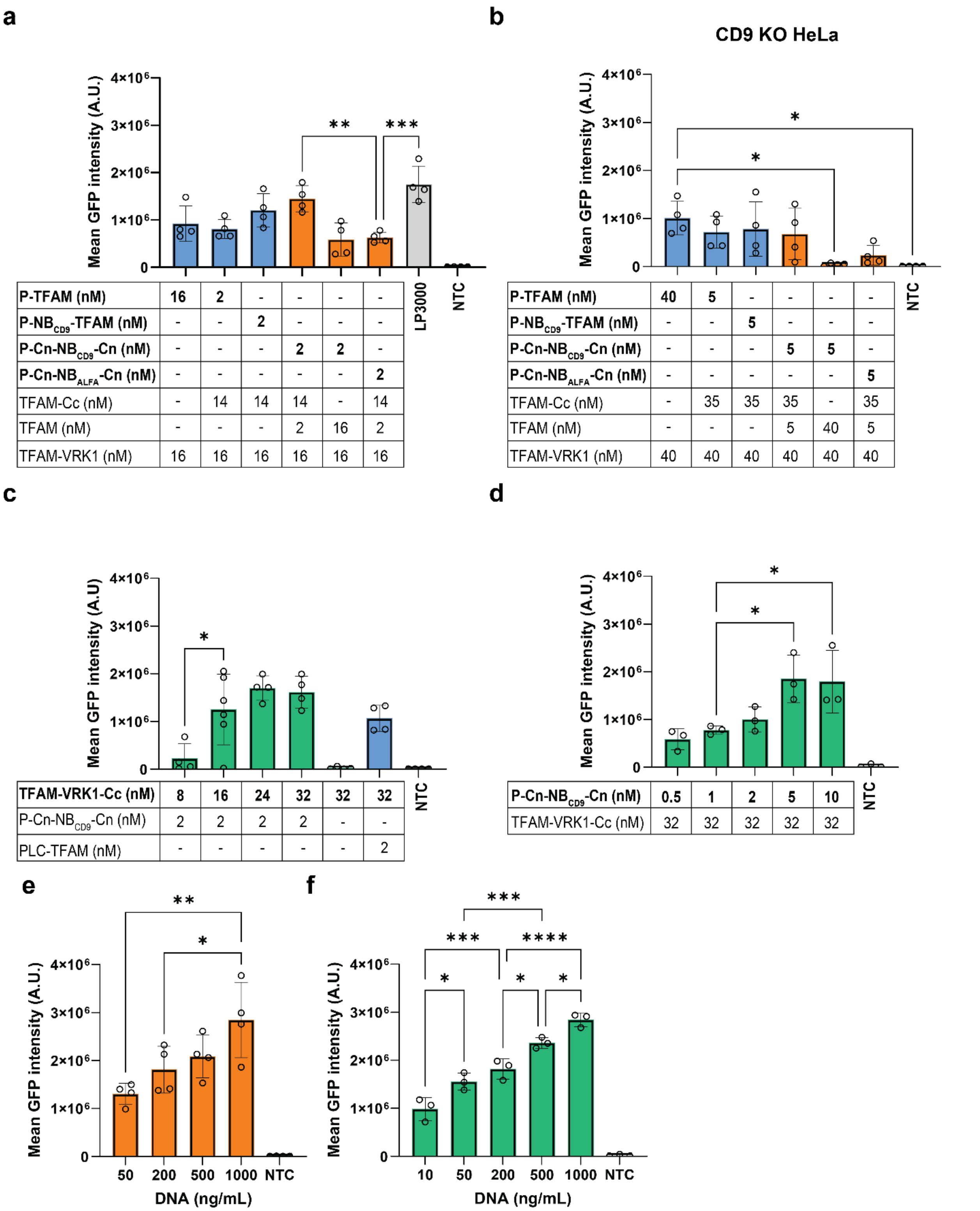

P-TFAM (nM)	16	2	-	-	-	-
P-NB$_{CD9}$-TFAM (nM)	-	-	2	-	-	-
P-Cn-NB$_{CD9}$-Cn (nM)	-	-	-	2	2	-
P-Cn-NB$_{ALFA}$-Cn (nM)	-	-	-	-	-	2
TFAM-Cc (nM)	-	14	14	14	-	14
TFAM (nM)	-	-	-	2	16	2
TFAM-VRK1 (nM)	16	16	16	16	16	16

P-TFAM (nM)	40	5	-	-	-	-
P-NB$_{CD9}$-TFAM (nM)	-	-	5	-	-	-
P-Cn-NB$_{CD9}$-Cn (nM)	-	-	-	5	5	-
P-Cn-NB$_{ALFA}$-Cn (nM)	-	-	-	-	-	5
TFAM-Cc (nM)	-	35	35	35	-	35
TFAM (nM)	-	-	-	5	40	5
TFAM-VRK1 (nM)	40	40	40	40	40	40

TFAM-VRK1-Cc (nM)	8	16	24	32	32	32
P-Cn-NB$_{CD9}$-Cn (nM)	2	2	2	2	-	-
PLC-TFAM (nM)	-	-	-	-	-	2

P-Cn-NB$_{CD9}$-Cn (nM)	0.5	1	2	5	10
TFAM-VRK1-Cc (nM)	32	32	32	32	32

Figure S12. Transfection experiments using TFAMoplexes incorporating the capture, release and targeting system. Bar plots specifying mean GFP intensities of GFP positive cells are shown. Transfection of HeLa (a) and CD9 KO HeLa (b) cells with different TFAMoplex systems or with Lipofectamine 3000™ using indicated protein concentrations and 200 ng/mL pDNA (HeLa) and 500 ng/mL pDNA (CD9 KO HeLa). (c) Transfection of HeLa cells with the CRT2-TFAMoplex system titrating TFAM-VRK1-Cc and TFAMoplexes containing PLC-TFAM as control using 200 ng/mL pDNA and indicated protein concentrations. (d) Transfection with CRT2-TFAMoplexes titrating PLC-Cn-NB$_{CD9}$-Cn using 200 ng/mL pDNA and indicated

protein concentrations on HeLa cells. (e) CRT-TFAMoplex dose escalation using PLC-Cn-NB_{CD9}-Cn (0.1—10 nM), TFAM-Cc (0.7—70 nM), TFAM (0.1—10 nM), TFAM-VRK1 (0.8—80 nM) and indicated DNA concentrations on HeLa cells. (f) CRT2-TFAMoplex dose escalation using PLC-Cn-NB_{CD9}-Cn (0.25—25 nM), TFAM-VRK1-Cc (1.6—160 nM) and indicated DNA concentrations on HeLa cells. For all experiments, cells were transfected with pDNA encoding for GFP in 99% FBS for 30 min and transfection efficiency was assessed the following day by flow cytometry quantifying GFP+ cells and mean GFP intensities. P, PLC; LP3000, Lipofectamine 3000™; NTC, non-treated control. Data shown as mean ± SD of N = 3-4 independent experiments, each performed in technical triplicates. Data was analyzed using one-way ANOVA with Tukey's multiple comparison test. Statistical significance is specified with *$p < 0.05$, **$p < 0.01$, ***$p < 0.001$, ****$p < 0.0001$.

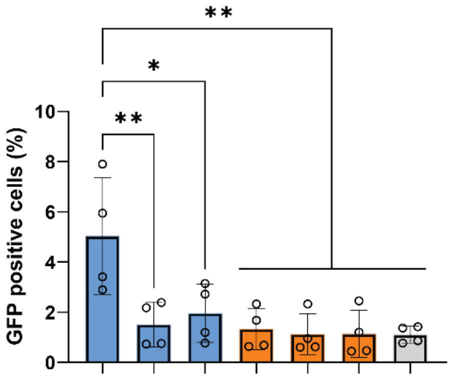

P-TFAM (nM)	16	2	-	-	-	-
P-NB$_{CD9}$-TFAM (nM)	-	-	2	-	-	-
P-Cn-NB$_{CD9}$-Cn (nM)	-	-	-	2	2	-
P-Cn-NB$_{ALFA}$-Cn (nM)	-	-	-	-	-	2
TFAM-Cc (nM)	-	14	14	14	-	14
TFAM (nM)	-	-	-	2	16	2
TFAM-VRK1 (nM)	16	16	16	16	16	16

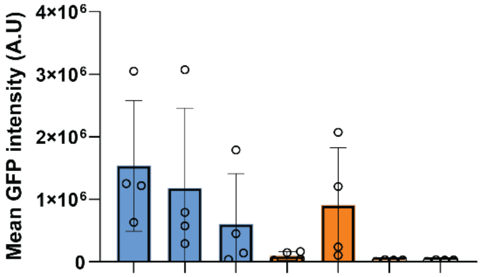

P-TFAM (nM)	16	2	-	-	-	-
P-NB$_{CD9}$-TFAM (nM)	-	-	2	-	-	-
P-Cn-NB$_{CD9}$-Cn (nM)	-	-	-	2	2	-
P-Cn-NB$_{ALFA}$-Cn (nM)	-	-	-	-	-	2
TFAM-Cc (nM)	-	14	14	14	-	14
TFAM (nM)	-	-	-	2	16	2
TFAM-VRK1 (nM)	16	16	16	16	16	16

Figure S13. Transfection of CD9 KO HeLa cells using TFAMoplexes incorporating the capture, release and targeting system. Bar plots specifying GFP positive cells and mean GFP intensities of GFP positive cells are shown. TFAMoplexes were formed with indicated protein concentrations and 200 ng/mL DNA. Cells were transfected with pDNA encoding for GFP in 99% FBS for 30 min and transfection efficiency was assessed the following day by flow cytometry quantifying GFP+ cells and mean GFP intensities. P, PLC; NTC, non-treated control. Data shown as mean ± SD of N = 4 independent experiments, each performed in technical triplicates. Data was analyzed using one-way ANOVA with Tukey's multiple comparison test. Statistical significance is specified with $^*p < 0.05$, $^{**}p < 0.01$.

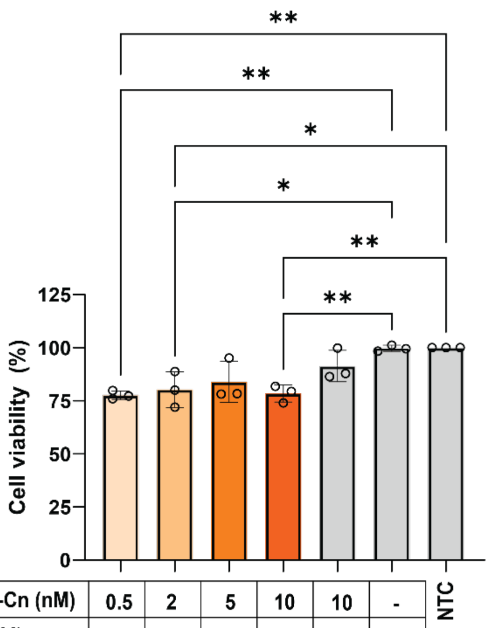

P-Cn-NB$_{CD9}$-Cn (nM)	0.5	2	5	10	10	-
TFAM-Cc (nM)	3.5	14	35	70	70	-
TFAM (nM)	0.5	2	5	10	10	-
TFAM-VRK1 (nM)	4	16	40	80	80	-
DNA (ng/mL)	50	200	500	1000	-	1000

Figure S14. Cell viability assay investigating CRT-TFAMoplex induced toxicity. Complexes were applied to HeLa cells in 99% FBS and incubated overnight. The next day, cell viability was measured with CellTiter 96® AQueous One Solution Cell Proliferation assay (MTS). P, PLC; NTC, non-treated control. From each replicate data set values obtained by applying 2% SDS (positive control) to cells was subtracted. Data were normalized to the maximal value of each respective replicate data set. Data shown as mean ± SD of N = 3 independent experiments, each performed in technical triplicates. Data was analyzed using one-way ANOVA with Tukey's multiple comparison test. Statistical significance is specified with *$p < 0.05$, **$p < 0.01$.

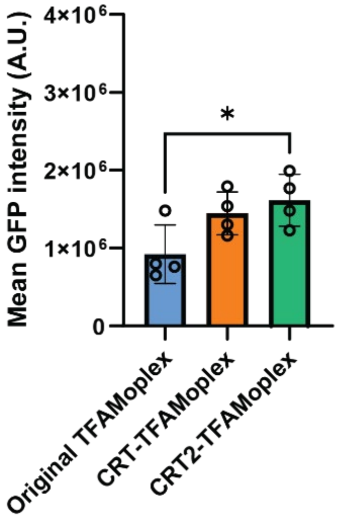

Figure S15. Comparison of mean GFP intensities obtained after HeLa cell transfection with the different TFAMoplex systems. Bar plots specifying mean GFP intensities of GFP positive cells for the indicated systems. Cells were transfected with pDNA encoding for GFP in 99% FBS for 30 min and transfection efficiency was assessed the following day by flow cytometry quantifying GFP+ cells and mean GFP intensities. Original TFAMoplexes (16 nM PLC-TFAM, 16 nM TFAM-VRK1), CRT-TFAMoplexes (2 nM PLC-Cn-NBCD9-Cn, 14 nM TFAM-Cc, 2 nM TFAM, 16 nM TFAM-VRK1), CRT2-TFAMoplexes (5 nM PLC-Cn-NBCD9-Cn, 32 nM TFAM-VRK1-Cc). All complexes were formed with final 200 ng/mL DNA on the cells. Data shown as mean ± SD of N = 4 independent experiments, each performed in technical triplicates. Data was analyzed using one-way ANOVA with Tukey's multiple comparison test. Statistical significance is specified with *$p < 0.05$.

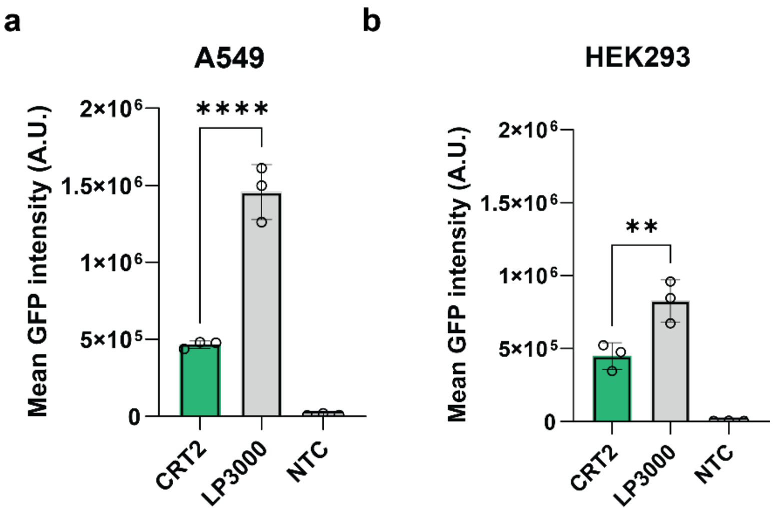

Figure S16. Mean GFP intensities obtained after transfection of A549 and HEK293 cells with CRT2-TFAMoplexes. (a) Transfection of A549 cells using CRT2-TFAMoplexes (10 nM PLC-Cn-NBCD9-Cn, 64 nM TFAM-VRK1-Cc) or LP3000 with 400 ng/mL pDNA. (b) Transfection of HEK293 cells using CRT2-TFAMoplexes (5 nM PLC-Cn-NBCD9-Cn, 32 nM TFAM-VRK1-Cc) or LP3000 with 200 ng/mL pDNA. Cells were transfected with pDNA encoding for GFP in 99% FBS for 30 min and transfection efficiency was assessed the following day by flow cytometry quantifying GFP+ cells. LP3000, Lipofectamine 3000™; NTC, non-treated control. Data shown as mean ± SD of N = 3 independent experiments, each performed in technical triplicates. Data was analyzed using one-way ANOVA with Tukey's multiple comparison test. Statistical significance is specified with **p < 0.01, ****p < 0.0001.

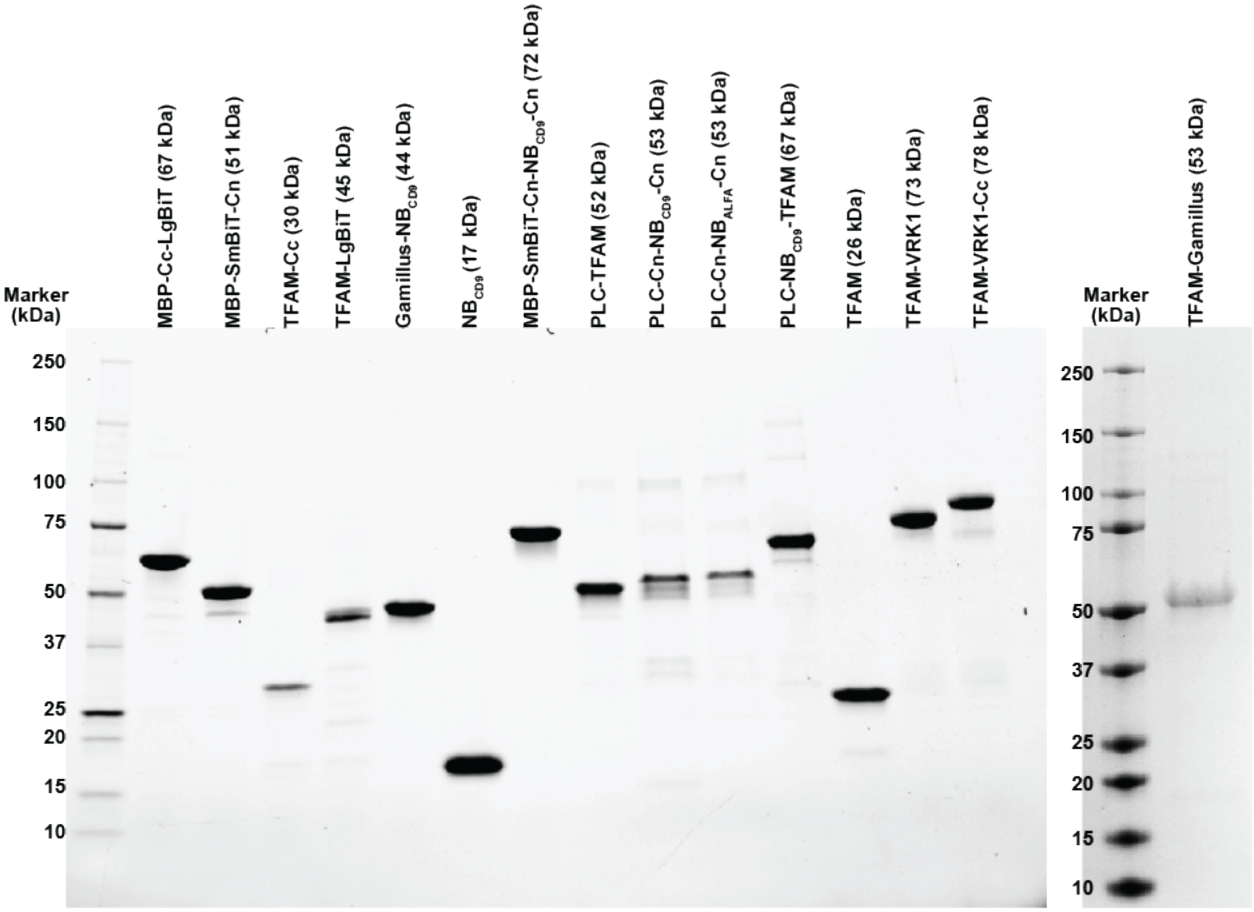

Figure S17. SDS-PAGE analysis of protein constructs. Molecular weight of each protein construct is specified. 1—2 µg protein were loaded per well. SDS gel was stained with Coomassie Brilliant Blue G-250.

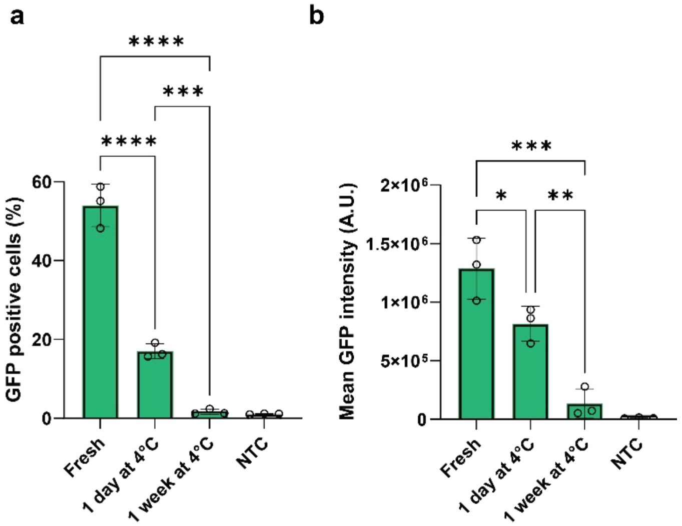

Figure S18. Transfection of HeLa cells with CRT2-TFAMoplexes. The complexes were formed in ≥80% FBS and incubated for 30 min at RT (fresh), one day at 4°C or 1 week at 4°C. HeLa cells were transfected using 200 ng/mL pDNA, 5 nM PLC-Cn-NBCD9-Cn and 32 nM TFAM-VRK1-Cc. Cells were transfected with pDNA encoding for GFP in 99% FBS for 30 min and transfection efficiency was assessed the following day by flow cytometry quantifying GFP+ cells. NTC, non-treated control. Data shown as mean ± SD of N = 3 independent experiments, each performed in technical triplicates. Data was analyzed using one-way ANOVA with Tukey's multiple comparison test. Statistical significance is specified with *$p < 0.05$, **$p < 0.01$, ***$p < 0.001$, ****$p < 0.0001$.

Supporting Movie 1. Live-cell time-lapse imaging of HeLa-Gal8-mRuby3 cells transfected with CRT2-TFAMoplexes using MFP488-labelled DNA. Applied final concentrations: 10 nM P-Cn-NBCD9-Cn, 64 nM TFAM-VRK1-Cc and 400 ng/mL pDNA. The complexes were added to cells in complete growth medium without phenol red. Endosomal rupture is represented by punctate mRuby3 fluorescence. Colocalization of endosomal rupture with MFP488-labelled DNA appears as white spots. Time-lapse imaging was initiated 30 min after transfection, acquiring z-stacks every 5 min. Movie displays total 60 min. MFP488 is displayed in green, mRuby3 is pseudocolored magenta. Scale bar 10 µm.

Supporting Tables

Table S1: Cloning strategy of constructs, DNA sequences, molecular weight (Mw) and molar extinction coefficient (ε) of constructs. Only the mutant TFAM$^{A105C, V109C}$ was used and is specified as TFAM for simplicity.

Construct	Cloning				DNA sequence	Mw (Da)	ε
	Backbone	Insert 1	Insert 2	Comments			
pET_MBP-PLC-TFAM	-	-	-	Produced as previously described [32]	ATGGGTAGCAGCCATCATCATCATCACCACCAAGCTTcgatGAAGATCGAAGAAGGTAAACTGGTAATCTGGAT<br>TAACGGCGATAAAGGCTATAACGGTCTCGCTGAAGTCGGTAAGAAATTCGAGAAAGATACCGGAATTAAAGT<br>CACCGTTGAGCATCCGGATAAACTGGAAGAGAAATTCCCACAGGTTGCGGCAACTGGCGATGGCCCTGACA<br>TTATCTTCTGGGCACACGACCGCTTTGGTGGCTACGCTCAATCTGGCCTGTTGGCTGAAATCACCCCGGACA<br>AAGCGTTCCAGGACAAGCTGTATCCGTTTACCTGGGATGCCGTACGTTACAACGGCAAGCTGATTGCTTACC<br>CGATCGCTGTTGAAGCGTTATCGCTGATTTATAACAAAGATCTGCTGCCGAACCCGCCAAAAACCTGGGAAG<br>AGATCCCGGCGCTGGATAAAGAACTGAAAGCGAAAGGTAAGAGCGCGCTGATGTTCAACCTGCAAGAACCG<br>TACTTCACCTGGCCGCTGATTGCTGCTGACGGGGGTTATGCGTTCAAGTATGAAAACGGCAAGTACGACATT<br>AAAGACGTGGGCGTGGATAACGCTGGCGCGAAAGCGGGTCTGACCTTCCTGGTTGACCTGATTAAAAACAA<br>ACACATGAATGCAGACACCGATTACTCCATCGCAGAAGCTGCCTTTAATAAAGGCGAAACAGCGATGACCAT<br>CAACGGCCCGTGGGCATGGTCCAACATCGACACCAGCAAAGTGAATTATGGTGTAACGGTACTGCCGACCTT<br>CAAGGGTCAACCATCCAAACCGTTCGTTGGCGTGCTGAGCGCAGGTATTAACGCCGCCAGTCCGAACAAAG<br>AGCTGGCAAAAGAGTTCCTCGAAAACTATCTGCTGACTGATGAAGGTCTGGAAGCGGTTAATAAAGACAAAC<br>CGCTGGGTGCCGTAGCGCTGAAGTCTTACGAGGAAGAGTTGGCGAAAGATCCACGTATTGCCGCCACCATG<br>GAAAACGCCCAGAAAGGTGAAATCATGCCGAACATCCCGCAGATGTCCGCTTTCTGGTATGCCGTGCGTACT<br>GCGGTGATCAACGCCGCCAGCGGTCGTCAGACTGTCGATGAAGCCCTGAAAGACGCGCAGACTAATTCGAG<br>CTCGAACAACAACAACAATAACAATAACAACAACCTCGGGctgcaGgCTTCAAGCGAAAATCTGTATTTCCAATG<br>GAGCGCGGACAATCCGACAAACACTGATGTAAACACCCATTATTGGCTTTTCAAGCAGGCTGAGAAAATTTTA<br>GCAAAAGACGTGAATCATATGCGCGCAAACCTGATGAACGAACTTAAGAAGTTTGATAAACAAATAGCCCAG<br>GGAATTTACGATGCCGACCACAAAAACCCCTATTATGACACTAGCACATTTCTGTCCCACTTCTATAACCCAG<br>ATCGCGATAACACGTACTTGCCTGGATTCGCAAATGCAAAGATTACGGGCGCTAAATATTTCAATCAAAGTGT<br>TACGGACTATCGAGAAGGTAAATTCGATACAGCCTTCTACAAATTAGGTCTGGCCATCCACTATTACACAGAT<br>ATTAGTCAACCTATGCACGCGAATAACTTCACGGCAATTAGCTATCCTCCGGGATATCATTGTGCATACGAAA<br>ACTACGTTGATACCATTAAACACAACTACCAGGCTACAGAAGATATGGTTGCCAAACGGTTTTGCAGCGACGA<br>CGTTAAAGATTGGTTATATGAAAACGCCAAACGTGCTAAAGCAGATTATCCGAAGATAGTAAACGCGAAAACA<br>AAAAAAAGCTACCTCGTGGGAAACTCTGAATGGAAAAAGGATACGGTCGAACCTACAGGAGCTCGCTTACGT<br>GATTCACAGCAGACGTTAGCCGGGTTCTTAGAATTTTGGTCTAAAAAGACAAATGAAAGCGGTGGTTCTGGT<br>GGATCGGGATCCatgtcatctgtcttgGCAAGTTGTCCAAAGAAACCTGTAAGTTCTTACCTTCGATTTTCTAAAGAAC<br>AACTACCCATATTTAAAGCTCAGAACCCAGATGCAAAAACTACAGAACTAATTAGAAGAATTGCCCAGCGTTG<br>GAGGGAACTTCCTGATTCAAAGAAAAAAATATATCAAGATGCTTATAGGGCGGAGTGGCAGGTATATAAAGAA<br>GAGATAAGCAGATTTAAAGAACAGCTAACTCCAAGTCAGATTATGTCTTTGGAAAAAGAAATCATGGACAAAC<br>ATTTAAAAAGGAAAGCTATGACAAAAAAAAAAGAGTTAACACTGCTTGGAAAACCAAAAAGACCTCGTTCAGC<br>TTATAACGTTTATGTAGCTGAAAGATTCCAAGAAGCTAAGGGTGATTCACCGCAGGAAAAGCTGAAGACTGTA<br>AAGGAAAACTGGAAAAATCTGTCTGACTCTGAAAAGGAATTATATATTCAGCATGCTAAAGAGGACGAAACTC<br>GTTATCATAATGAAATGAAGTCTTGGGAAGAACAAATGATTGAAGTTGGACGAAAGGATCTTCTACGTCGCAC<br>AATAAAGAAACAACGAAAATATGGTGCTGAGGAGTGTTaa	97,618	159,310
PLC-TFAM	-	-	-	Sequence corresponding to active PLC-TFAM (MBP cleaved off by TEV protease)	TGGAGCGCGGACAATCCGACAAACACTGATGTAAACACCCATTATTGGCTTTTCAAGCAGGCTGAGAAAATTT<br>TAGCAAAAGACGTGAATCATATGCGCGCAAACCTGATGAACGAACTTAAGAAGTTTGATAAACAAATAGCCCA<br>GGGAATTTACGATGCCGACCACAAAAACCCCTATTATGACACTAGCACATTTCTGTCCCACTTCTATAACCCA<br>GATCGCGATAACACGTACTTGCCTGGATTCGCAAATGCAAAGATTACGGGCGCTAAATATTTCAATCAAAGTG<br>TTACGGACTATCGAGAAGGTAAATTCGATACAGCCTTCTACAAATTAGGTCTGGCCATCCACTATTACACAGA<br>TATTAGTCAACCTATGCACGCGAATAACTTCACGGCAATTAGCTATCCTCCGGGATATCATTGTGCATACGAA<br>AACTACGTTGATACCATTAAACACAACTACCAGGCTACAGAAGATATGGTTGCCAAACGGTTTTGCAGCGACG<br>ACGTTAAAGATTGGTTATATGAAAACGCCAAACGTGCTAAAGCAGATTATCCGAAGATAGTAAACGCGAAAAC<br>AAAAAAAAGCTACCTCGTGGGAAACTCTGAATGGAAAAAGGATACGGTCGAACCTACAGGAGCTCGCTTACG<br>TGATTCACAGCAGACGTTAGCCGGGTTCTTAGAATTTTGGTCTAAAAAGACAAATGAAAGCGGTGGTTCTGGT<br>GGATCGGGATCCatgtcatctgtcttgGCAAGTTGTCCAAAGAAACCTGTAAGTTCTTACCTTCGATTTTCTAAAGAAC<br>AACTACCCATATTTAAAGCTCAGAACCCAGATGCAAAAACTACAGAACTAATTAGAAGAATTGCCCAGCGTTG<br>GAGGGAACTTCCTGATTCAAAGAAAAAAATATATCAAGATGCTTATAGGGCGGAGTGGCAGGTATATAAAGAA<br>GAGATAAGCAGATTTAAAGAACAGCTAACTCCAAGTCAGATTATGTCTTTGGAAAAAGAAATCATGGACAAAC<br>ATTTAAAAAGGAAAGCTATGACAAAAAAAAAAGAGTTAACACTGCTTGGAAAACCAAAAAGACCTCGTTCAGC<br>TTATAACGTTTATGTAGCTGAAAGATTCCAAGAAGCTAAGGGTGATTCACCGCAGGAAAAGCTGAAGACTGTA<br>AAGGAAAACTGGAAAAATCTGTCTGACTCTGAAAAGGAATTATATATTCAGCATGCTAAAGAGGACGAAACTC<br>GTTATCATAATGAAATGAAGTCTTGGGAAGAACAAATGATTGAAGTTGGACGAAAGGATCTTCTACGTCGCAC<br>AATAAAGAAACAACGAAAATATGGTGCTGAGGAGTGTTaa	52,856	91,220
pET_TFAM	-	-	-	Produced as previously described [32]	ATGGGTAGCAGCCATCATCATCATCACCACGGTTcgGGATCCATGTCATCTGTCTTGGCAAGTTGTCCAAAGA<br>AACCTGTAAGTTCTTACCTTCGATTTTCTAAAGAACAACTACCCATATTTAAAGCTCAGAACCCAGATGCAAAA<br>ACTACAGAACTAATTAGAAGAATTGCCCAGCGTTGGAGGGAACTTCCTGATTCAAAGAAAAAAATATATCAAG<br>ATGCTTATAGGtgcGAGTGGCAatgtTATAAAGAAGAGATAAGCAGATTTAAAGAgCAGtTgACTCCAAGTCAGATT<br>ATGTCTTTGGAAAAAGAAATCATGGACAAACATTTAAAAAGGAAAGCTATGACAAAAAAAAAAGAGTTAACACT<br>GCTTGGAAAACCAAAAAGACCTCGTTCAGCTTATAACGTTTATGTAGCTGAAAGATTCCAAGAAGCTAAGGGT<br>GATTCACCGCAGGAAAAGCTGAAGACTGTAAAGGAAAACTGGAAAAATCTGTCTGACTCTGAAAAGGAATTAT<br>ATATTCAGCATGCTAAAGAGGACGAAACTCGTTATCATAATGAAATGAAGTCTTGGGAAGAACAAATGATTGA<br>AGTTGGACGAAAGGATCTTCTACGTCGCACAATAAAGAAACAACGAAAATATGGTGCTGAGGAGTGTTCCGG<br>TGGATCAAGCGGCCGCAGTTAA	26,716	35,410

pET_TFAM-VRK1	-	-	-	Produced as previously described [32]	ATGGGGAGtTCACACCATCATCACCACCAcGGATCTGGTAGTATGAGTTCAGTGCTGGCTAGCTGTCCGAAAA<br>AACCAGTCTCTTCATATCTGCGTTTTTCAAAAGAGCAGTTGCCAATCTTTAAGGCCCAAAATCCAGATGCGAA<br>AACAACTGAGCTGATTAGACGCATAGCGCAACGGTGGAGAGAACTGCCGGACTCCAAGAAGAAGATTTATCA<br>GGACGCGTATCGCTGTGAGTGGCAATGCTATAAAGAAGAAATATCGCGTTTCAAAGAACAGCTGACCCCTAG<br>TCAGATTATGTCCCTTGAGAAAGAAATCATGGATAAACACCTGAAACGAAAAGCAATGACCAAGAAAAAAGAA<br>TTAACCTTACTGGGAAAACCAAAGCGGCCGCGCAGTGCATACAATGTTTATGTGGCTGAACGGTTTCAAGAG<br>GCAAAAGGCGATTCTCCTCAGGAGAAACTGAAAACGGTTAAAGAAAATTGGAAGAACCTCTCCGATTCAGAG<br>AAGGAACTGTATATCCAGCACGCTAAAGAGGATGAAACAAGATATCATAACGAAATGAAATCCTGGGAGGAG<br>CAGATGATTGAGGTAGGTCGGAAAGAcCTTCTACGTCGCACTATTAAGAAACAGCGCAAATACGGTGCTGAA<br>GAATGCAGTGGGGGTAGCTCCGGCCGTGGATCCATGCCCCGTGTGAAGGCGGCGCAGGCTGGACGGCAGT<br>CTTCAGCGAAGcgtCACCTCGCGGAGCAGTTTGCAGTTGGAGAAATTATCACTGATATGGCTAAAAAGGAGTG<br>GAAAGTGGGACTGCCTATTGGACAAGGTGGATTTGGTTGTATCTATCTGGCCGACATGAACTCTTCGGAATC<br>TGTGGGCTCAGATGCTCCCTGTGTAGTCAAAGTAGAACCTTCAGATAATGGGCCGCTGTTTACTGAACTGAA<br>ATTTTATCAAAGGGCTGCTAAACCTGAACAGATACAAAAATGGATACGGACTCGGAAATTGAAATATCTCGGC<br>GTACCAAAATATTGGGGTAGCGGACTTCATGATAAAAATGGGAAATCGTATCGTTTTATGATAATGGACCGGT<br>TCGGCTCGGACTTACAAAAAATTTACGAGGCGAACGCCAAACGGTTTAGCCGCAAGACTGTATTACAGCTGA<br>GCTTGCGCATTCTgGATATTCTTGAgTATATcCACGAACATGAATATGTTCATGGTGATATTAAGGCAagcAATTT<br>ATTATTGAACTATAAGAACCCGGATCAGGTATATTTGGTGGATTACGGTCTGGCATACCGCTACTGCCCGGA<br>GGGAGTACACAAAGAGTATAAGGAAGACCCAAAACGGTGTCATGAcGGAACCATCGAATTTACCTCGATAGA<br>CGCACATAACGGTGTCGCGCCCTCACGTCGTGGAGAcCTGGAAATCCTGGGATATTGCATGATTCAGTGGCT<br>GACGGGACACCTTCCgTGGGAGGATAATCTGAAAGATCCTAAGTATGTGCGAGACAGTAAGATTAGATACAG<br>GGAAAATATAGCCAGCCTGATGGATAAATGCTTTCCaGAAAAGAACAAACCGGGAGAAATCGCTAAATATATG<br>GAGACTGTCAAACTTTTGGATTACACCGAGAAACCGCTGTATGAAAACCTCCGCGATATtTTACTACAGGGCC<br>TGAAAGCCATTGGCAGTAAAGATGATGGCAAGTTAGAcCTGTCAGTGGTTGAAAACGGGGGTCTTAAAGCAA<br>AGACAATTACGAAAAAACGAAAGAAAGAGATTGAAGAATCAAAAGAACCAGGCGTTGAAGATACTGAATGGA<br>GCAATACACAGACAGAAGAGGCTATCCAGACGCGTTCCAGAACCCGCAAACGTGTTCAGAAGAGCGGTCCG<br>AAGAAGAAACGGAAAGTATAA	73,240	98,210
pET_MBP-Cc-LgBiT	pET_MBP-PLC-TFAM (PstI + XhoI)	Cc-LgBiT (PstI + XhoI)	-	Insert purchased from Twist Bioscience (South San Francisco, CA, USA)	ATGGGTAGCAGCCATCATCATCATCACCACCAAGCTTcgatGAAGATCGAAGAAGGTAAACTGGTAATCTGGAT<br>TAACGGCGATAAAGGCTATAACGGTCTCGCTGAAGTCGGTAAGAAATTCGAGAAAGATACCGGAATTAAAGT<br>CACCGTTGAGCATCCGGATAAACTGGAAGAGAAATTCCCACAGGTTGCGGCAACTGGCGATGGCCCTGACA<br>TTATCTTCTGGGCACACGACCGCTTTGGTGGCTACGCTCAATCTGGCCTGTTGGCTGAAATCACCCCGGACA<br>AAGCGTTCCAGGACAAGCTGTATCCGTTTACCTGGGATGCCGTACGTTACAACGGCAAGCTGATTGCTTACC<br>CGATCGCTGTTGAAGCGTTATCGCTGATTTATAACAAAGATCTGCTGCCGAACCCGCCAAAAACCTGGGAAG<br>AGATCCCGGCGCTGGATAAAGAACTGAAAGCGAAAGGTAAGAGCGCGCTGATGTTCAACCTGCAAGAACCG<br>TACTTCACCTGGCCGCTGATTGCTGCTGACGGGGGTTATGCGTTCAAGTATGAAAACGGCAAGTACGACATT<br>AAAGACGTGGGCGTGGATAACGCTGGCGCGAAAGCGGGTCTGACCTTCCTGGTTGACCTGATTAAAAACAA<br>ACACATGAATGCAGACACCGATTACTCCATCGCAGAAGCTGCCTTTAATAAAGGCGAAACAGCGATGACCAT<br>CAACGGCCCGTGGGCATGGTCCAACATCGACACCAGCAAAGTGAATTATGGTGTAACGGTACTGCCGACCTT<br>CAAGGGTCAACCATCCAAACCGTTCGTTGGCGTGCTGAGCGCAGGTATTAACGCCGCCAGTCCGAACAAAG<br>AGCTGGCAAAAGAGTTCCTCGAAAACTATCTGCTGACTGATGAAGGTCTGGAAGCGGTTAATAAAGACAAAC<br>CGCTGGGTGCCGTAGCGCTGAAGTCTTACGAGGAAGAGTTGGCGAAAGATCCACGTATTGCCGCCACCATG<br>GAAAACGCCCAGAAAGGTGAAATCATGCCGAACATCCCGCAGATGTCCGCTTTCTGGTATGCCGTGCGTACT<br>GCGGTGATCAACGCCGCCAGCGGTCGTCAGACTGTCGATGAAGCCCTGAAAGACGCGCAGACTAATTCGAG<br>CTCGAACAACAACAACAATAACAATAACAACAACCTCGGGcTGCAGGCTTCAAGCGAgAATCTGTATTTCCAA<br>AGCGGCGGTCTTAAAGGTCCGAATACTTTAGAtGATCTATTTCAAGAACTGGAcAAAAACGGTGACGGGGAGG<br>TCTCATTTGAGGAGTTTCAGGTGCTGGTGAAgAAGATtTCACAGGGCGGTGGCAGCGGTATGGTGTTTACTCT<br>TGAAGATTTTGTAGGTGATTGGGAGCAGACAGCGGCCTATAACCTGGATCAaGTTCTGGAACAAGGTGGAGT<br>TAGCAGCCTACTTCAGAAcCTGGCTGTTAGCGTGACGCCGATCCAGCGAATAGTTCGCTCCGGTGAAAATGC<br>CTTGAAAATTGATATACACGTGATCATCCCATACGAGGGATTGAGCGCAGATCAAATGGCCCAGATTGAAGAg<br>GTTTTTAAAGTCGTTTATCCAGTTGACGAcCATCATTTTAAAGTCATACTTCCGTATGGAACGCTTGTTATAGAT<br>GGGGTGACTCCGAACATGCTCAACTATTTCGGGAGGCCCTACGAAGGCATAGCAGTTTTCGATGGCAAGAAA<br>ATcACcGTGACtGGCACGCTGTGGAACGGCAACAAGATCATTGATGAACGCCTTATTACACCGGATGGTAGTA<br>TGCTTTTCCGGGTTACGATTAACTCTTAA	67,070	87,780
pET_MBP-SmBiT-Cn	pET_MBP-PLC-TFAM (PstI + XhoI)	SmBiT-Cn (PstI + XhoI)	-	Insert purchased from Twist Bioscience	ATGGGTAGCAGCCATCATCATCATCACCACCAAGCTTcgatGAAGATCGAAGAAGGTAAACTGGTAATCTGGAT<br>TAACGGCGATAAAGGCTATAACGGTCTCGCTGAAGTCGGTAAGAAATTCGAGAAAGATACCGGAATTAAAGT<br>CACCGTTGAGCATCCGGATAAACTGGAAGAGAAATTCCCACAGGTTGCGGCAACTGGCGATGGCCCTGACA<br>TTATCTTCTGGGCACACGACCGCTTTGGTGGCTACGCTCAATCTGGCCTGTTGGCTGAAATCACCCCGGACA<br>AAGCGTTCCAGGACAAGCTGTATCCGTTTACCTGGGATGCCGTACGTTACAACGGCAAGCTGATTGCTTACC<br>CGATCGCTGTTGAAGCGTTATCGCTGATTTATAACAAAGATCTGCTGCCGAACCCGCCAAAAACCTGGGAAG<br>AGATCCCGGCGCTGGATAAAGAACTGAAAGCGAAAGGTAAGAGCGCGCTGATGTTCAACCTGCAAGAACCG<br>TACTTCACCTGGCCGCTGATTGCTGCTGACGGGGGTTATGCGTTCAAGTATGAAAACGGCAAGTACGACATT<br>AAAGACGTGGGCGTGGATAACGCTGGCGCGAAAGCGGGTCTGACCTTCCTGGTTGACCTGATTAAAAACAA<br>ACACATGAATGCAGACACCGATTACTCCATCGCAGAAGCTGCCTTTAATAAAGGCGAAACAGCGATGACCAT<br>CAACGGCCCGTGGGCATGGTCCAACATCGACACCAGCAAAGTGAATTATGGTGTAACGGTACTGCCGACCTT<br>CAAGGGTCAACCATCCAAACCGTTCGTTGGCGTGCTGAGCGCAGGTATTAACGCCGCCAGTCCGAACAAAG<br>AGCTGGCAAAAGAGTTCCTCGAAAACTATCTGCTGACTGATGAAGGTCTGGAAGCGGTTAATAAAGACAAAC<br>CGCTGGGTGCCGTAGCGCTGAAGTCTTACGAGGAAGAGTTGGCGAAAGATCCACGTATTGCCGCCACCATG<br>GAAAACGCCCAGAAAGGTGAAATCATGCCGAACATCCCGCAGATGTCCGCTTTCTGGTATGCCGTGCGTACT<br>GCGGTGATCAACGCCGCCAGCGGTCGTCAGACTGTCGATGAAGCCCTGAAAGACGCGCAGACTAATTCGAG<br>CTCGAACAACAACAACAATAACAATAACAACAACCTCGGGcTGCAGgCTAGCAGTGAAAATCTGTATTTCCAAA<br>GCGGCGGaTCTGTGACCGGCTACCGGCTGTTCGAaGAgATaCTgGGCGGTGGCAGCGGTAGTACCAAGAAAT<br>CGCCaGAAGAATTAAAGCGTATATTTGAAAAATATGCCGCAAAAGAGGGCGATCCGGAcCAGCTTTCCAAGGA<br>CGAGCTTAAGTTGTTAATTCAAGCTGAgTTTCCTTCTTTAtaa	51,507	70,820

pET_MBP-SmBiT-Cn-NB_{CD9}-Cn	pET_MBP-PLC-TFAM (PstI + XhoI)	SmBiT-Cn-NB_{CD9}-Cn (PstI + XhoI)	-	Insert purchased from Twist Bioscience	ATGGGTAGCAGCCATCATCATCATCACCACCAAGCTTcgatGAAGATCGAAGAAGGTAAACTGGTAATCTGGAT<br>TAACGGCGATAAAGGCTATAACGGTCTCGCTGAAGTCGGTAAGAAATTCGAGAAAGATACCGGAATTAAAGT<br>CACCGTTGAGCATCCGGATAAACTGGAAGAGAAATTCCCACAGGTTGCGGCAACTGGCGATGGCCCTGACA<br>TTATCTTCTGGGCACACGACCGCTTTGGTGGCTACGCTCAATCTGGCCTGTTGGCTGAAATCACCCCGGACA<br>AAGCGTTCCAGGACAAGCTGTATCCGTTTACCTGGGATGCCGTACGTTACAACGGCAAGCTGATTGCTTACC<br>CGATCGCTGTTGAAGCGTTATCGCTGATTTATAACAAAGATCTGCTGCCGAACCCGCCAAAAACCTGGGAAG<br>AGATCCCGGCGCTGGATAAAGAACTGAAAGCGAAAGGTAAGAGCGCGCTGATGTTCAACCTGCAAGAACCG<br>TACTTCACCTGGCCGCTGATTGCTGCTGACGGGGGTTATGCGTTCAAGTATGAAAACGGCAAGTACGACATT<br>AAAGACGTGGGCGTGGATAACGCTGGCGCGAAAGCGGGTCTGACCTTCCTGGTTGACCTGATTAAAAACAA<br>ACACATGAATGCAGACACCGATTACTCCATCGCAGAAGCTGCCTTTAATAAAGGCGAAACAGCGATGACCAT<br>CAACGGCCCGTGGGCATGGTCCAACATCGACACCAGCAAAGTGAATTATGGTGTAACGGTACTGCCGACCTT<br>CAAGGGTCAACCATCCAAACCGTTCGTTGGCGTGCTGAGCGCAGGTATTAACGCCGCCAGTCCGAACAAAG<br>AGCTGGCAAAAGAGTTCCTCGAAAACTATCTGCTGACTGATGAAGGTCTGGAAGCGGTTAATAAAGACAAAC<br>CGCTGGGTGCCGTAGCGCTGAAGTCTTACGAGGAAGAGTTGGCGAAAGATCCACGTATTGCCGCCACCATG<br>GAAAACGCCCAGAAAGGTGAAATCATGCCGAACATCCCGCAGATGTCCGCTTTCTGGTATGCCGTGCGTACT<br>GCGGTGATCAACGCCGCCAGCGGTCGTCAGACTGTCGATGAAGCCCTGAAAGACGCGCAGACTAATTCGAG<br>CTCGAACAACAACAACAATAACAATAACAACAACCTCGGGcTGCAGgCTAGCAGTGAAAATCTGTATTTCCAAA<br>GCGGCGGaTCTGTGACCGGCTACCGGCTGTTCGAaGAgATaCTgGGCGGTGGCAGCGGTTCCACGAAAAAAA<br>GCCCAGAAGAACTTAAACGCATATTCGAAAAGTATGCTGCCAAGGAAGGCGACCCGGATCAGCTGTCTAAAG<br>ATGAGCTGAAACTTCTCATTCAGGCCGAGTTCCCTAGCTTAGGCGGTAGCGGTGGaTCTGAAGTGCAACTGG<br>TTGAATCAGGAGGCGGTTTAGTGCAGGCTGGAGGGAGTCTGCGTCTGTCTTGTGCCGCATCTGGCCGTACA<br>TTTAGCGATTATGTGATGGGCTGGTTtCGCCAAGCACCAGGGAAAGAACGCACATTCGTGGCGCGGATCGGT<br>TGGAGTGGTGACCTGACGTACTATGCAGACAGTGTTAAAGGCAGATTTACTATCTCTCGCGACAACGCAAAG<br>AATACGGTGTACTTGCAAATGAACTCATTAAAACCGGAGGACACTGCCATTTATTACTGTGCAGCAGATGAAC<br>GGTGGGGCACAGGTGGTAAATTCGACTATTGGGGACAAGGAACACAAGTTACAGTAAGCGGTGGCTCCGGA<br>GGCAGCAGTACCAAGAAGTCGCCGGAAGAATTAAAGCGTATCTTCGAGAAATACGCCGCAAAAGAGGGCGA<br>TCCGGAcCAGCTTTCCAAGGACGAGCTTAAACTGTTAATTCAAGCTGAgTTTCCTTCTTTAGCTAGCGCTTGGT<br>CGCACCCGCAGTTTGAGAAATaa	71,552	110,365
pET_TFAM-Cc	pET_TFAM-sh2 (BamHI + XhoI)	Cc (BamHI + XhoI)	-	pET_TFAM-sh2 was obtained from Dr. Michael Burger; insert purchased from Twist Bioscience	ATGGGTAGCAGCCATCATCATCATCACCACTCTGCAGGAGGTACCGGACAAGCTTTGATGTCATCTGTCTTG<br>GCAAGTTGTCCAAAGAAACCTGTAAGTTCTTACCTTCGATTTTCTAAAGAACAACTACCCATATTTAAAGCTCA<br>GAACCCAGATGCAAAAACTACAGAACTAATTAGAAGAATTGCCCAGCGTTGGAGGGAACTTCCTGATTCAAA<br>GAAAAAAATATATCAAGATGCTTATAGGTGCGAGTGGCAATGTTATAAAGAAGAGATAAGCAGATTTAAAGAG<br>CAGTTGACTCCAAGTCAGATTATGTCTTTGGAAAAAGAAATCATGGACAAACATTTAAAAAGGAAAGCTATGA<br>CAAAAAAAAAAGAGTTAACACTGCTTGGAAAACCAAAAAGACCTCGTTCAGCTTATAACGTTTATGTAGCTGAA<br>AGATTCCAAGAAGCTAAGGGTGATTCACCGCAGGAAAAGCTGAAGACTGTAAAGGAAAACTGGAAAAATCTG<br>TCTGACTCTGAAAAGGAATTATATATTCAGCATGCTAAAGAGGACGAAACTCGTTATCATAATGAAATGAAGTC<br>TTGGGAAGAACAAATGATTGAAGTTGGACGAAAGGATCTTCTACGTCGCACAATAAAGAAACAACGAAAATAT<br>GGTGCTGAGGAGTGTGGATCCGGCGGTTCTCTTAAAGGTCCGAATACTTTAGAtGACCTATTTCAAGAACTGG<br>AcAAAAACGGTGACGGGGAGGTCTCATTTGAGGAGTTTCAGGTGCTGGTGAAgAAGATtTCACAGTAA	30,904	35,410
pET_TFAM-Gamillus	pET_TFAM-sh2 (BamHI + XhoI)	Gamillus (BamHI + XhoI)	-	pET_TFAM-sh2 was obtained from Dr. Michael Burger; insert purchased from Twist Bioscience	ATGGGTAGCAGCCATCATCATCATCACCACTCTGCAGGAGGTACCGGACAAGCTTTGATGTCATCTGTCTTGG<br>CAAGTTGTCCAAAGAAACCTGTAAGTTCTTACCTTCGATTTTCTAAAGAACAACTACCCATATTTAAAGCTCAGA<br>ACCCAGATGCAAAAACTACAGAACTAATTAGAAGAATTGCCCAGCGTTGGAGGGAACTTCCTGATTCAAAGAA<br>AAAAATATATCAAGATGCTTATAGGTGCGAGTGGCAATGTTATAAAGAAGAGATAAGCAGATTTAAAGAGCAGTT<br>GACTCCAAGTCAGATTATGTCTTTGGAAAAAGAAATCATGGACAAACATTTAAAAAGGAAAGCTATGACAAAAAA<br>AAAAGAGTTAACACTGCTTGGAAAACCAAAAAGACCTCGTTCAGCTTATAACGTTTATGTAGCTGAAAGATTCC<br>AAGAAGCTAAGGGTGATTCACCGCAGGAAAAGCTGAAGACTGTAAAGGAAAACTGGAAAAATCTGTCTGACTC<br>TGAAAAGGAATTATATATTCAGCATGCTAAAGAGGACGAAACTCGTTATCATAATGAAATGAAGTCTTGGGAAGA<br>ACAAATGATTGAAGTTGGACGAAAGGATCTTCTACGTCGCACAATAAAGAAACAACGAAAATATGGTGCTGAGG<br>AGTGTGGATCCGGCGGTTCTATGGTGAGCAAGGGCGAGGAgGCCAGTGGACGCGCCCTGTTTCAGTATCCC<br>ATGACCTCTAAAATCGAGTTGAATGGGGAAATCAACGGAAAAAAATTTAAAGTTGCAGGTGAAGGATTTACACC<br>TTCTTCCGGGCGATTCAAcATGCATGCcTATTGCACTACGGGCGACCTGCCGATGTCATGGGTGGTTATAGCAA<br>GCCCACTaCAGTATGGGTTTCACATGTTCGCGCATTATCCCGAAGATATTACCCATTTCTTCCAAGAATGTTTTC<br>CGGGTTCATACACGCTAGATCGTACCCTGCGTATGGAAGGTGATGGGACGCTTACAACACACCACGAATATAG<br>TCTGGAAGATGGTTGcGTAACTTCaAAGACTACCTTAAATGCGAGCGGGTTTGATCCGAAAGGTGCTACAATGA<br>CAAAgTCCTTCGTGAAACAGTTACCCAATGAAGTCAAGATAACTCCGCACGGACCGAACGGTATTAGATTGAC<br>GTCTACAGTCCTTTACCTGAAAGAAGACGGTACTATTCAGATCGGGACCCAAGATTGCATTGTGACCCCAGTT<br>GGCGGTCGTAAAGTgACACAACCAAAGGCTCACTTCCTTCACACCCAGATAATTCAGAAAAAGGACCCCAATG<br>ATACCCGCGACCACATAGTGCAAACCGAACTGGCAGTGGCAGGTAATCTGTGGCATGGCATGGACGAGCTGT<br>ACAAGTAA	53,369	58,330
pET_TFAM-LgBiT	pET_TFAM-VRK1 (BamHI + XhoI)	LgBiT (BamHI + XhoI)	-	Insert purchased from GeneArt (Thermo Fisher Scientific, St. Louis, MO, USA)	ATGGATAAATATCAACATCATCATCATCACCACTCTGCAGGAGGTACCGGACAAGCTTTGATGTCATCTGTCT<br>TGGCAAGTTGTCCAAAGAAACCTGTAAGTTCTTACCTTCGATTTTCTAAAGAACAACTACCCATATTTAAAGCT<br>CAGAACCCAGATGCAAAAACTACAGAACTAATTAGAAGAATTGCCCAGCGTTGGAGGGAACTTCCTGATTCAA<br>AGAAAAAAATATATCAAGATGCTTATAGGTGCGAGTGGCAATGTTATAAAGAAGAGATAAGCAGATTTAAAGA<br>GCAGTTGACTCCAAGTCAGATTATGTCTTTGGAAAAAGAAATCATGGACAAACATTTAAAAAGGAAAGCTATG<br>ACAAAAAAAAAAGAGTTAACACTGCTTGGAAAACCAAAAAGACCTCGTTCAGCTTATAACGTTTATGTAGCTGA<br>AAGATTCCAAGAAGCTAAGGGTGATTCACCGCAGGAAAAGCTGAAGACTGTAAAGGAAAACTGGAAAAATCT<br>GTCTGACTCTGAAAAGGAATTATATATTCAGCATGCTAAAGAGGACGAAACTCGTTATCATAATGAAATGAAGT<br>CTTGGGAAGAACAAATGATTGAAGTTGGACGAAAGGATCTTCTACGTCGCACAATAAAGAAACAACGAAAATA<br>TGGTGCTGAGGAGTGTGGGAATTCAGGGTCGACGGGTGGATCCTCTGCAGggATGGTGTTTACTCTTGAaGA<br>TTTTGTAGGTGATTGGGAGCAGACAGCGGCCTATAACCTGGATCAGGTTCTGGAACAAGGTGGAGTTAGCAG<br>cCTACTTCAGAATCTGGCTGTTAGCGTGACGCCgATCCAGCGAATAGTTCGCTCCGGTGAAAATGCCTTGAAA<br>ATTGATATACACGTGATCATCCCATACGAGGGATTGAGCGCAGATCAAATGGCCCAGATTGAAGAAGTTTTTA<br>AAGTCGTTTATCCAGTTGACGATCATCATTTTAAAGTCATACTTCCGTATGGAACGCTTGTTATAGATGGGGTG<br>ACTCCGAACATGCTCAACTATTTCGGGAGGCCcTACGAAGGCATAGCAGTTTTCGATGGCAAGAAAATAACTG<br>TGACGGGCACGCTGTGGAACGGCAACAAGATCATTGATGAACGCCTTATTACACCGGATGGTAGtATGCTTTT<br>CCGGGTTACGATTAACTCTggGTCGACctga	45,675	56,840

pET_Gamillus-NB$_{CD9}$	pET_MBP-PLC-TFAM (HindIII + XhoI)	Gamillus (HindIII + BamHI)	NB$_{CD9}$ (BamHI + XhoI)	Inserts purchased from Twist Bioscience	ATGGGTAGCAGCCATCATCATCATCACCACCAAGCTTCTATGGTGAGCAAGGGCGAGGAgGCCAGTGGACG<br>CGCCCTGTTTCAGTATCCCATGACCTCTAAAATCGAGTTGAATGGGGAAATCAACGGAAAAAAATTTAAAGTT<br>GCAGGTGAAGGATTTACACCTTCTTCCGGGCGATTCAAcATGCATGCcTATTGCACTACGGGCGACCTGCCG<br>ATGTCATGGGTGGTTATAGCAAGCCCACTaCAGTATGGGTTTCACATGTTCGCGCATTATCCCGAAGATATTA<br>CCCATTTCTTCCAAGAATGTTTTCCGGGTTCATACACGCTAGATCGTACCCTGCGTATGGAAGGTGATGGGA<br>CGCTTACAACACACCACGAATATAGTCTGGAAGATGGTTGcGTAACTTCaAAGACTACCTTAAATGCGAGCGG<br>GTTTGATCCGAAAGGTGCTACAATGACAAAgTCCTTCGTGAAACAGTTACCCAATGAAGTCAAGATAACTCCG<br>CACGGACCGAACGGTATTAGATTGACGTCTACAGTCCTTTACCTGAAAGAAGACGGTACTATTCAGATCGGG<br>ACCCAAGATTGCATTGTGACCCCAGTTGGCGGTCGTAAAGTgACACAACCAAAGGCTCACTTCCTTCACACC<br>CAGATAATTCAGAAAAAGGACCCCAATGATACCCGCGACCACATAGTGCAAACCGAACTGGCAGTGGCAGGT<br>AATCTGTGGCATGGCATGGACGAGCTGTACAAGGGgAATTCaGGGTCGACGGGTgGATCCGGTGGTTCCGAA<br>GTGCAACTGGTTGAATCAGGAGGCGGTTTAGTGCAGGCTGGAGGGAGTCTGCGTCTGTCTTGTGCCGCATC<br>TGGCCGTACATTTAGCGATTATGTGATGGGCTGGTTtCGCCAAGCACCAGGGAAAGAACGCACATTCGTGGC<br>GCGGATCGGTTGGAGTGGTGACCTGACGTACTATGCAGACAGTGTTAAAGGCAGATTTACTATCTCTCGCGA<br>CAACGCAAAGAATACGGTGTACTTGCAAATGAACTCATTAAAACCGGAGGACACTGCCATTTATTACTGTGCA<br>GCAGATGAACGGTGGGGCACAGGTGGTAAATTCGACTATTGGGGACAAGGAACACAAGTTACAGTAAGCGG<br>CGGTAGCGGTGGCTCTGCTAGCGCTTGGTCGCACCCGCAGTTTGAGAAATaa	43,761	61,225
pET_NB$_{CD9}$	pET_MBP-PLC-TFAM (HindIII + XhoI)	NB$_{CD9}$ (HindIII + XhoI)	-	Insert purchased from Twist Bioscience	ATGGGTAGCAGCCATCATCATCATCACCACCAAGCTTCTGGTgGATCCGGTGGTTCCGAAGTGCAACTGGTT<br>GAATCAGGAGGCGGTTTAGTGCAGGCTGGAGGGAGTCTGCGTCTGTCTTGTGCCGCATCTGGCCGTACATT<br>TAGCGATTATGTGATGGGCTGGTTtCGCCAAGCACCAGGGAAAGAACGCACATTCGTGGCGCGGATCGGTTG<br>GAGTGGTGACCTGACGTACTATGCAGACAGTGTTAAAGGCAGATTTACTATCTCTCGCGACAACGCAAAGAA<br>TACGGTGTACTTGCAAATGAACTCATTAAAACCGGAGGACACTGCCATTTATTACTGTGCAGCAGATGAACGG<br>TGGGGCACAGGTGGTAAATTCGACTATTGGGGACAAGGAACACAAGTTACAGTAAGCGGCGGTAGCGGTGG<br>CTCTGCTAGCGCTTGGTCGCACCCGCAGTTTGAGAAATaa	16,730	38,055
pET_MBP-PLC-Cn-NB$_{CD9}$-Cn	pET_MBP-PLC-TFAM (BamHI + XhoI)	Cn-NB$_{CD9}$-Cn (BamHI + XhoI)	-	Insert purchased from Twist Bioscience	ATGGGTAGCAGCCATCATCATCATCACCACCAAGCTTcgatGAAGATCGAAGAAGGTAAACTGGTAATCTGGAT<br>TAACGGCGATAAAGGCTATAACGGTCTCGCTGAAGTCGGTAAGAAATTCGAGAAAGATACCGGAATTAAAGT<br>CACCGTTGAGCATCCGGATAAACTGGAAGAGAAATTCCCACAGGTTGCGGCAACTGGCGATGGCCCTGACA<br>TTATCTTCTGGGCACACGACCGCTTTGGTGGCTACGCTCAATCTGGCCTGTTGGCTGAAATCACCCCGGACA<br>AAGCGTTCCAGGACAAGCTGTATCCGTTTACCTGGGATGCCGTACGTTACAACGGCAAGCTGATTGCTTACC<br>CGATCGCTGTTGAAGCGTTATCGCTGATTTATAACAAAGATCTGCTGCCGAACCCGCCAAAAACCTGGGAAG<br>AGATCCCGGCGCTGGATAAAGAACTGAAAGCGAAAGGTAAGAGCGCGCTGATGTTCAACCTGCAAGAACCG<br>TACTTCACCTGGCCGCTGATTGCTGCTGACGGGGGTTATGCGTTCAAGTATGAAAACGGCAAGTACGACATT<br>AAAGACGTGGGCGTGGATAACGCTGGCGCGAAAGCGGGTCTGACCTTCCTGGTTGACCTGATTAAAAACAA<br>ACACATGAATGCAGACACCGATTACTCCATCGCAGAAGCTGCCTTTAATAAAGGCGAAACAGCGATGACCAT<br>CAACGGCCCGTGGGCATGGTCCAACATCGACACCAGCAAAGTGAATTATGGTGTAACGGTACTGCCGACCTT<br>CAAGGGTCAACCATCCAAACCGTTCGTTGGCGTGCTGAGCGCAGGTATTAACGCCGCCAGTCCGAACAAAG<br>AGCTGGCAAAAGAGTTCCTCGAAAACTATCTGCTGACTGATGAAGGTCTGGAAGCGGTTAATAAAGACAAAC<br>CGCTGGGTGCCGTAGCGCTGAAGTCTTACGAGGAAGAGTTGGCGAAAGATCCACGTATTGCCGCCACCATG<br>GAAAACGCCCAGAAAGGTGAAATCATGCCGAACATCCCGCAGATGTCCGCTTTCTGGTATGCCGTGCGTACT<br>GCGGTGATCAACGCCGCCAGCGGTCGTCAGACTGTCGATGAAGCCCTGAAAGACGCGCAGACTAATTCGAG<br>CTCGAACAACAACAACAATAACAATAACAACAACCTCGGGcTGCAGgCTTCAAGCGAAAATCTGTATTTCCAAT<br>GGAGCGCGGACAATCCGACAAACACTGATGTAAACACCCATTATTGGCTTTTCAAGCAGGCTGAGAAAATTTT<br>AGCAAAAGACGTGAATCATATGCGCGCAAACCTGATGAACGAACTTAAGAAGTTTGATAAACAAATAGCCCAG<br>GGAATTTACGATGCCGACCACAAAAACCCCTATTATGACACTAGCACATTTCTGTCCCACTTCTATAACCCAG<br>ATCGCGATAACACGTACTTGCCTGGATTCGCAAATGCAAAGATTACGGGCGCTAAATATTTCAATCAAAGTGT<br>TACGGACTATCGAGAAGGTAAATTCGATACAGCCTTCTACAAATTAGGTCTGGCCATCCACTATTACACAGAT<br>ATTAGTCAACCTATGCACGCGAATAACTTCACGGCAATTAGCTATCCTCCGGGATATCATTGTGCATACGAAA<br>ACTACGTTGATACCATTAAACACAACTACCAGGCTACAGAAGATATGGTTGCCAAACGGTTTTGCAGCGACGA<br>CGTTAAAGATTGGTTATATGAAAACGCCAAACGTGCTAAAGCAGATTATCCGAAGATAGTAAACGCGAAAACA<br>AAAAAAAGCTACCTCGTGGGAAACTCTGAATGGAAAAAGGATACGGTCGAACCTACAGGAGCTCGCTTACGT<br>GATTCACAGCAGACGTTAGCCGGGTTCTTAGAATTTTGGTCTAAAAAGACAAATGAAAGCGGTGGTTCTGGT<br>GGATCGGGATCCAGTACCAAGAAGTCGCCGGAAGAATTAAAGCGTATTTTCGAGAAATATGCCGCAAAAGAG<br>GGCGATCCGGAcCAGCTTTCCAAGGACGAGCTTAAGTTGTTAATTCAAGCTGAgTTTCCTTCTTTAGGTGGTT<br>CTGGTGGTTCCGAAGTGCAACTGGTTGAATCAGGAGGCGGTTTAGTGCAGGCTGGAGGGAGTCTGCGTCTG<br>TCTTGTGCCGCATCTGGCCGTACATTTAGCGATTATGTGATGGGCTGGTTtCGCCAAGCACCAGGGAAAGAA<br>CGCACATTCGTGGCGCGGATCGGTTGGAGTGGTGACCTGACGTACTATGCAGACAGTGTTAAAGGCAGATTT<br>ACTATCTCTCGCGACAACGCAAAGAATACGGTGTACTTGCAAATGAACTCATTAAAACCGGAGGACACTGCC<br>ATTTATTACTGTGCAGCAGATGAACGGTGGGGCACAGGTGGTAAATTCGACTATTGGGGACAAGGAACACAA<br>GTTACAGTAAGCGGCGGTAGCGGTGGCAGCTCCACGAAAAAAAGCCCAGAAGAACTTAAACGCATATTCGAA<br>AAGTATGCTGCCAAGGAAGGCGACCCGGATCAGCTGTCTAAAGATGAGCTGAAACTTCTCATTCAGGCCGAG<br>TTCCCTAGCTTAGCTAGCGCTTGGTCGCACCCGCAGTTTGAGAAATaa	97,952	164,810
PLC-Cn-NB$_{CD9}$-Cn	-	-	-	Sequence corresponding to active PLC-Cn-NBCD9-Cn (MBP cleaved off by TEV protease)	TGGAGCGCGGACAATCCGACAAACACTGATGTAAACACCCATTATTGGCTTTTCAAGCAGGCTGAGAAAATTT<br>TAGCAAAAGACGTGAATCATATGCGCGCAAACCTGATGAACGAACTTAAGAAGTTTGATAAACAAATAGCCCA<br>GGGAATTTACGATGCCGACCACAAAAACCCCTATTATGACACTAGCACATTTCTGTCCCACTTCTATAACCCA<br>GATCGCGATAACACGTACTTGCCTGGATTCGCAAATGCAAAGATTACGGGCGCTAAATATTTCAATCAAAGTG<br>TTACGGACTATCGAGAAGGTAAATTCGATACAGCCTTCTACAAATTAGGTCTGGCCATCCACTATTACACAGA<br>TATTAGTCAACCTATGCACGCGAATAACTTCACGGCAATTAGCTATCCTCCGGGATATCATTGTGCATACGAA<br>AACTACGTTGATACCATTAAACACAACTACCAGGCTACAGAAGATATGGTTGCCAAACGGTTTTGCAGCGACG<br>ACGTTAAAGATTGGTTATATGAAAACGCCAAACGTGCTAAAGCAGATTATCCGAAGATAGTAAACGCGAAAAC<br>AAAAAAAAGCTACCTCGTGGGAAACTCTGAATGGAAAAAGGATACGGTCGAACCTACAGGAGCTCGCTTACG<br>TGATTCACAGCAGACGTTAGCCGGGTTCTTAGAATTTTGGTCTAAAAAGACAAATGAAAGCGGTGGTTCTGGT<br>GGATCGGGATCCAGTACCAAGAAGTCGCCGGAAGAATTAAAGCGTATTTTCGAGAAATATGCCGCAAAAGAG<br>GGCGATCCGGAcCAGCTTTCCAAGGACGAGCTTAAGTTGTTAATTCAAGCTGAgTTTCCTTCTTTAGGTGGTT<br>CTGGTGGTTCCGAAGTGCAACTGGTTGAATCAGGAGGCGGTTTAGTGCAGGCTGGAGGGAGTCTGCGTCTG<br>TCTTGTGCCGCATCTGGCCGTACATTTAGCGATTATGTGATGGGCTGGTTtCGCCAAGCACCAGGGAAAGAA<br>CGCACATTCGTGGCGCGGATCGGTTGGAGTGGTGACCTGACGTACTATGCAGACAGTGTTAAAGGCAGATTT<br>ACTATCTCTCGCGACAACGCAAAGAATACGGTGTACTTGCAAATGAACTCATTAAAACCGGAGGACACTGCC<br>ATTTATTACTGTGCAGCAGATGAACGGTGGGGCACAGGTGGTAAATTCGACTATTGGGGACAAGGAACACAA<br>GTTACAGTAAGCGGCGGTAGCGGTGGCAGCTCCACGAAAAAAAGCCCAGAAGAACTTAAACGCATATTCGAA<br>AAGTATGCTGCCAAGGAAGGCGACCCGGATCAGCTGTCTAAAGATGAGCTGAAACTTCTCATTCAGGCCGAG<br>TTCCCTAGCTTAGCTAGCGCTTGGTCGCACCCGCAGTTTGAGAAATaa	53,191	96,970
pET_MBP-PLC-Cn-NB$_{ALFA}$-Cn	pET_MBP-PLC-TFAM (BamHI + XhoI)	Cn-NB$_{ALFA}$-Cn (BamHI + XhoI)	-	Insert purchased from Twist Bioscience	ATGGGTAGCAGCCATCATCATCATCACCACCAAGCTTcgatGAAGATCGAAGAAGGTAAACTGGTAATCTGGAT<br>TAACGGCGATAAAGGCTATAACGGTCTCGCTGAAGTCGGTAAGAAATTCGAGAAAGATACCGGAATTAAAGT<br>CACCGTTGAGCATCCGGATAAACTGGAAGAGAAATTCCCACAGGTTGCGGCAACTGGCGATGGCCCTGACA<br>TTATCTTCTGGGCACACGACCGCTTTGGTGGCTACGCTCAATCTGGCCTGTTGGCTGAAATCACCCCGGACA<br>AAGCGTTCCAGGACAAGCTGTATCCGTTTACCTGGGATGCCGTACGTTACAACGGCAAGCTGATTGCTTACC<br>CGATCGCTGTTGAAGCGTTATCGCTGATTTATAACAAAGATCTGCTGCCGAACCCGCCAAAAACCTGGGAAG<br>AGATCCCGGCGCTGGATAAAGAACTGAAAGCGAAAGGTAAGAGCGCGCTGATGTTCAACCTGCAAGAACCG<br>TACTTCACCTGGCCGCTGATTGCTGCTGACGGGGGTTATGCGTTCAAGTATGAAAACGGCAAGTACGACATT<br>AAAGACGTGGGCGTGGATAACGCTGGCGCGAAAGCGGGTCTGACCTTCCTGGTTGACCTGATTAAAAACAA<br>ACACATGAATGCAGACACCGATTACTCCATCGCAGAAGCTGCCTTTAATAAAGGCGAAACAGCGATGACCAT<br>CAACGGCCCGTGGGCATGGTCCAACATCGACACCAGCAAAGTGAATTATGGTGTAACGGTACTGCCGACCTT<br>CAAGGGTCAACCATCCAAACCGTTCGTTGGCGTGCTGAGCGCAGGTATTAACGCCGCCAGTCCGAACAAAG<br>AGCTGGCAAAAGAGTTCCTCGAAAACTATCTGCTGACTGATGAAGGTCTGGAAGCGGTTAATAAAGACAAAC<br>CGCTGGGTGCCGTAGCGCTGAAGTCTTACGAGGAAGAGTTGGCGAAAGATCCACGTATTGCCGCCACCATG	98,209	150,830

					GAAAACGCCCAGAAAGGTGAAATCATGCCGAACATCCCGCAGATGTCCGCTTTCTGGTATGCCGTGCGTACT<br>GCGGTGATCAACGCCGCCAGCGGTCGTCAGACTGTCGATGAAGCCCTGAAAGACGCGCAGACTAATTCGAG<br>CTCGAACAACAACAACAATAACAATAACAACAACCTCGGGctgcaGgCTTCAAGCGAAAATCTGTATTTCCAATG<br>GAGCGCGGACAATCCGACAAACACTGATGTAAACACCCATTATTGGCTTTTCAAGCAGGCTGAGAAAATTTTA<br>GCAAAAGACGTGAATCATATGCGCGCAAACCTGATGAACGAACTTAAGAAGTTTGATAAACAAATAGCCCAG<br>GGAATTTACGATGCCGACCACAAAAACCCCTATTATGACACTAGCACATTTCTGTCCCACTTCTATAACCCAG<br>ATCGCGATAACACGTACTTGCCTGGATTCGCAAATGCAAAGATTACGGGCGCTAAATATTTCAATCAAAGTGT<br>TACGGACTATCGAGAAGGTAAATTCGATACAGCCTTCTACAAATTAGGTCTGGCCATCCACTATTACACAGAT<br>ATTAGTCAACCTATGCACGCGAATAACTTCACGGCAATTAGCTATCCTCCGGGATATCATTGTGCATACGAAA<br>ACTACGTTGATACCATTAAACACAACTACCAGGCTACAGAAGATATGGTTGCCAAACGGTTTTGCAGCGACGA<br>CGTTAAAGATTGGTTATATGAAAACGCCAAACGTGCTAAAGCAGATTATCCGAAGATAGTAAACGCGAAAACA<br>AAAAAAAGCTACCTCGTGGGAAACTCTGAATGGAAAAAGGATACGGTCGAACCTACAGGAGCTCGCTTACGT<br>GATTCACAGCAGACGTTAGCCGGGTTCTTAGAATTTTGGTCTAAAAAGACAAATGAAAGCGGTGGTTCTGGT<br>GGATCGGGATCCAGTACCAAGAAGTCGCCGGAAGAATTAAAGCGTATTTTCGAGAAATATGCCGCAAAAGAG<br>GGCGATCCGGAcCAGCTTTCCAAGGACGAGCTTAAGTTGTTAATTCAAGCTGAgTTTCCTTCTTTAGGTGGTT<br>CTGGTGGTTCCGAGGTCCAGTTACAGGAGAGCGGAGGTGGACTCGTGCAGCCGGGGGGGAGTCTGCGCTT<br>GTCTTGCACCGCCTCTGGTGTTACCATAAGTGCGCTGAATGCGATGGCAATGGGCTGGTATCGCCAAGCCC<br>CTGGAGAACGCAGAGTGATGGTGGCCGCCGTGAGCGAACGCGGCAATGCAATGTATCGGGAATCCGTCCA<br>GGGCAGGTTTACTGTCACACGCGACTTTACGAATAAAATGGTGTCCTTACAAATGGACAATCTTAAGCCCGAG<br>GATACAGCGGTTTATTATTGTCATGTTCTGGAAGACAGAGTTGATAGCTTTCACGATTATTGGGGTCAGGGTA<br>CACAGGTCACCGTTAGCTCAGGCGGTAGCGGTGGCAGCTCCACGAAAAAAAGCCCAGAAGAACTTAAACGC<br>ATATTCGAAAAGTATGCTGCCAAGGAAGGCGACCCGGATCAGCTGTCTAAAGATGAGCTGAAACTTCTCATT<br>CAGGCCGAGTTCCCTAGCTTAGCTAGCGCTTGGTCGCACCCGCAGTTTGAGAAATAA		
PLC-Cn-NB$_{ALFA}$-Cn	-	-	-	Sequence corresponding to active PLC-Cn-NB$_{ALFA}$-Cn (MBP cleaved off by TEV protease)	TGGAGCGCGGACAATCCGACAAACACTGATGTAAACACCCATTATTGGCTTTTCAAGCAGGCTGAGAAAATTT<br>TAGCAAAAGACGTGAATCATATGCGCGCAAACCTGATGAACGAACTTAAGAAGTTTGATAAACAAATAGCCCA<br>GGGAATTTACGATGCCGACCACAAAAACCCCTATTATGACACTAGCACATTTCTGTCCCACTTCTATAACCCA<br>GATCGCGATAACACGTACTTGCCTGGATTCGCAAATGCAAAGATTACGGGCGCTAAATATTTCAATCAAAGTG<br>TTACGGACTATCGAGAAGGTAAATTCGATACAGCCTTCTACAAATTAGGTCTGGCCATCCACTATTACACAGA<br>TATTAGTCAACCTATGCACGCGAATAACTTCACGGCAATTAGCTATCCTCCGGGATATCATTGTGCATACGAA<br>AACTACGTTGATACCATTAAACACAACTACCAGGCTACAGAAGATATGGTTGCCAAACGGTTTTGCAGCGACG<br>ACGTTAAAGATTGGTTATATGAAAACGCCAAACGTGCTAAAGCAGATTATCCGAAGATAGTAAACGCGAAAAC<br>AAAAAAAAGCTACCTCGTGGGAAACTCTGAATGGAAAAAGGATACGGTCGAACCTACAGGAGCTCGCTTACG<br>TGATTCACAGCAGACGTTAGCCGGGTTCTTAGAATTTTGGTCTAAAAAGACAAATGAAAGCGGTGGTTCTGGT<br>GGATCGGGATCCAGTACCAAGAAGTCGCCGGAAGAATTAAAGCGTATTTTCGAGAAATATGCCGCAAAAGAG<br>GGCGATCCGGAcCAGCTTTCCAAGGACGAGCTTAAGTTGTTAATTCAAGCTGAgTTTCCTTCTTTAGGTGGTT<br>CTGGTGGTTCCGAGGTCCAGTTACAGGAGAGCGGAGGTGGACTCGTGCAGCCGGGGGGGAGTCTGCGCTT<br>GTCTTGCACCGCCTCTGGTGTTACCATAAGTGCGCTGAATGCGATGGCAATGGGCTGGTATCGCCAAGCCC<br>CTGGAGAACGCAGAGTGATGGTGGCCGCCGTGAGCGAACGCGGCAATGCAATGTATCGGGAATCCGTCCA<br>GGGCAGGTTTACTGTCACACGCGACTTTACGAATAAAATGGTGTCCTTACAAATGGACAATCTTAAGCCCGAG<br>GATACAGCGGTTTATTATTGTCATGTTCTGGAAGACAGAGTTGATAGCTTTCACGATTATTGGGGTCAGGGTA<br>CACAGGTCACCGTTAGCTCAGGCGGTAGCGGTGGCAGCTCCACGAAAAAAAGCCCAGAAGAACTTAAACGC<br>ATATTCGAAAAGTATGCTGCCAAGGAAGGCGACCCGGATCAGCTGTCTAAAGATGAGCTGAAACTTCTCATT<br>CAGGCCGAGTTCCCTAGCTTAGCTAGCGCTTGGTCGCACCCGCAGTTTGAGAAATAA	53,448	82,990

pET_MBP-PLC-NB$_{CD9}$-TFAM	pET_PLC-Cn-NBCD9-Cn (BamHI + XhoI)	NB$_{CD9}$ (BamHI + EcoRI)	TFAM (EcoRI + XhoI)	Inserts purchased from Twist Bioscience	ATGGGTAGCAGCCATCATCATCATCACCACCAAGCTTcgatGAAGATCGAAGAAGGTAAACTGGTAATCTGGAT<br>TAACGGCGATAAAGGCTATAACGGTCTCGCTGAAGTCGGTAAGAAATTCGAGAAAGATACCGGAATTAAAGT<br>CACCGTTGAGCATCCGGATAAACTGGAAGAGAAATTCCCACAGGTTGCGGCAACTGGCGATGGCCCTGACA<br>TTATCTTCTGGGCACACGACCGCTTTGGTGGCTACGCTCAATCTGGCCTGTTGGCTGAAATCACCCCGGACA<br>AAGCGTTCCAGGACAAGCTGTATCCGTTTACCTGGGATGCCGTACGTTACAACGGCAAGCTGATTGCTTACC<br>CGATCGCTGTTGAAGCGTTATCGCTGATTTATAACAAAGATCTGCTGCCGAACCCGCCAAAAACCTGGGAAG<br>AGATCCCGGCGCTGGATAAAGAACTGAAAGCGAAAGGTAAGAGCGCGCTGATGTTCAACCTGCAAGAACCG<br>TACTTCACCTGGCCGCTGATTGCTGCTGACGGGGGTTATGCGTTCAAGTATGAAAACGGCAAGTACGACATT<br>AAAGACGTGGGCGTGGATAACGCTGGCGCGAAAGCGGGTCTGACCTTCCTGGTTGACCTGATTAAAAACAA<br>ACACATGAATGCAGACACCGATTACTCCATCGCAGAAGCTGCCTTTAATAAAGGCGAAACAGCGATGACCAT<br>CAACGGCCCGTGGGCATGGTCCAACATCGACACCAGCAAAGTGAATTATGGTGTAACGGTACTGCCGACCTT<br>CAAGGGTCAACCATCCAAACCGTTCGTTGGCGTGCTGAGCGCAGGTATTAACGCCGCCAGTCCGAACAAAG<br>AGCTGGCAAAAGAGTTCCTCGAAAACTATCTGCTGACTGATGAAGGTCTGGAAGCGGTTAATAAAGACAAAC<br>CGCTGGGTGCCGTAGCGCTGAAGTCTTACGAGGAAGAGTTGGCGAAAGATCCACGTATTGCCGCCACCATG<br>GAAAACGCCCAGAAAGGTGAAATCATGCCGAACATCCCGCAGATGTCCGCTTTCTGGTATGCCGTGCGTACT<br>GCGGTGATCAACGCCGCCAGCGGTCGTCAGACTGTCGATGAAGCCCTGAAAGACGCGCAGACTAATTCGAG<br>CTCGAACAACAACAACAATAACAATAACAACAACCTCGGGctgcaGgCTTCAAGCGAAAATCTGTATTTCCAATG<br>GAGCGCGGACAATCCGACAAACACTGATGTAAACACCCATTATTGGCTTTTCAAGCAGGCTGAGAAAATTTTA<br>GCAAAAGACGTGAATCATATGCGCGCAAACCTGATGAACGAACTTAAGAAGTTTGATAAACAAATAGCCCAG<br>GGAATTTACGATGCCGACCACAAAAACCCCTATTATGACACTAGCACATTTCTGTCCCACTTCTATAACCCAG<br>ATCGCGATAACACGTACTTGCCTGGATTCGCAAATGCAAAGATTACGGGCGCTAAATATTTCAATCAAAGTGT<br>TACGGACTATCGAGAAGGTAAATTCGATACAGCCTTCTACAAATTAGGTCTGGCCATCCACTATTACACAGAT<br>ATTAGTCAACCTATGCACGCGAATAACTTCACGGCAATTAGCTATCCTCCGGGATATCATTGTGCATACGAAA<br>ACTACGTTGATACCATTAAACACAACTACCAGGCTACAGAAGATATGGTTGCCAAACGGTTTTGCAGCGACGA<br>CGTTAAAGATTGGTTATATGAAAACGCCAAACGTGCTAAAGCAGATTATCCGAAGATAGTAAACGCGAAAACA<br>AAAAAAAGCTACCTCGTGGGAAACTCTGAATGGAAAAAGGATACGGTCGAACCTACAGGAGCTCGCTTACGT<br>GATTCACAGCAGACGTTAGCCGGGTTCTTAGAATTTTGGTCTAAAAAGACAAATGAAAGCGGTGGTTCTGGT<br>GGATCGGGATCCGAAGTGCAACTGGTTGAATCAGGAGGCGGTTTAGTGCAGGCTGGAGGGAGTCTGCGTCT<br>GTCTTGTGCCGCATCTGGCCGTACATTTAGCGATTATGTGATGGGCTGGTTtCGCCAAGCACCAGGGAAAGA<br>ACGCACATTCGTGGCGCGGATCGGTTGGAGTGGTGACCTGACGTACTATGCAGACAGTGTTAAAGGCAGAT<br>TTACTATCTCTCGCGACAACGCAAAGAATACGGTGTACTTGCAAATGAACTCATTAAAACCGGAGGACACTGC<br>CATTTATTACTGTGCAGCAGATGAACGGTGGGGCACAGGTGGTAAATTCGACTATTGGGGACAAGGAACACA<br>AGTTACAGTAAGCGGGAATTCtggtggcggtagcggtggcATGTCATCTGTCTTGGCAAGTTGTCCAAAGAAACCTGT<br>AAGTTCTTACCTTCGATTTTCTAAAGAACAACTACCCATATTTAAAGCTCAGAACCCAGATGCAAAAACTACAG<br>AACTAATTAGAAGAATTGCCCAGCGTTGGAGGGAACTTCCTGATTCAAAGAAAAAAATATATCAAGATGCTTAT<br>AGGtgcGAGTGGCAatgtTATAAAGAAGAGATAAGCAGATTTAAAGAgCAGtTgACTCCAAGTCAGATTATGTCTTT<br>GGAAAAAGAAATCATGGACAAACATTTAAAAAGGAAAGCTATGACAAAAAAAAAAGAGTTAACACTGCTTGGA<br>AAACCAAAAAGACCTCGTTCAGCTTATAACGTTTATGTAGCTGAAAGATTCCAAGAAGCTAAGGGTGATTCAC<br>CGCAGGAAAAGCTGAAGACTGTAAAGGAAAACTGGAAAAATCTGTCTGACTCTGAAAAGGAATTATATATTCA<br>GCATGCTAAAGAGGACGAAACTCGTTATCATAATGAAATGAAGTCTTGGGAAGAACAAATGATTGAAGTTGGA<br>CGAAAGGATCTTCTACGTCGCACAATAAAGAAACAACGAAAATATGGTGCTGAGGAGTGTTaa	111,451	191,990
PLC-NB$_{CD9}$-TFAM	-	-	-	Sequence corresponding to active PLC-NB$_{CD9}$-TFAM (MBP cleaved off by TEV protease)	TGGAGCGCGGACAATCCGACAAACACTGATGTAAACACCCATTATTGGCTTTTCAAGCAGGCTGAGAAAATTT<br>TAGCAAAAGACGTGAATCATATGCGCGCAAACCTGATGAACGAACTTAAGAAGTTTGATAAACAAATAGCCCA<br>GGGAATTTACGATGCCGACCACAAAAACCCCTATTATGACACTAGCACATTTCTGTCCCACTTCTATAACCCA<br>GATCGCGATAACACGTACTTGCCTGGATTCGCAAATGCAAAGATTACGGGCGCTAAATATTTCAATCAAAGTG<br>TTACGGACTATCGAGAAGGTAAATTCGATACAGCCTTCTACAAATTAGGTCTGGCCATCCACTATTACACAGA<br>TATTAGTCAACCTATGCACGCGAATAACTTCACGGCAATTAGCTATCCTCCGGGATATCATTGTGCATACGAA<br>AACTACGTTGATACCATTAAACACAACTACCAGGCTACAGAAGATATGGTTGCCAAACGGTTTTGCAGCGACG<br>ACGTTAAAGATTGGTTATATGAAAACGCCAAACGTGCTAAAGCAGATTATCCGAAGATAGTAAACGCGAAAAC<br>AAAAAAAAGCTACCTCGTGGGAAACTCTGAATGGAAAAAGGATACGGTCGAACCTACAGGAGCTCGCTTACG<br>TGATTCACAGCAGACGTTAGCCGGGTTCTTAGAATTTTGGTCTAAAAAGACAAATGAAAGCGGTGGTTCTGGT<br>GGATCGGGATCCGAAGTGCAACTGGTTGAATCAGGAGGCGGTTTAGTGCAGGCTGGAGGGAGTCTGCGTCT<br>GTCTTGTGCCGCATCTGGCCGTACATTTAGCGATTATGTGATGGGCTGGTTtCGCCAAGCACCAGGGAAAGA<br>ACGCACATTCGTGGCGCGGATCGGTTGGAGTGGTGACCTGACGTACTATGCAGACAGTGTTAAAGGCAGAT<br>TTACTATCTCTCGCGACAACGCAAAGAATACGGTGTACTTGCAAATGAACTCATTAAAACCGGAGGACACTGC<br>CATTTATTACTGTGCAGCAGATGAACGGTGGGGCACAGGTGGTAAATTCGACTATTGGGGACAAGGAACACA<br>AGTTACAGTAAGCGGGAATTCtggtggcggtagcggtggcATGTCATCTGTCTTGGCAAGTTGTCCAAAGAAACCTGT<br>AAGTTCTTACCTTCGATTTTCTAAAGAACAACTACCCATATTTAAAGCTCAGAACCCAGATGCAAAAACTACAG<br>AACTAATTAGAAGAATTGCCCAGCGTTGGAGGGAACTTCCTGATTCAAAGAAAAAAATATATCAAGATGCTTAT<br>AGGtgcGAGTGGCAatgtTATAAAGAAGAGATAAGCAGATTTAAAGAgCAGtTgACTCCAAGTCAGATTATGTCTTT<br>GGAAAAAGAAATCATGGACAAACATTTAAAAAGGAAAGCTATGACAAAAAAAAAAGAGTTAACACTGCTTGGA<br>AAACCAAAAAGACCTCGTTCAGCTTATAACGTTTATGTAGCTGAAAGATTCCAAGAAGCTAAGGGTGATTCAC<br>CGCAGGAAAAGCTGAAGACTGTAAAGGAAAACTGGAAAAATCTGTCTGACTCTGAAAAGGAATTATATATTCA<br>GCATGCTAAAGAGGACGAAACTCGTTATCATAATGAAATGAAGTCTTGGGAAGAACAAATGATTGAAGTTGGA<br>CGAAAGGATCTTCTACGTCGCACAATAAAGAAACAACGAAAATATGGTGCTGAGGAGTGTTaa	66,689	124,150

pET_TFAM-VRK1-Cc	pET_TFAM-Cc (BamHI + XhoI)	VRK1 (BamHI + NheI)	Cc (NheI + XhoI)	VRK1 insert generated by PCR (forward primer: 5’-GGTGGATCCATGCCC-3’, reverse primer: 5’-CATGCTAGCTACTTTCCGTTTCTTCTTCG-3’, template: pET_TFAM-VRK1);<br>Cc insert generated by PCR (forward primer: 5’-GTAGCTAGCGGCGGTTCTCTTAAAGGTC-3’, reverse primer: 5’-GTGCTCGAGTTACTGTGAAATC-3’, template: pET_TFAM-Cc)	ATGGGGAGtTCACACCATCATCACCACCAcGGATCTGGTAGTATGAGTTCAGTGCTGGCTAGCTGTCCGAAAA<br>AACCAGTCTCTTCATATCTGCGTTTTTCAAAAGAGCAGTTGCCAATCTTTAAGGCCCAAAATCCAGATGCGAA<br>AACAACTGAGCTGATTAGACGCATAGCGCAACGGTGGAGAGAACTGCCGGACTCCAAGAAGAAGATTTATCA<br>GGACGCGTATCGCTGTGAGTGGCAATGCTATAAAGAAGAAATATCGCGTTTCAAAGAACAGCTGACCCCTAG<br>TCAGATTATGTCCCTTGAGAAAGAAATCATGGATAAACACCTGAAACGAAAAGCAATGACCAAGAAAAAAGAA<br>TTAACCTTACTGGGAAAACCAAAGCGGCCGCGCAGTGCATACAATGTTTATGTGGCTGAACGGTTTCAAGAG<br>GCAAAAGGCGATTCTCCTCAGGAGAAACTGAAAACGGTTAAAGAAAATTGGAAGAACCTCTCCGATTCAGAG<br>AAGGAACTGTATATCCAGCACGCTAAAGAGGATGAAACAAGATATCATAACGAAATGAAATCCTGGGAGGAG<br>CAGATGATTGAGGTAGGTCGGAAAGAcCTTCTACGTCGCACTATTAAGAAACAGCGCAAATACGGTGCTGAA<br>GAATGCAGTGGGGGTAGCTCCGGCCGTGGATCCATGCCCCGTGTGAAGGCGGCGCAGGCTGGACGGCAGT<br>CTTCAGCGAAGcgtCACCTCGCGGAGCAGTTTGCAGTTGGAGAAATTATCACTGATATGGCTAAAAAGGAGTG<br>GAAAGTGGGACTGCCTATTGGACAAGGTGGATTTGGTTGTATCTATCTGGCCGACATGAACTCTTCGGAATC<br>TGTGGGCTCAGATGCTCCCTGTGTAGTCAAAGTAGAACCTTCAGATAATGGGCCGCTGTTTACTGAACTGAA<br>ATTTTATCAAAGGGCTGCTAAACCTGAACAGATACAAAAATGGATACGGACTCGGAAATTGAAATATCTCGGC<br>GTACCAAAATATTGGGGTAGCGGACTTCATGATAAAAATGGGAAATCGTATCGTTTTATGATAATGGACCGGT<br>TCGGCTCGGACTTACAAAAAATTTACGAGGCGAACGCCAAACGGTTTAGCCGCAAGACTGTATTACAGCTGA<br>GCTTGCGCATTCTgGATATTCTTGAgTATATcCACGAACATGAATATGTTCATGGTGATATTAAGGCAagcAATTT<br>ATTATTGAACTATAAGAACCCGGATCAGGTATATTTGGTGGATTACGGTCTGGCATACCGCTACTGCCCGGA<br>GGGAGTACACAAAGAGTATAAGGAAGACCCAAAACGGTGTCATGAcGGAACCATCGAATTTACCTCGATAGA<br>CGCACATAACGGTGTCGCGCCCTCACGTCGTGGAGAcCTGGAAATCCTGGGATATTGCATGATTCAGTGGCT<br>GACGGGACACCTTCCgTGGGAGGATAATCTGAAAGATCCTAAGTATGTGCGAGACAGTAAGATTAGATACAG<br>GGAAAATATAGCCAGCCTGATGGATAAATGCTTTCCaGAAAAGAACAAACCGGGAGAAATCGCTAAATATATG<br>GAGACTGTCAAACTTTTGGATTACACCGAGAAACCGCTGTATGAAAACCTCCGCGATATtTTACTACAGGGCC<br>TGAAAGCCATTGGCAGTAAAGATGATGGCAAGTTAGAcCTGTCAGTGGTTGAAAACGGGGGTCTTAAAGCAA<br>AGACAATTACGAAAAAACGAAAGAAAGAGATTGAAGAATCAAAAGAACCAGGCGTTGAAGATACTGAATGGA<br>GCAATACACAGACAGAAGAGGCTATCCAGACGCGTTCCAGAACCCGCAAACGTGTTCAGAAGAGCGGTCCG<br>AAGAAGAAACGGAAAGTAGCTAGCGGCGGTTCTCTTAAAGGTCCGAATACTTTAGAtGACCTATTTCAAGAAC<br>TGGAcAAAAACGGTGACGGGGAGGTCTCATTTGAGGAGTTTCAGGTGCTGGTGAAgAAGATtTCACAGTAA	77,663	98,210
pEGFP	-	-	-	Entire plasmid DNA sequence is specified. Plasmid was used for transfections	CTGCTCCCTGCTTGTGTGTTGGAGGTCGCTGAGTAGTGCGCGAGCAAAATTTAAGCTACAACAAGGCAAGGC<br>TTGACCGACAATTGCATGAAGAATCTGCTTAGGGTTAGGCGTTTTGCGCTGCTTCGCGATGTACGGGCCAGA<br>TATACGCGTTGACATTGATTATTGACTAGTTATTAATAGTAATCAATTACGGGGTCATTAGTTCATAGCCCATA<br>TATGGAGTTCCGCGTTACATAACTTACGGTAAATGGCCCGCCTGGCTGACCGCCCAACGACCCCCGCCCATT<br>GACGTCAATAATGACGTATGTTCCCATAGTAACGCCAATAGGGACTTTCCATTGACGTCAATGGGTGGAGTAT<br>TTACGGTAAACTGCCCACTTGGCAGTACATCAAGTGTATCATATGCCAAGTACGCCCCCTATTGACGTCAATG<br>ACGGTAAATGGCCCGCCTGGCATTATGCCCAGTACATGACCTTATGGGACTTTCCTACTTGGCAGTACATCTA<br>CGTATTAGTCATCGCTATTACCATGGTGATGCGGTTTTGGCAGTACATCAATGGGCGTGGATAGCGGTTTGA<br>CTCACGGGGATTTCCAAGTCTCCACCCCATTGACGTCAATGGGAGTTTGTTTTGGCACCAAAATCAACGGGA<br>CTTTCCAAAATGTCGTAACAACTCCGCCCCATTGACGCAAATGGGCGGTAGGCGTGTACGGTGGGAGGTCTA<br>TATAAGCAGAGCTCTCTGGCTAACTAGAGAACCCACTGCTTACTGGCTTATCGAAATTAATACGACTCACTAT<br>AGGGAGACCCAAGCTGGCTAGCGTTTAAACTTAAGCTTGGTACCGAGCTCGGATCCACCGGTCGCCACCAT<br>GGTGAGCAAGGGCGAGGAGCTGTTCACCGGGGTGGTGCCCATCCTGGTCGAGCTGGACGGCGACGTAAAC<br>GGCCACAAGTTCAGCGTGTCCGGCGAGGGCGAGGGCGATGCCACCTACGGCAAGCTGACCCTGAAGTTCA<br>TCTGCACCACCGGCAAGCTGCCCGTGCCCTGGCCCACCCTCGTGACCACCCTGACCTACGGCGTGCAGTG<br>CTTCAGCCGCTACCCCGACCACATGAAGCAGCACGACTTCTTCAAGTCCGCCATGCCCGAAGGCTACGTCC<br>AGGAGCGCACCATCTTCTTCAAGGACGACGGCAACTACAAGACCCGCGCCGAGGTGAAGTTCGAGGGCGAC<br>ACCCTGGTGAACCGCATCGAGCTGAAGGGCATCGACTTCAAGGAGGACGGCAACATCCTGGGGCACAAGCT<br>GGAGTACAACTACAACAGCCACAACGTCTATATCATGGCCGACAAGCAGAAGAACGGCATCAAGGTGAACTT<br>CAAGATCCGCCACAACATCGAGGACGGCAGCGTGCAGCTCGCCGACCACTACCAGCAGAACACCCCCATCG<br>GCGACGGCCCCGTGCTGCTGCCCGACAACCACTACCTGAGCACCCAGTCCGCCCTGAGCAAAGACCCCAA<br>CGAGAAGCGCGATCACATGGTCCTGCTGGAGTTCGTGACCGCCGCCGGGATCACTCTCGGCATGGACGAG<br>CTGTACAAGTAAAGCGGCCGCTCGAGTCTAGAGGGCCCGTTTAAACCCGCTGATCAGCCTCGACTGTGCCTT<br>CTAGTTGCCAGCCATCTGTTGTTTGCCCCTCCCCCGTGCCTTCCTTGACCCTGGAAGGTGCCACTCCCACTG<br>TCCTTTCCTAATAAAATGAGGAAATTGCATCGCATTGTCTGAGTAGGTGTCATTCTATTCTGGGGGGTGGGGT<br>GGGGCAGGACAGCAAGGGGGAGGATTGGGAAGACAATAGCAGGCATGCTGGGGATGCGGTGGGCTCTATG<br>GCTTCTGAGGCGGAAAGAACCAGCTGGGGCTCTAGGGGGTATCCCCACGCGCCCTGTAGCGGCGCATTAA<br>GCGCGGCGGGTGTGGTGGTTACGCGCAGCGTGACCGCTACACTTGCCAGCGCCCTAGCGCCCGCTCCTTT<br>CGCTTTCTTCCCTTCCTTTCTCGCCACGTTCGCCGGCTTTCCCCGTCAAGCTCTAAATCGGGGGCTCCCTTTA<br>GGGTTCCGATTTAGTGCTTTACGGCACCTCGACCCCAAAAAACTTGATTAGGGTGATGGTTCACGTAGTGGG<br>CCATCGCCCTGATAGACGGTTTTTCGCCCTTTGACGTTGGAGTCCACGTTCTTTAATAGTGGACTCTTGTTCC<br>AAACTGGAACAACACTCAACCCTATCTCGGTCTATTCTTTTGATTTATAAGGGATTTTGCCGATTTCGGCCTAT<br>TGGTTAAAAAATGAGCTGATTTAACAAAAATTTAACGCGAATTAATTCTGTGGAATGTGTGTCAGTTAGGGTGT<br>GGAAAGTCCCCAGGCTCCCCAGCAGGCAGAAGTATGCAAAGCATGCATCTCAATTAGTCAGCAACCAGGTGT<br>GGAAAGTCCCCAGGCTCCCCAGCAGGCAGAAGTATGCAAAGCATGCATCTCAATTAGTCAGCAACCATAGTC<br>CCGCCCCTAACTCCGCCCATCCCGCCCCTAACTCCGCCCAGTTCCGCCCATTCTCCGCCCCATGGCTGACT<br>AATTTTTTTTATTTATGCAGAGGCCGAGGCCGCCTCTGCCTCTGAGCTATTCCAGAAGTAGTGAGGAGGCTTT<br>TTTGGAGGCCTAGGCTTTTGCAAAAAGCTCCCGGGAGCTTGTATATCCATTTTCGGATCTGATCAAGAGACAG<br>GATGAGGATCGTTTCGCATGATTGAACAAGATGGATTGCACGCAGGTTCTCCGGCCGCTTGGGTGGAGAGG<br>CTATTCGGCTATGACTGGGCACAACAGACAATCGGCTGCTCTGATGCCGCCGTGTTCCGGCTGTCAGCGCA<br>GGGGCGCCCGGTTCTTTTTGTCAAGACCGACCTGTCCGGTGCCCTGAATGAACTGCAGGACGAGGCAGCGC<br>GGCTATCGTGGCTGGCCACGACGGGCGTTCCTTGCGCAGCTGTGCTCGACGTTGTCACTGAAGCGGGAAG<br>GGACTGGCTGCTATTGGGCGAAGTGCCGGGGCAGGATCTCCTGTCATCTCACCTTGCTCCTGCCGAGAAAG<br>TATCCATCATGGCTGATGCAATGCGGCGGCTGCATACGCTTGATCCGGCTACCTGCCCATTCGACCACCAAG<br>CGAAACATCGCATCGAGCGAGCACGTACTCGGATGGAAGCCGGTCTTGTCGATCAGGATGATCTGGACGAA<br>GAGCATCAGGGGCTCGCGCCAGCCGAACTGTTCGCCAGGCTCAAGGCGCGCATGCCCGACGGCGAGGATC<br>TCGTCGTGACCCATGGCGATGCCTGCTTGCCGAATATCATGGTGGAAAATGGCCGCTTTTCTGGATTCATCG<br>ACTGTGGCCGGCTGGGTGTGGCGGACCGCTATCAGGACATAGCGTTGGCTACCCGTGATATTGCTGAAGAG<br>CTTGGCGGCGAATGGGCTGACCGCTTCCTCGTGCTTTACGGTATCGCCGCTCCCGATTCGCAGCGCATCGC<br>CTTCTATCGCCTTCTTGACGAGTTCTTCTGAGCGGGACTCTGGGGTTCGAAATGACCGACCAAGCGACGCCC<br>AACCTGCCATCACGAGATTTCGATTCCACCGCCGCCTTCTATGAAAGGTTGGGCTTCGGAATCGTTTTCCGG<br>GACGCCGGCTGGATGATCCTCCAGCGCGGGGATCTCATGCTGGAGTTCTTCGCCCACCCCAACTTGTTTATT<br>GCAGCTTATAATGGTTACAAATAAAGCAATAGCATCACAAATTTCACAAATAAAGCATTTTTTTCACTGCATTCT<br>AGTTGTGGTTTGTCCAAACTCATCAATGTATCTTATCATGTCTGTATACCGTCGACCTCTAGCTAGAGCTTGG<br>CGTAATCATGGTCATAGCTGTTTCCTGTGTGAAATTGTTATCCGCTCACAATTCCACACAACATACGAGCCGG<br>AAGCATAAAGTGTAAAGCCTGGGGTGCCTAATGAGTGAGCTAACTCACATTAATTGCGTTGCGCTCACTGCC<br>CGCTTTCCAGTCGGGAAACCTGTCGTGCCAGCTGCATTAATGAATCGGCCAACGCGCGGGGAGAGGCGGTT<br>TGCGTATTGGGCGCTCTTCCGCTTCCTCGCTCACTGACTCGCTGCGCTCGGTCGTTCGGCTGCGGCGAGCG<br>GTATCAGCTCACTCAAAGGCGGTAATACGGTTATCCACAGAATCAGGGGATAACGCAGGAAAGAACATGTGA<br>GCAAAAGGCCAGCAAAAGGCCAGGAACCGTAAAAAGGCCGCGTTGCTGGCGTTTTTCCATAGGCTCCGCCC<br>CCCTGACGAGCATCACAAAAATCGACGCTCAAGTCAGAGGTGGCGAAACCCGACAGGACTATAAAGATACCA<br>GGCGTTTCCCCCTGGAAGCTCCCTCGTGCGCTCTCCTGTTCCGACCCTGCCGCTTACCGGATACCTGTCCG<br>CCTTTCTCCCTTCGGGAAGCGTGGCGCTTTCTCATAGCTCACGCTGTAGGTATCTCAGTTCGGTGTAGGTCG<br>TTCGCTCCAAGCTGGGCTGTGTGCACGAACCCCCCGTTCAGCCCGACCGCTGCGCCTTATCCGGTAACTAT<br>CGTCTTGAGTCCAACCCGGTAAGACACGACTTATCGCCACTGGCAGCAGCCACTGGTAACAGGATTAGCAGA	-	-

GCGAGGTATGTAGGCGGTGCTACAGAGTTCTTGAAGTGGTGGCCTAACTACGGCTACACTAGAAGAACAGTA
TTTGGTATCTGCGCTCTGCTGAAGCCAGTTACCTTCGGAAAAAGAGTTGGTAGCTCTTGATCCGGCAAACAAA
CCACCGCTGGTAGCGGTGGTTTTTTTGTTTGCAAGCAGCAGATTACGCGCAGAAAAAAAGGATCTCAAGAAG
ATCCTTTGATCTTTTCTACGGGGTCTGACGCTCAGTGGAACGAAAACTCACGTTAAGGGATTTTGGTCATGAG
ATTATCAAAAAGGATCTTCACCTAGATCCTTTTAAATTAAAAATGAAGTTTTAAATCAATCTAAAGTATATATGA
GTAAACTTGGTCTGACAGTTACCAATGCTTAATCAGTGAGGCACCTATCTCAGCGATCTGTCTATTTCGTTCAT
CCATAGTTGCCTGACTCCCCGTCGTGTAGATAACTACGATACGGGAGGGCTTACCATCTGGCCCCAGTGCTG
CAATGATACCGCGAGACCCACGCTCACCGGCTCCAGATTTATCAGCAATAAACCAGCCAGCCGGAAGGGCC
GAGCGCAGAAGTGGTCCTGCAACTTTATCCGCCTCCATCCAGTCTATTAATTGTTGCCGGGAAGCTAGAGTA
AGTAGTTCGCCAGTTAATAGTTTGCGCAACGTTGTTGCCATTGCTACAGGCATCGTGGTGTCACGCTCGTCG
TTTGGTATGGCTTCATTCAGCTCCGGTTCCCAACGATCAAGGCGAGTTACATGATCCCCCATGTTGTGCAAAA
AAGCGGTTAGCTCCTTCGGTCCTCCGATCGTTGTCAGAAGTAAGTTGGCCGCAGTGTTATCACTCATGGTTAT
GGCAGCACTGCATAATTCTCTTACTGTCATGCCATCCGTAAGATGCTTTTCTGTGACTGGTGAGTACTCAACC
AAGTCATTCTGAGAATAGTGTATGCGGCGACCGAGTTGCTCTTGCCCGGCGTCAATACGGGATAATACCGCG
CCACATAGCAGAACTTTAAAAGTGCTCATCATTGGAAAACGTTCTTCGGGGCGAAAACTCTCAAGGATCTTAC
CGCTGTTGAGATCCAGTTCGATGTAACCCACTCGTGCACCCAACTGATCTTCAGCATCTTTTACTTTCACCAG
CGTTTCTGGGTGAGCAAAAACAGGAAGGCAAAATGCCGCAAAAAAGGGAATAAGGGCGACACGGAAATGTT
GAATACTCATACTCTTCCTTTTTCAATATTATTGAAGCATTTATCAGGGTTATTGTCTCATGAGCGGATACATAT
TTGAATGTATTTAGAAAAATAAACAAATAGGGGTTCCGCGCACATTTCCCCGAAAAGTGCCACCTGACGTCGA
CGGATCGGGAGATCTCCCGATCCCCTATGGTGCACTCTCAGTACAATCTGCTCTGATGCCGCATAGTTAAGC
CAGTAT

Table S2: ImageJ macros used for quantification of endosomal permeation and colocalization of TFAMoplexes with endosomal permeation.

Macro 1. Gal8-mRuby3 dots count batch processing (with free proteins)

```
// Make a list of the files in the input directory
list = getFileList(dir);

// Loop through the list of files
for (i = 0; i < list.length; i++) {
    file = dir + "/" + list[i];

    // Check if the file is not a directory and is a nd2 file --> can be changed for any file type
    if (File.isDirectory(file) == 0 &&
        endsWith(list[i], ".nd2")) {

        // Open the image and print the files in list to check if the correct files are opened
        //run("Bio-Formats Importer", "open=[" + file + "] autoscale display_metadata rois_import=[ROI manager] view=[Hyperstack] stack_order=XYCZT split_channels split_timepoints
split_focal_planes");
        open(file) ;
        print("Opened file: " + list[i]);

        // Extract the base name of the input file
        baseName = File.nameWithoutExtension;
        print(baseName);

                        // Actual imaging processing steps --> Change with your image processing steps
                        run("Duplicate...", "duplicate channels=2-3");

                        //z projection
                        run("Z Project...", "projection=[Max Intensity]");
                        rename("MAX" +  "/" + baseName);

                        //run("Brightness/Contrast...");
                        //select channel Gal8 mRuby
                        Stack.setChannel(1);
                        setMinAndMax(180, 1000);
                        run("Magenta");
                        //select channel Hoechst
                        Stack.setChannel(2);
                        setMinAndMax(250,1300);
                        run("Scale Bar...", "width=10 height=40 thickness=20 font=0 color=White background=None location=[Lower Right] horizontal bold overlay");
                        //outputFile = output_dir + "/" + baseName + "MAX";
        //saveAs("Tiff", outputFile);

                        //segment MFP488 dots
                        run("Duplicate...", "duplicate channels=1");
                        //run("Add Selection...");
                        //run("Gaussian Blur...", "sigma=1");
```

```
                    //run("Threshold...");
                    setThreshold(700, 65535, "raw");
                    setOption("BlackBackground", true);
                    run("Convert to Mask");
                    run("Watershed");
                    run("Set Measurements...", "area mean standard centroid add redirect=None decimal=2");
                    run("Analyze Particles...", "size=0.02-200 circularity=0.6-1.00 display add");


                    //measure Gal8 signal in segemented objects
                    selectWindow("MAX" +  "/" + baseName);
                    Stack.setChannel(1);
                    roiManager("Show None");
                    roiManager("Show All");
                    run("Clear Results");
                    roiManager("Measure");

        outputFile = output_dir + "/" + baseName + "Gal8 objects.csv";
        saveAs("Results", "outputFile");
                    outputFile = output_dir + "/" + baseName + "Gal8 objects.zip";
        roiManager("Save", outputFile);
        outputFile = output_dir + "/" + baseName + "MAX";
        saveAs("Tiff", outputFile);
        rename("MAX" + baseName);

                    run("Clear Results");
                    roiManager("Delete");
        // Close the images
        run("Close All");

       // Check if any images are still open and print a message
                    if (isOpen("Any")) {
                    print("There are still open images.");
                    } else {
                            print("All images closed.");
                    }
    }
}
```

Macro 2. Nuclei count batch processing

```
// Make a list of the files in the input directory
list = getFileList(dir);

// Loop through the list of files
for (i = 0; i < list.length; i++) {
    file = dir + "/" + list[i];
```

```
if (File.isDirectory(file) == 0 &&
    endsWith(list[i], ".tif")) {

    // Open the image and print the files in list to check if the correct files are opened
    open(file) ;
    print("Opened file: " + list[i]);

    // Extract the base name of the input file
    baseName = File.nameWithoutExtension;
    print(baseName);

                        // Actual imaging processing steps --> Change with your image processing steps
                        run("Duplicate...", "duplicate channels=3");

                        rename("Hoechst");
                        setMinAndMax(260, 500);
                        //run("Grays");
                        run("Gaussian Blur...", "sigma=2");
                        setThreshold(300, 65535, "raw");
                        setOption("BlackBackground", true);
                        run("Convert to Mask");
                        //reduce noise with median (It replaces each pixel's value with the median value of the intensities in the neighborhood around that pixel.)
                        //run("Median...", "radius=2");
                        //run("Subtract Background...", "rolling=200 sliding disable");
                        //setAutoThreshold("Default dark");

                        run("Fill Holes");
                        run("Set Measurements...", "area mean min integrated display add redirect=None decimal=3");
                        run("Analyze Particles...", "size=50-Infinity show=Masks display clear include add composite");
                        selectWindow("Mask of Hoechst");
                        rename("Nucleus Mask");

    // Save the processed image according to baseName
    selectWindow("Nucleus Mask");
    outputFile = output_dir + "/" + baseName + "_Nucleus_mask";
    saveAs("Tiff", outputFile);

    selectWindow("Hoechst");
    outputFile = output_dir + "/" + baseName + "Hoechst";
    saveAs("Tiff", outputFile);
    outputFile = output_dir + "/" + baseName + "Nuclei count.csv";
    saveAs("Results", "outputFile");

    run("Clear Results");
                        roiManager("Delete");

    // Close the images
    run("Close All");
```

```
        // Check if any images are still open and print a message
                        if (isOpen("Any")) {
                        print("There are still open images.");
                        } else {
                                print("All images closed.");
                        }
    }
}
```

Macro 3. Gal8-mRuby3 dots count batch processing (with TFAMoplexes)

```
// Make a list of the files in the input directory
list = getFileList(dir);

// Loop through the list of files
for (i = 0; i < list.length; i++) {
    file = dir + "/" + list[i];

    if (File.isDirectory(file) == 0 &&
        endsWith(list[i], ".tif")) {
         // Open the image and print the files in list to check if the correct files are opened
        open(file) ;
        print("Opened file: " + list[i]);

        // Extract the base name of the input file
        baseName = File.nameWithoutExtension;
        print(baseName);

        rename("MAX" +  "/" + baseName);

                        //run("Brightness/Contrast...");
                        //select channel Gal8 mRuby
                        Stack.setChannel(1);
                        setMinAndMax(180, 1000);
                        //run("Magenta");
                        //select channel Hoechst
                        Stack.setChannel(3);
                        setMinAndMax(250,1300);
                        //outputFile = output_dir + "/" + baseName + "MAX";
        //saveAs("Tiff", outputFile);

                        //segment Gal8 mRuby objects
                        run("Duplicate...", "duplicate channels=1");
                        //run("Add Selection...");
                        //run("Gaussian Blur...", "sigma=1");

                        //run("Threshold...");
                        setThreshold(1000, 65535, "raw");
```

```
                        setOption("BlackBackground", true);
                        run("Convert to Mask");
                        run("Watershed");
                        run("Set Measurements...", "area mean standard centroid add redirect=None decimal=2");
                        run("Analyze Particles...", "size=0.02-200 circularity=0.6-1.00 display add");


                        //measure Gal8 signal in segemented objects
                        selectWindow("MAX" +  "/" + baseName);
                        Stack.setChannel(1);
                        roiManager("Show None");
                        roiManager("Show All");
                        run("Clear Results");
                        roiManager("Measure");

        outputFile = output_dir + "/" + baseName + "Gal8 objects.csv";
        saveAs("Results", "outputFile");
                        outputFile = output_dir + "/" + baseName + "Gal8 objects.zip";
        roiManager("Save", outputFile);
        outputFile = output_dir + "/" + baseName + "MAX";
        saveAs("Tiff", outputFile);
        rename("MAX" + baseName);

                        run("Clear Results");
                        roiManager("Delete");
        // Close the images
        run("Close All");
       // Check if any images are still open and print a message
                        if (isOpen("Any")) {
                        print("There are still open images.");
                        } else {
                              print("All images closed.");
                        }
    }
}
```

Macro 4. MFP488-Gal8-mRuby3 colocalization analysis batch processing (with TFAMoplexes)

```
// Make a list of the files in the input directory
list = getFileList(dir);

// Loop through the list of files
for (i = 0; i < list.length; i++) {
    file = dir + "/" + list[i];

    // Check if the file is not a directory and is a nd2 file --> can be changed for any file type
    if (File.isDirectory(file) == 0 &&
        endsWith(list[i], ".nd2")) {
```

```
// Open the image and print the files in list to check if the correct files are opened
open(file) ;
print("Opened file: " + list[i]);

// Extract the base name of the input file
baseName = File.nameWithoutExtension;
print(baseName);

        // Actual imaging processing steps --> Change with your image processing steps
        run("Duplicate...", "duplicate channels=1-3");

        //z projection
        run("Z Project...", "projection=[Max Intensity]");
        rename("MAX" +  "/" + baseName);
        Stack.setChannel(1);
        //run("Brightness/Contrast...");
        setMinAndMax(250, 1300);
        run("Magenta");
        //select channel AB 488
        Stack.setChannel(2);
        setMinAndMax(100, 3500);
        //select channel Hoechst
        Stack.setChannel(3);
        setMinAndMax(250,1300);
        run("Scale Bar...", "width=10 height=40 thickness=20 font=0 color=White background=None location=[Lower Right] horizontal bold overlay");
        //outputFile = output_dir + "/" + baseName + "MAX";
//saveAs("Tiff", outputFile);

        //segment MFP488 dots
        run("Duplicate...", "duplicate channels=2");
        //run("Add Selection...");
        run("Gaussian Blur...", "sigma=1");

        //run("Threshold...");
        setThreshold(1000, 65535, "raw");
        setOption("BlackBackground", true);
        run("Convert to Mask");
        run("Watershed");
        run("Set Measurements...", "area mean standard centroid add redirect=None decimal=2");
        run("Analyze Particles...", "size=0.02-100 circularity=0.6-1.00 display add");


        //measure MFP488 signal in segemented objects
        selectWindow("MAX" +  "/" + baseName);
        Stack.setChannel(2);
        roiManager("Show None");
        roiManager("Show All");
```

```
                        run("Clear Results");
                        roiManager("Measure");

          outputFile = output_dir + "/" + baseName + "MFP 488 objects.csv";
          saveAs("Results", "outputFile");
                        outputFile = output_dir + "/" + baseName + "MFP 488 objects.zip";
          roiManager("Save", outputFile);
          outputFile = output_dir + "/" + baseName + "MAX";
          saveAs("Tiff", outputFile);
          rename("MAX" + baseName);

                        //measure Gal8-mRuby3 signal in segemented objects
                        selectWindow("MAX" + baseName);
                        Stack.setChannel(1);
                        roiManager("Show None");
                        roiManager("Show All");
                        run("Clear Results");
                        roiManager("Measure");


          outputFile = output_dir + "/" + baseName + "mRuby3.csv";
          saveAs("Results", outputFile);
                        run("Clear Results");
                        roiManager("Delete");


          // Close the images
          run("Close All");

         // Check if any images are still open and print a message
                        if (isOpen("Any")) {
                        print("There are still open images.");
                        } else {
                                   print("All images closed.");
                        }
     }
 }
```
